# Uptake of Home-Based HIV Testing, Linkage to Care, and Community Attitudes about ART in Rural KwaZulu-Natal, South Africa: Descriptive Results from the First Phase of the ANRS 12249 TasP Cluster-Randomised Trial

**DOI:** 10.1371/journal.pmed.1002107

**Published:** 2016-08-09

**Authors:** Collins C. Iwuji, Joanna Orne-Gliemann, Joseph Larmarange, Nonhlanhla Okesola, Frank Tanser, Rodolphe Thiebaut, Claire Rekacewicz, Marie-Louise Newell, Francois Dabis, ANRS 12249 TasP trial group

**Affiliations:** 1 Africa Centre for Population Health, University of KwaZulu-Natal, Durban, South Africa; 2 Research Department of Infection and Population Health, University College London, London, United Kingdom; 3 Centre INSERM U1219 Bordeaux Population Health, Université de Bordeaux, Bordeaux, France; 4 Institut de Santé Publique, d’Epidémiologie et de Développement, Centre INSERM U1219 Bordeaux Population Health, Université de Bordeaux, Bordeaux, France; 5 Centre Population & Développement UMR 196, Université Paris Descartes, Institut de Recherche pour le Développement, Paris, France; 6 School of Nursing and Public Health, University of KwaZulu-Natal, Durban, South Africa; 7 Agence Nationale de Recherches sur le Sida et les Hépatites Virales, Paris, France; 8 Human Health and Development and Global Health Research Institute, Faculty of Medicine, University of Southampton, Southampton, United Kingdom; University of Bern, SWITZERLAND

## Abstract

**Background:**

The 2015 WHO recommendation of antiretroviral therapy (ART) for all immediately following HIV diagnosis is partially based on the anticipated impact on HIV incidence in the surrounding population. We investigated this approach in a cluster-randomised trial in a high HIV prevalence setting in rural KwaZulu-Natal. We present findings from the first phase of the trial and report on uptake of home-based HIV testing, linkage to care, uptake of ART, and community attitudes about ART.

**Methods and Findings:**

Between 9 March 2012 and 22 May 2014, five clusters in the intervention arm (immediate ART offered to all HIV-positive adults) and five clusters in the control arm (ART offered according to national guidelines, i.e., CD4 count ≤ 350 cells/μl) contributed to the first phase of the trial. Households were visited every 6 mo. Following informed consent and administration of a study questionnaire, each resident adult (≥16 y) was asked for a finger-prick blood sample, which was used to estimate HIV prevalence, and offered a rapid HIV test using a serial HIV testing algorithm. All HIV-positive adults were referred to the trial clinic in their cluster. Those not linked to care 3 mo after identification were contacted by a linkage-to-care team. Study procedures were not blinded.

In all, 12,894 adults were registered as eligible for participation (5,790 in intervention arm; 7,104 in control arm), of whom 9,927 (77.0%) were contacted at least once during household visits. HIV status was ever ascertained for a total of 8,233/9,927 (82.9%), including 2,569 ascertained as HIV-positive (942 tested HIV-positive and 1,627 reported a known HIV-positive status). Of the 1,177 HIV-positive individuals not previously in care and followed for at least 6 mo in the trial, 559 (47.5%) visited their cluster trial clinic within 6 mo. In the intervention arm, 89% (194/218) initiated ART within 3 mo of their first clinic visit. In the control arm, 42.3% (83/196) had a CD4 count ≤ 350 cells/μl at first visit, of whom 92.8% initiated ART within 3 mo. Regarding attitudes about ART, 93% (8,802/9,460) of participants agreed with the statement that they would want to start ART as soon as possible if HIV-positive. Estimated baseline HIV prevalence was 30.5% (2,028/6,656) (95% CI 25.0%, 37.0%). HIV prevalence, uptake of home-based HIV testing, linkage to care within 6 mo, and initiation of ART within 3 mo in those with CD4 count ≤ 350 cells/μl did not differ significantly between the intervention and control clusters. Selection bias related to noncontact could not be entirely excluded.

**Conclusions:**

Home-based HIV testing was well received in this rural population, although men were less easily contactable at home; immediate ART was acceptable, with good viral suppression and retention. However, only about half of HIV-positive people accessed care within 6 mo of being identified, with nearly two-thirds accessing care by 12 mo. The observed delay in linkage to care would limit the individual and public health ART benefits of universal testing and treatment in this population.

**Trial registration:**

ClinicalTrials.gov NCT01509508

## Abstract 2

Collins Iwuji and colleagues report implementation indicators and early health outcomes from the first phase of a cluster-randomized trial of immediate antiretroviral therapy to all HIV-positive individuals in rural KwaZulu-Natal, South Africa.

## Author Summary

**Why Was This Study Done?:**

- A study in stable sexual partners in which one partner was HIV-positive and the other partner was HIV-negative (and both partners had disclosed to each other) showed that if the HIV-positive partner was on antiretroviral therapy, there was a 96% reduction in HIV transmission from the HIV-positive partner to the HIV-negative partner.
- However, we do not know if antiretroviral therapy prescribed to HIV-positive individuals in the general population—and where individuals might not disclose their HIV status to sexual partners—would have a similar impact on HIV transmission.
- It is important to determine whether prescribing antiretroviral therapy to all HIV-positive individuals is more effective at decreasing HIV transmission than starting individuals on antiretroviral therapy only once their HIV has progressed to the point at which local HIV treatment guidelines currently recommend that HIV-positive individuals start treatment.

**What Did the Researchers Do and Find?:**

- We designed an experiment to investigate whether antiretroviral therapy can reduce new HIV infections in the general population, and piloted the trial in ten communities in KwaZulu-Natal, South Africa, to check whether starting HIV-positive individuals on antiretroviral therapy directly after diagnosis is feasible and acceptable.
- We visited people in their homes, offered HIV rapid tests every six months to all individuals 16 years and older, and referred identified HIV-positive individuals to trial clinics, where they were offered antiretroviral therapy either regardless of their CD4 count (intervention group) or when they were treatment-eligible per current national guidelines (control group).
- During the two-year study, we contacted 9,927 (77%) of 12,894 eligible individuals and ascertained the HIV status of 80% of contacted women and 75% of contacted men.
- HIV-positive status was ascertained for 1,339 adults who were not previously in care; 1,177 were followed in the trial at least 6 mo after referral, of whom 559 (47.5%) engaged with care within this period.

**What Do These Findings Mean?:**

- Our findings show good acceptance of home-based HIV testing in rural South Africa but highlight the challenges in reaching adequate numbers of people to offer HIV tests to, especially among men.
- We also found that linkage to care was slower than expected, but amongst those who reached the clinics, uptake of antiretroviral therapy was high, with the majority of individuals achieving good control of the virus.
- Our study informs health care professionals, planners, and policy makers about the challenges that need to be addressed to achieve the UNAIDS target of 90% of people living with HIV aware of their HIV diagnosis, 90% on antiretroviral therapy, and 90% achieving good control of the virus, with testing and treatment offered to all.

## Introduction

Although significant gains have been made in the control of the HIV epidemic in many sub-Saharan countries, the annual number of new HIV infections remains unacceptably high [1]. Approximately 6.3 million people were estimated to be living with HIV in South Africa alone in 2013, of whom 3.1 million were on antiretroviral therapy (ART) [2]. Adult HIV prevalence in KwaZulu-Natal province in 2011 was estimated to be as high as 30% in peri-urban communities [3], making this province an ideal setting to evaluate the impact of universal test and treat on HIV incidence.

HIV viral load (VL) in HIV-positive individuals is the dominant determinant of transmission [4]. Effective ART lowers VL and thus substantially decreases the risk of HIV transmission. In heterosexual couples in stable relationships, ART provided to the HIV-positive partner with T cell lymphocyte CD4+ count between 250 and 550 cells/μl reduced transmission to the HIV-negative partner by 96% [5]. Repeat annual population-based HIV surveys in rural KwaZulu-Natal, South Africa, have shown that individual-level HIV acquisition risk decreased by 38% when ART coverage in the surrounding community increased from <10% to 30%–40% under national treatment guidelines (first ART initiation at a CD4 count of ≤200 cells/μl, then at ≤350 cells/μl) [3]. The impact on HIV transmission at the population level of ART initiation in all HIV-positive individuals soon after HIV diagnosis has not yet been evaluated in a trial setting.

Mathematical models have suggested that significant reductions in HIV transmission could be achieved with optimisation of every step of the HIV care cascade—starting with high uptake of regular HIV testing in all adults—and with immediate treatment initiation in those found to be HIV-positive [6]. The Joint United Nations Programme on HIV/AIDS (UNAIDS) has set targets that by 2020, 90% of all people living with HIV will know their HIV status, 90% of all people with diagnosed HIV infection will receive sustained ART, and 90% of all people receiving ART will achieve viral suppression (the 90-90-90 targets) [7]. However, the impact of the HIV care cascade on HIV incidence would additionally depend on the context of the sexual networks (such as heterogeneity, concurrency, and mixing) in which HIV transmission occurs [8–10].

Experience from public health HIV treatment programmes highlights the challenges of reaching high uptake of HIV testing, linkage to care, ART initiation, and long-term treatment adherence [11]. A recent meta-analysis of 28 studies that evaluated several approaches to community-based HIV testing including door-to-door HIV testing amongst 555,267 participants reported an HIV test acceptance rate of 80% (95% CI 76.9%, 83.1%), with higher HIV test acceptance in community-based than in facility-based programmes, although the former identified fewer HIV-positive people and with substantially less advanced disease [12]. There are limited data on repeat HIV testing, but one population-based study in rural Malawi reported repeat HIV testing uptake of 96% amongst participants who tested HIV-negative in a previous survey and were recontacted [13]. Linkage to care and ART initiation present further challenges in the HIV care cascade, with results from a further meta-analysis of sub-Saharan African data showing that for every 100 patients with a positive HIV test, 72 had a CD4 count performed, 40 were deemed ART-eligible by national treatment criteria, and only 25 started ART [14]. However, somewhat more positively, a recent evaluation of self-testing for HIV and linkage to care in Blantyre, Malawi, reported that 56% of individuals who tested HIV-positive linked to care within 12 mo [15]. There are little, if any, data on the acceptability and uptake of immediate ART for HIV prevention in African populations [16,17].

In early 2012, we initiated a cluster-randomised trial in rural KwaZulu-Natal to evaluate whether immediate ART in HIV-positive individuals could significantly reduce HIV incidence at the population level. At the time of implementation, similar trials were still in their planning phase [18,19]; with the complexity of implementing such a large trial and a lack of available data to inform the design and sample size, we opted to start the interventions in a limited number of clusters randomised for the main trial. We were able to evaluate process indicators such as uptake of initial and repeat home-based HIV testing, linkage to care, uptake of ART, and community attitudes and beliefs about HIV, which are important for the success of the main trial as well as for HIV treatment programmes more generally.

## Methods

### Ethics Statement

The trial was approved by the Biomedical Research Ethics Committee (BFC 104/11) at the University of KwaZulu-Natal and the Medicines Control Council of South Africa. (ClinicalTrials.gov: NCT01509508; South African National Clinical Trials Register: DOH-27-0512-3974). All participants provided written or witnessed thumbprint informed consent.

### Study Design

ANRS 12249 TasP is a cluster-randomised trial including 22 clusters (2 × 11) at full implementation. The full trial protocol has been published previously [20]. The protocol underwent some modification in response to changes in South African national ART guidelines and to optimise trial implementation procedures by introducing an active linkage-to-care team in both arms in May 2013 to facilitate linkage to trial clinics for those not linked to care within 3 mo of being referred. These amendments were approved by the Biomedical Research Ethics Committee of the University of KwaZulu-Natal and the trial data and safety monitoring board.

We here present results on process indicators from the first phase of the trial in ten (2 × 5) clusters; four (2 × 2) of the ten clusters began enrolment on 9 March 2012, and the remaining six (2 × 3) on 22 January 2013. A total of three HIV survey rounds were conducted in the initial four clusters between 9 March 2012 and 31 August 2013, and two in the remaining six clusters between 22 January 2013 and 5 April 2014. Follow-up for all those identified as HIV-positive and receiving care in trial clinics started on 9 March 2012, depending on cluster implementation date, and ended on 22 May 2014.

### Trial Setting

This cluster-randomised trial was implemented in the Hlabisa sub-district of the uMkhanyakude district in northern KwaZulu-Natal, South Africa. The area is largely rural, with scattered homesteads and a national road on the boundary, adjacent to the Africa Centre for Population Health and its demographic surveillance area. It is served by the Hlabisa Department of Health HIV treatment and care programme [21].

### Study Procedures

The study procedures and instruments have been fully described previously [20,22]. Procedures relevant to the first phase of the trial are summarised below.

#### Home-based procedures

In each home-based HIV testing and survey round, conducted every 6 mo, homestead (a bounded physical structure usually comprising one household but occasionally two or more households with different household heads) visits took place between 8:00 a.m. and 4:30 p.m. weekly from Tuesday to Saturday. Where required, separate homestead visits took place between 10:00 a.m. and 6:30 p.m. from Thursday to Sunday to accommodate adults not contacted during the standard homestead visit times, mainly students and employed individuals.

Individuals were eligible for trial participation if they were aged ≥16 y and resident (defined as spending four or more nights per week) members of a household in the designated cluster. Individuals were ineligible if they did not fulfil the criteria for residency or lacked the mental capacity to give informed consent.

Verbal consent from the homestead owner was obtained before entering any homestead. After explaining the study, each household head was asked to enumerate all eligible household members and complete a household asset form. Eligible individuals present on the day were taken to a private area and consented in writing to respond to a social and sexual behaviour questionnaire and give a finger-prick blood sample, collected on filter paper as dried blood spots (DBSs). An individual was considered a trial participant if they agreed to complete a study questionnaire, although opting out of providing DBSs was accepted. DBS samples collected longitudinally during survey rounds were tested using HIV ELISA in the Africa Centre for Population Health laboratory in Durban for identification of new cases of HIV infection, which is the primary outcome of the main trial. Researchers and study participants were blinded to these longitudinal DBS results.

HIV pretest counselling and a finger-prick rapid HIV test were offered, following a separate written consenting process, to individuals who completed the study questionnaire, using a serial HIV testing algorithm [23]. HIV 1/2 Gold Screening Test (G-Ocean, Hong Kong, China) was used as the first test, for screening, and Alere Determine HIV-1/2 (Alere, Kempton Park, South Africa) was used as the second test, for confirmation of HIV-positive results, in line with the provincial Department of Health (DoH) protocol. This protocol was later amended on 20 September 2012 to Alere Determine HIV-1/2 for screening and First Response HIV-1/HIV-2 test kit (Premier Medical Corporation, Kachigam, India) for confirmation, following changes in the DoH directive.

The result of the rapid HIV test was given to each participant, with counselling as appropriate, and further psychological support was offered for any serious distress observed. Those who newly tested HIV-positive or who self-reported being HIV-positive were referred to the TasP trial clinic in their cluster. Those who self-reported being HIV-negative were documented as having refused to be tested.

Procedures offered to eligible individuals were the same in each repeat survey round, irrespective of their HIV status recorded in the previous round.

#### TasP trial clinic procedures

HIV-positive participants attending TasP trial clinics were asked to provide written consent to (i) complete clinical history and examination questionnaires and provide blood specimens for VL testing and (ii) receive care as per national guidelines and ART as per cluster allocation. HIV-positive participants comprised those newly diagnosed as HIV-positive and those who self-reported as HIV-positive during home-based survey visits; they could be either ART-naïve or ART-experienced from their previous HIV care provider.

All consenting HIV-positive participants underwent clinical evaluation and a point-of-care CD4 measurement (Alere Pima CD4 test, Alere, Waltham, MA, US). All participants eligible for ART attended adherence and ART literacy sessions and initiated ART within 2 wk of the baseline visit, or sooner if severely immunocompromised. A fixed dose combination of tenofovir/emtricitabine/efavirenz (Atripla) was used for first-line ART, except if a participant’s clinical condition indicated otherwise. Second-line ART was informed by the results of genotypic resistance tests in participants failing first-line ART.

Participants receiving ART underwent monthly clinical evaluation including scheduled safety monitoring blood and HIV VL measurements (Abbott m2000 RealTime System, Abbott Molecular, Des Plaines, IL, US) at the first visit, at 3 and 6 mo after ART initiation, and every 6 mo thereafter. They were also interviewed for clinical adverse events. Unscheduled clinic visits were also allowed for participants with clinical complaints. In the control clusters, patients not yet eligible for ART were invited to return to the study clinic in 4 to 6 mo for pre-ART care, positive prevention services, repeat clinical assessment, and CD4 count measurement. Participants missing a trial clinic appointment were phoned, and, when possible, a new appointment was scheduled. Participants who failed to link to care 3 mo after being referred by the field team were contacted by a trial linkage-to-care team either by phone or through a home visit.

### Intervention

In the intervention clusters, HIV-positive individuals were informed during home-based HIV testing that they would be provided ART irrespective of CD4 count and clinical stage. In the control clusters, HIV-positive individuals were informed that ART would be offered according to the criteria of the national South African guidelines: CD4 count ≤ 350 cells/μl, World Health Organization (WHO) clinical stage 3 or 4, or coinfection with multidrug resistant or extensively drug-resistant tuberculosis.

### Definition of Variables and Outcomes

S1 Table presents the outcomes measured in phase 1 and presented here, and those that will be measured after trial completion.

HIV prevalence was estimated on the basis of antibody test results from the DBSs collected during the first survey round only. We estimated ART coverage at the start of the trial (proportion of all HIV-positive individuals on ART) among those with positive DBS results (first survey round) using linked information from DoH clinics and pharmacy records (ARTemis and iDART databases). Matching between the three databases was based on first names, last name, date of birth, South African ID number, and cell phone numbers.

Means and medians (interquartile ranges [IQRs]) for age at registration and other demographic characteristics were computed among individuals who completed at least one individual questionnaire at home.

Proportion of individuals contacted and whose HIV status was ascertained was computed per home-based survey round, i.e., an individual eligible in three survey rounds (taking into account round of registration and population exits), fully contacted in two rounds, but accepting an HIV rapid test only in one round will contribute three episodes in the denominator and two episodes in the numerator for estimation of contact, and two episodes in the denominator and one in the numerator for HIV ascertainment. The HIV status of an individual was ascertained if that person accepted an HIV rapid test and obtained a valid result (i.e., invalid/indeterminate results excluded) or if he/she self-reported being HIV-positive.

Rapid HIV test uptake was computed amongst individuals who were contacted, did not self-report being HIV-positive, and accepted a rapid HIV test.

Linkage to care within 6 mo was computed among individuals who were ascertained as HIV-positive at home, not previously in care (in DoH clinics in the study area), and observed at least 6 mo (taking into account population exits and the end date of data collection). Linkage to care was defined as having a first clinic visit either in a DoH clinic in the study area or a TasP clinic and was obtained by individual linkage in the databases as described above.

ART uptake within 3 mo of the first clinic visit was computed in TasP clinics only, among participants not on ART at the first clinic visit, regardless of ART eligibility criteria. ART uptake was further stratified by CD4 count at first visit in the TasP clinics.

Viral suppression was defined as having VL < 400 copies/ml on ART.

We estimated the status within the HIV care cascade (diagnosed, ever on ART, ever virally suppressed during the trial follow-up) of trial participants who were contacted and identified to be HIV-positive (through DBS results and/or HIV rapid test) who linked to TasP or DoH clinics, using linked information from DoH clinics. In order to present a population cascade, we also estimated the number of HIV-infected individuals who had not been reached by the trial field activities by applying the observed HIV prevalence (from DBS results) to the total population of registered individuals. This assumes there was no selection bias.

Attitude indicators were computed (i) among individuals who completed at least one individual questionnaire at home (the first questionnaire was used for individuals who completed several questionnaires over the trial) or (ii) among HIV-positive participants who linked to TasP clinics, at their first visit, if they were not already on ART at this first clinic visit.

### Sample Size

The main trial at full implementation was 80% powered to detect an overall 34% reduction in cumulative HIV incidence over 4 y (*n* = 22,000; 22 clusters), with an incidence of 2.25% per year in the control clusters. The calculation made allowance for 20% loss to follow-up and assumed a coefficient of variation of 0.25 to account for variation between clusters [20]. Assumptions for attaining this incidence reduction were that the level of population contact would need to be 90%, HIV status ascertainment 80%, linkage to care 70%, and baseline HIV prevalence 24%.

### Randomisation

Randomisation was performed by the trial statisticians before the start of the trial. 211 local areas were aggregated into 48 clusters. Initial sample size calculation showed that 34 clusters (2 × 17) would be required, and these were randomly allocated to the two arms, control and intervention. Randomisation was carried out within each stratum to derive an equal number of control and intervention communities per stratum. Random number generation and the randomisation procedure were performed in MapInfo version 11.0. The sample size was subsequently amended to 2 × 11 with an increase in the duration of follow-up through a revision of the protocol for the main trial. For this initial phase, only ten (2 × 5) of the 22 clusters were used. To minimise the degree of between-cluster variation, clusters were stratified on the basis of predicted HIV prevalence, extrapolating from HIV surveillance data from the Africa Centre for Population Health’s demographic surveillance area and data from antenatal clinics (six strata).

### Statistical Analyses

Process indicators were summarised by arm and described according to key baseline characteristics (sex, age, education level, marital status, and professional status). They were then compared by arm using Pearson’s chi² test with Rao-Scott second-order correction, which is appropriate in the context of cluster sampling [24]. The *p*-values were computed with a Satterthwaite approximation to the distribution and with denominator degrees of freedom as recommended by Thomas and Rao [25] or with a design-based *t*-test (taking into account clustering for variance computation).

## Results

### Registration and Enrolment of Participants

**Fig 1:**
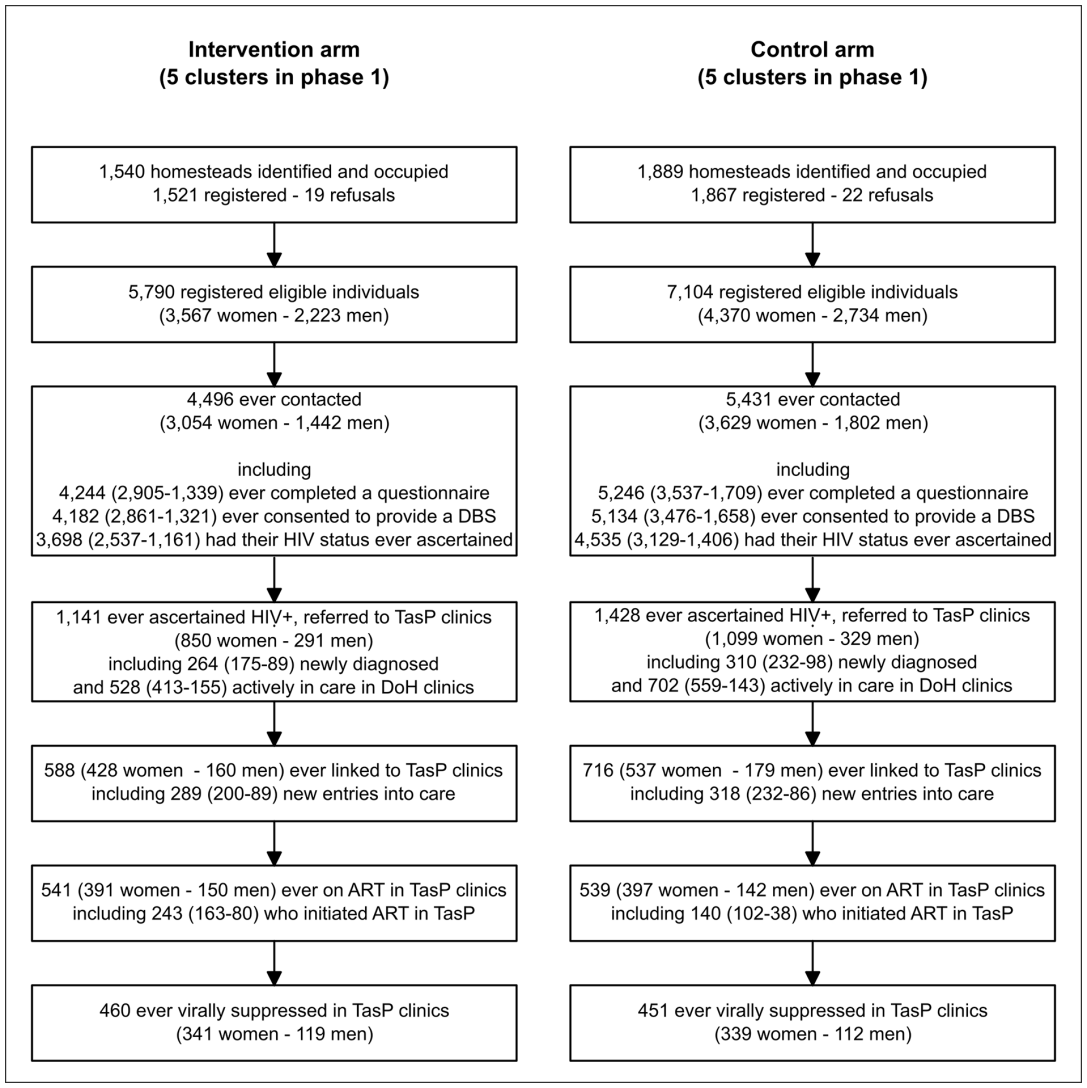
Flow diagram of enrolment by trial arm and sex in the ANRS 12249 TasP trial.

Three consecutive rounds of home-based HIV testing were conducted in four initial clusters (2 × 2) starting 9 March 2012, and two rounds in six additional clusters (2x3) starting 22 January 2013. A total of 12,894 individuals were registered as eligible between 9 March 2012 and 22 May 2014 (Fig 1). During this period, 9,927 (77.0%) were contacted by the fieldworkers at least once, with no difference between arms (77.7% in the intervention arm and 76.4% in the control arm). Of the 9,927 individuals ever contacted, 9,490 (95.6%) agreed to complete a social and sexual behaviour questionnaire at least once, and HIV status was ascertained at least once for 82.3% (3,698/4,496) and 83.5% (4,535/5,431) in the intervention and control arms, respectively (Fig 1); this translates to ascertained HIV status for 63.9% (3,698/5,790) and 63.8% (4,535/7,104) of all registered individuals, respectively.

Overall, the HIV prevalence was 30.5% (95% CI 25.0%, 37.0%).

### Baseline Characteristics

The median age (IQR) of participants was 32.3 y (22.1–52.4), and the majority were female (67.9%). A third of participants had primary education or less. The majority were never married; very few were formally employed. No difference was observed between arms (Table 1).

**Table 1:** Baseline characteristics of participants in intervention and control arms, first phase of the TasP ANRS 12249 trial, 2012–2014.

Characteristic	Intervention Arm	Control Arm	*p*-Value
Number of registered individuals	5,790	7,104	
Estimated baseline HIV prevalence (*n/N*)	29.0% (880/3,036)	31.7% (1,148/3,620)	0.672
Estimated baseline ART coverage (*n/N*)	36.0% (317/880)	39.8% (457/1,148)	0.271
Number of participants who completed at least one questionnaire	4,244	5,246	
Female	68.4%	67.4%	0.486
Mean age (years) at registration	39.0	38.1	0.944
Median age (IQR)	33.3 (22.4–53.3)	31.6 (21.9–51.8)	
Never been married	67.0%	69.1%	0.678
Primary education or less	42.6%	39.7%	0.133
Employed	9.4%	9.9%	0.842
Condom use at last sexual act (IQ1)	29.3%	32.2%	0.342

*p*-Values correspond to Pearson’s chi² tests with Rao-Scott second-order correction or design-based *t*-tests (taking into account clustering) for a difference between the intervention and control arms.

IQ1, first administered questionnaire at home.

### Contact and Uptake of HIV Testing per Round

The proportion of registered individuals contacted per survey round was 66.8%, similar in both arms (*p* = 0.530) but lower in males than females (53.3% versus 75.2%). Uptake of rapid HIV test at first contact was 73.1% overall, similar in both arms (Table 2). Amongst those contacted, HIV status ascertainment (rapid HIV test uptake plus self-reported HIV-positive) per survey round was 77.6%, also similar between arms (*p* = 0.676) (Table 2).

**Table 2:** Process indicators by trial arm and by sex, first phase of the ANRS 12249 TasP trial, 2012–2014.

Process Indicator	Women	Men	Overall	Difference in Proportions for Women versus Men	
				Percent (95% CI)	*p*-Value
**Intervention arm, percent (*n/N*)**					
Contact per home-based survey round	74.4% (5,554/7,465)	50.3% (2,305/4,587)	65.2% (7,859/12,052)	24.1 (21.4, 26.9)	<0.001
HIV ascertainment per home-based survey round	76.8% (4,264/5,554)	76.6% (1,765/2,305)	76.7% (6,029/7,858)	0.2 (−4.1, 4.5)	0.932
Linkage to care within 3 mo (individuals not in care at referral)	36.7% (159/433)	38.1% (67/176)	37.1% (226/609)	−1.3 (−9.7, 7.0)	0.770
Linkage to care within 6 mo (individuals not in care at referral)	47.4% (188/397)	48.2% (79/164)	47.6% (267/561)	−0.8 (−5.0, 3.3)	0.724
Linkage to care within 12 mo (individuals not in care at referral)	62.6% (134/214)	62.6% (57/91)	62.6% (191/305)	0.0 (−3.4, 3.4)	0.991
ART initiation within 3 mo of first clinic visit (CD4 count ≤ 350 cells/μl at baseline)	95.0% (57/60)	85.0% (34/40)	91.0% (91/100)	10.0 (−2.9, 22.9)	0.073
ART initiation within 3 mo of first clinic visit (CD4 count > 350 cells/μl at baseline)	89.8% (79/88)	80.0% (24/30)	87.3% (103/118)	9.8 (−3.5, 23.1)	0.168
**Control arm, percent (*n/N*)**					
Contact per home-based survey round	75.8% (6,426/8,475)	56.0% (2,904/5,189)	68.3% (9,330/13,664)	19.8 (16.6, 23.1)	<0.001
HIV ascertainment per home-based survey round	80.4% (5,165/6,426)	73.9% (2,146/2,904)	78.4% (7,311/9,326)	6.5 (3.9, 9.0)	0.009
Linkage to care within 3 mo (individuals not in care at referral)	35.6% (190/534)	39.3% (72/183)	36.5% (262/717)	−3.4 (−7.8, 0.3)	0.137
Linkage to care within 6 mo (individuals not in care at referral)	47.3% (218/461)	47.7% (74/155)	47.4% (292/616)	−0.4 (−4.5, 3.6)	0.838
Linkage to care within 12 mo (individuals not in care at referral)	62.9% (146/232)	66.1% (39/59)	63.6% (185/291)	−3.2 (−17.9, 11.5)	0.700
ART initiation within 3 mo of first clinic visit (CD4 count ≤ 350 cells/μl at baseline)	93.3% (56/60)	91.3% (21/23)	92.8% (77/83)	2.0 (−6.3, 10.3)	0.587
ART initiation within 3 mo of first clinic visit (CD4 count > 350 cells/μl at baseline)	9.8% (9/92)	14.3% (3/21)	10.6% (12/113)	−4.5 (−21.4, 12.4)	0.565
**Difference in proportions for intervention versus control arm, percent (95% CI), *p*-value**					
Contact per home-based survey round	−1.4 (−9.1, 6.3), 0.730	−5.7 (−17.1, 5.6), 0.353	−3.1 (−12.1, 6.0), 0.530		
HIV ascertainment per home-based survey round	−3.6 (−11.7, 4.5), 0.386	2.7 (−5.0, 10.4), 0.525	−1.6 (−9.3, 6.0), 0.676		
Linkage to care within 3 mo (individuals not in care at referral)	1.1 (−11.8, 14.1), 0.866	−1.3 (−9.4, 6.9), 0.525	0.6 (−10.4, 11.6), 0.921		
Linkage to care within 6 mo (individuals not in care at referral)	0.1 (−10.4, 10.5), 0.990	0.4 (−8.7, 9.6), 0.929	0.2 (−9.5, 9.9), 0.970		
Linkage to care within 12 mo (individuals not in care at referral)	0.3 (−16.6, 16.0), 0.971	−3.5 (−25.5, 18.6), 0.766	−1.0 (−17.4, 15.5), 0.912		
ART initiation within 3 mo of first clinic visit (CD4 count ≤ 350 cells/μl at baseline)	1.7 (−3.9, 7.2), 0.571	−6.3 (−24.5, 11.9), 0.518	−1.8 (−11.2, 7.7), 0.719		
ART initiation within 3 mo of first clinic visit (CD4 count > 350 cells/μl at baseline)	80.0 (73.2, 86.8), <0.001	65.7 (43.2, 88.2), 0.003	76.7 (68.4, 85.0), <0.001		

*p*-Values correspond to Pearson’s chi² tests with Rao-Scott second-order correction (taking into account clustering) for a difference between women and men or between the intervention and control arms. Proportion of individuals contacted and whose HIV status was ascertained was computed per home-based survey round, i.e., an individual eligible in three survey rounds, fully contacted in two rounds, but accepting a HIV rapid test only in one round will contribute three episodes in the denominator and two episodes in the numerator for estimation of contact, and two episodes in the denominator and one in the numerator for HIV ascertainment.

Repeat HIV test uptake was 85.3% at second contact in those testing HIV-negative at first contact. Cumulatively in all survey rounds, 2,569 adults were ascertained as HIV-positive (942 tested HIV-positive and 1,627 reported a known HIV-positive status) and referred to TasP clinics in their cluster (Fig 1).

Amongst the 1,694 individuals who were contacted at least once but whose HIV status was never ascertained, 573 (33.8%) did not provide any reason for refusal. Amongst the remaining 1,121 individuals, 545 (48.6%) ever reported that they thought they were HIV-negative, 353 (31.5%) were afraid to test, 96 (8.6%) would test only with their partner, and 323 (28.8%) provided other reasons (overall proportion differs from 100% due to multiple reasons per individual).

### Linkage to Care

Of the 2,569 adults ascertained as HIV-positive and referred to TasP clinics, 1,230 were actively engaged with care in DoH clinics at the time of referral (Fig 1). Amongst the remaining 1,339 adults, 1,177 were followed in the trial at least 6 mo after referral (Table 2). Of these, 559 (47.5%) linked to care within 6 mo of referral, with no significant difference between arms (*p* = 0.970). The corresponding estimate at 12 mo was 63.1% (376/596) overall, again not significantly different between arms (*p* = 0.912).

### Uptake of ART and Retention

In the intervention arm, among participants not already on ART who were followed at least 3 mo in TasP clinics, 103/118 (87.3%) participants with CD4 count > 350 cells/μl initiated ART within 3 mo of the first clinic visit (Table 2). There was no difference between arms in the proportion of treated individuals who achieved viral suppression (448/526, 85.2%, in the intervention arm and 440/518, 84.9%, in the control arm). The median (IQR) duration on ART was 265 d (162–383).

Retention in care in trial clinics at 12 mo was 84.4%, slightly higher in the intervention than in the control arm (86.2% versus 82.5%).

### HIV Care Cascade

**Fig 2:**
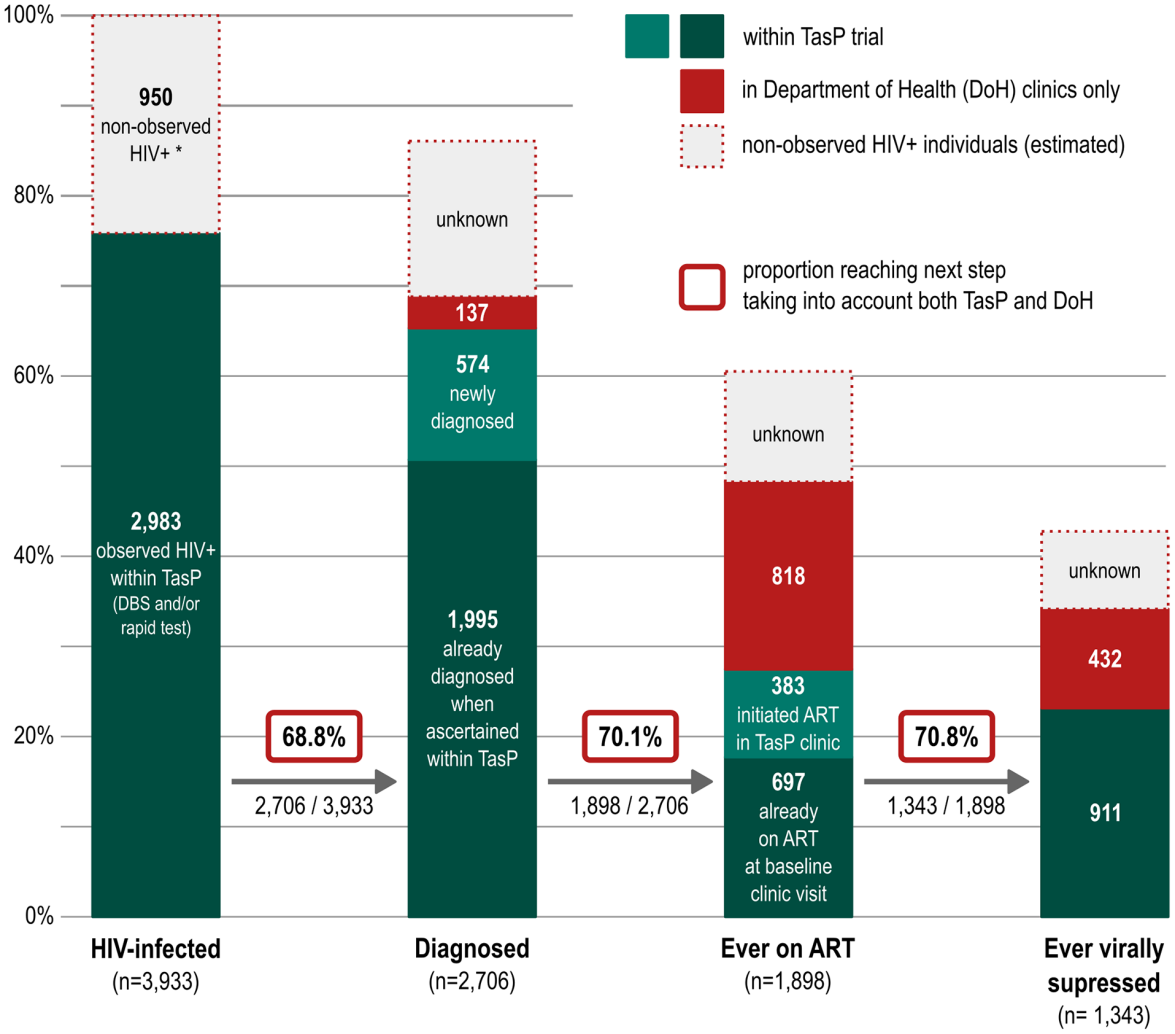
Estimated HIV care cascade among all HIV-infected individuals at the population level. *The number of non-observed HIV-positive individuals was estimated under the assumption that there was no selection bias, i.e., the observed HIV prevalence of 30.5% was applied to individuals whose HIV status was not observed within TasP. The 2,983 amongst the HIV-infected individuals in column 1 refers to the total number of individuals ascertained as HIV-positive within TasP in both arms of the trial, 2,569 (Fig 1), plus those who declined HIV rapid test but whose DBS HIV ELISA was positive and matched that of 137 individuals on ART within the DoH clinics.

Among an estimated total of 3,933 (30.5% HIV prevalence × 12,894) HIV-infected individuals ≥16 y living within the trial area, 2,706 (68.8%) were diagnosed (2,569 ever ascertained within TasP plus 137 not ascertained within TasP but ever in care in a DoH clinic), of whom 1,898 (70.1%) were ever in care in a TasP and/or DoH clinic, of whom 1,343 (70.8%) achieved viral suppression (Fig 2). Overall, 34.1% (1,343/3,933) of all HIV-positive individuals were ever virally suppressed.

### Attitude Indicators and Other Observations

Almost all participants in both arms were of the opinion that people should test regularly and agreed that they would start ART as soon as possible if diagnosed HIV-positive, with no difference by arm (Table 3).

**Table 3:** Attitudes towards repeat home-based HIV testing and early treatment by trial arm, first phase of the ANRS 12249 TasP trial, 2012–2014.

Attitude Indicator	Percent (*n/N*)		*p*-Value
	Intervention Arm	Control Arm	
Consider that people should test regularly (IQ1)	93.3% (3,961/4,244)	92.9% (4,876/5,246)	0.846
Consider that best place to get HIV tested is “at home” (IQ1)	60.4% (2,564/4,244)	56.5% (2,965/5,246)	0.505
Agree that would want to start ART as soon as possible if HIV-positive (IQ1)	93.1% (3,951/4,244)	92.5% (4,851/5,246)	0.688
Believe that antiretroviral drugs make people with HIV less infectious (IQ1)	78.7% (3,340/4,244)	75.3% (3,948/5,246)	0.472
Less worried about HIV now that treatments have improved (IQ1)	81.9% (3,476/4,244)	84.2% (4,419/5,246)	0.258
Agree that ART will reduce transmission to sexual partners (HIV+ patients, first visit in TasP clinic, not already on ART)	77.1% (219/284)	82.9% (252/304)	0.601

*p*-Values correspond to Pearson’s chi² tests with Rao-Scott second-order correction (taking into account clustering) for a difference between the intervention and control arms.

IQ1, first administered questionnaire at home.

One female participant newly identified as HIV-positive among the 6,155 individuals who underwent 10,445 HIV rapid tests in the home suffered an acute adjustment reaction with suicidal intent; she was referred to a clinical psychologist for support and care. There were no reports of study-related gender-based violence, partnership dissolution, or breach of confidentiality.

## Discussion

This 2-y initial phase of a trial evaluating a treatment as prevention strategy in a rural South African setting confirmed programmatic challenges in reaching optimum numbers of individuals for HIV testing at home during working hours, especially men, hindering HIV status ascertainment. However, among those contacted, uptake of initial and repeat HIV testing was high. Linkage to care in adults newly diagnosed with HIV was slower than expected, but of those who reached the trial clinic, uptake of ART was high regardless of CD4 count, with good viral suppression and retention. These observations are particularly relevant given the most recent WHO guidelines recommending ART be initiated in anyone diagnosed with HIV, irrespective of CD4 cell count [26]. We show a drop-off at each of the first two steps of the HIV care cascade, which would undermine the effectiveness of such a universal testing and treatment policy in reducing HIV transmission.

We were unable to contact one-quarter of the potential target population, especially men; however, home-based HIV testing was effective in ascertaining the HIV status of those contacted. Our results are in line with those from a meta-analysis including 28 studies that showed a pooled 80% uptake of home-based HIV testing [12]. Individuals unaware of their HIV-positive status cannot benefit from ART for their own health, and as they would remain potential transmitters, the population as a whole would not benefit either. Mobile HIV testing has recently been shown to be more efficient than home-based approaches in increasing contact and testing uptake in men [27–29] and in younger individuals and should be considered as a complementary approach in settings such as ours. Concerns about stigma and breach of confidentiality are often cited as reasons for HIV test refusal, but actual harm is rarely reported in published studies [12]. We identified only one serious adverse event following nearly 11,000 home-based tests, which highlights the quality of the pre- and post-test counselling.

First-time clinic engagement was limited, with only 47.5% of HIV-positive participants not already in care attending a trial or DoH clinic within 6 mo of referral, with 63% linkage at 12 mo. Our findings are in line with the findings from a study in Malawi of HIV self-testing and linkage to care [15] as well as those from a systematic review and meta-analysis of 11 sub-Saharan African studies, which reported that only 57% of those diagnosed HIV-positive had been linked to care [30]. The delay in accessing the trial clinics in our study may be associated with earlier HIV identification, in individuals who were asymptomatic, in home-based testing. Furthermore, our study did not show much difference in delay in accessing care between those in the intervention arm (who were told that ART would be provided to all) and those in the control arm (who were told that ART would be provided to those who were treatment-eligible). However, there were some anecdotal reports that fear of stigma may have discouraged rapid linkage to care, as trial clinics provided services only to HIV-positive individuals; ongoing social science work embedded within the trial may shed more light on this issue [22]. Further, about one-fifth of HIV-positive individuals who entered into care reported being unaware of the link between VL and HIV transmission; this highlights the need to incorporate this information into ART literacy and adherence counselling sessions.

The observed uptake of ART once linked to care was high both in treatment-eligible and not-yet-eligible individuals, with 85% of those initiating ART achieving viral suppression (VL < 400 copies/ml) in both arms. Retention was equally high in the two arms in the first year following treatment initiation. In contrast, in a study in urban Soweto, one in five HIV-positive individuals eligible for ART refused to initiate ART [31]. In that study, “feeling healthy” was the commonest reason given for ART refusal, despite a median CD4 count of 110 cells/μl and high rates of tuberculosis. In a qualitative study in Kenya to explore HIV serodiscordant couples’ attitudes toward early initiation of ART, most participants reported interest in initiating ART early, citing individual health benefits and preventing HIV transmission as motivators [16]; with side effects, lifelong adherence, and stigma emerging as potential barriers.

Overall, we estimated that only one-third of all individuals living with HIV in this population were on ART and virally suppressed. However, we were able to link data from DoH clinics only for the trial participants who were contacted and observed HIV-positive (through DBS results and/or ascertainment). We do not have this information for nearly one-quarter of individuals not reached by the trial or who refused to participate, who may be in HIV care at DoH clinics. Hence, the overall proportion of HIV-positive individuals in the population with viral suppression should be considered an underestimate, while the value of 45.0% (1,343/2,983) with viral suppression computed only among observed HIV-positive individuals constitutes an upper estimate. There remains an important gap in reaching the UNAIDS target of 73% (90% of 90% of 90%) of all people living with HIV being virally suppressed.

The 2013 WHO guidelines for ART recommended initiation at CD4 count ≤ 500 cells/μl and immediate ART initiation among specific groups including serodiscordant couples. Following two large randomised clinical trials [32,33] reporting health benefits in individuals initiating ART at higher CD4 counts, the WHO recently concluded that universal testing and treatment should become the standard of care [26]. Many African countries, including South Africa since January 2015, had already adopted the 2013 guidelines. Trials, including ours, currently underway in South Africa, Zambia, Botswana, Uganda, and Kenya that were originally designed to show a decrease in HIV incidence with ART initiated at a CD4 count of ≤350 cells/μl in the control arm have had to adapt to these expanding treatment eligibility criteria [18,19]. It is likely that when the 2015 WHO HIV treatment guidelines are adopted and implemented in these countries, the research focus in these trials may shift to evaluations of programmes that aim to achieve the WHO/UNAIDS 90-90-90 targets (90% of people living with HIV aware of their HIV status, 90% of people diagnosed HIV-positive on ART, 90% of people on ART virally suppressed) by 2020 [7], which our study shows could potentially be challenging. As these changes have not yet been implemented in South Africa, they do not affect the analysis in this paper.

Study limitations include the use of a subset of the original randomisation for the initial phase of the trial, comprising fewer clusters, but this did not seem to affect the distribution of baseline characteristics between the two arms. Although household contact rates were high, we were unable to contact all individuals identified as eligible for the trial, in particular men. Noncontact could potentially be a source of bias, if different between arms. These limitations highlight the challenges of a universal test and treat policy.

In summary, we show that home-based HIV testing was well received in this population, although men were less easily contactable during the day of home visits, and that immediate ART was acceptable, with good viral suppression and retention. However, only about half of the HIV-positive people identified accessed care within 6 mo, with nearly two-thirds by 12 mo; the improvement with time would suggest that people take time in accessing care, rather than refuse to link to care altogether.

These findings inform the now topical debate of how to identify HIV-positive people in the community and to improve the rate of linkage to care, and provide important input in further statistical projections about the burden of HIV and treatment need for populations with high HIV prevalence, such as in sub-Saharan Africa.

## Supporting Information

S1 Table TasP trial outcomes measured in phase 1 and 2. (DOCX)

S1 Text Trial protocol. (PDF)

S2 Text CONSORT checklist. (DOC)

## Abbreviations

ART: antiretroviral therapy
DBS: dried blood spot
DoH: Department of Health
IQR: interquartile range
UNAIDS: Joint United Nations Programme on HIV/AIDS
VL: viral load
