# Supplementary material for: Uptake of Home-Based HIV Testing, Linkage to Care, and Community Attitudes about ART in Rural KwaZulu-Natal, South Africa: Descriptive Results from the First Phase of the ANRS 12249 TasP Cluster-Randomised Trial
Source: PLoS Med. 2016 Aug 9;13(8):e1002107. doi: 10.1371/journal.pmed.1002107 (PMC4978506; doi:10.1371/journal.pmed.1002107)
Supplement: S1 Table — (DOCX) [file pmed.1002107.s001.docx]

S1 Table. TasP trial outcomes measured in Phase 1 and 2

| **Outcome** | **Indicators** | **Phase 1** | **Phase 2** |
| --- | --- | --- | --- |
| **General population level** | | | |
| HIV incidence | - HIV incidence rate |  | X |
| Acceptability of HIV testing | - Uptake of HIV testing - Attitudes towards HIV testing | X  X | X  X |
| Sexual behaviour and HIV prevention practices | - Sexual partnerships patterns - Marital union dissolution - Uptake of circumcision - Condom use - Contraceptive use - Number of pregnancies | X | X  X  X  X  X  X |
| Quality of life | - Quality of life score |  | X |
| Expenditure  Cost and cost-effectiveness | - Health care use and health care expenditures - Cost analysis - Budget impact |  | X  X  X |
| Community awareness | - Stigma toward people living with HIV/AIDS, perception of stigma - Attitudes towards ART | X  X | X  X |
| **HIV-infected participants** | | | |
| Societal impact | - Gender attitudes and experiences of violence |  | X |
| Acceptability of HIV care | - Attitudes towards HIV care and immediate ART Time to entry into care from referral - Time to ART initiation from first clinic visit | X  X  X | X  X  X |
| Retention | - Retention in care | X | X |
| Morbidity and mortality | - Mortality - Morbidity (HIV and non HIV-related) - Hepatitis B co-infection |  | X  X  X |
| Adherence to ART | - Adherence to ART |  | X |
| HIV drug resistance | - Prevalence and incidence of acquired and transmitted drug resistance |  | X |
| Virological outcomes on ART | - Virological suppression and failure | X | X |
| Toxicity | - Renal dysfunction, liver dysfunction |  | X |
