# Supplementary material for: Uptake of Home-Based HIV Testing, Linkage to Care, and Community Attitudes about ART in Rural KwaZulu-Natal, South Africa: Descriptive Results from the First Phase of the ANRS 12249 TasP Cluster-Randomised Trial
Source: PLoS Med. 2016 Aug 9;13(8):e1002107. doi: 10.1371/journal.pmed.1002107 (PMC4978506; doi:10.1371/journal.pmed.1002107)
Supplement: S1 Text — (PDF) [file pmed.1002107.s002.pdf]

TasP

Antiretroviral Treatment as Prevention • ANRS 12249
Ukuphila kwami, ukuphila kwethu (my health for our health)

A cluster-randomised trial comparing the impact of immediate versus South African recommendations guided ART initiation on HIV incidence The ANRS 12249 TasP (Treatment as Prevention) trial in Hlabisa sub-district, Kwazulu-Natal, South Africa

---

ANRS 12249 TasP Protocol

Version 2.0 • 09/01/2014
Phase 1 (2011-2013) and Phase 2 (2013-2016)

Modification from the Version 1.2 dated March 8th, 2012, signed 14/01/2013. Amendment 1 approved by the Biomedical Research Ethics Committee of the University of KwaZulu-Natal (BREC) on 02/08/2012. Expedited application approved by BREC on 26/09/2012. Amendment 2 approved by BREC on 20/09/2012. Amendment 3 approved by BREC on 14/12/2012. Amendment 4&5 approved by BREC on 15/03/2013. Amendment 6 approved by BREC on 13/08/2013. Amendment 7 approved by BREC on 10/10/2013. Amendment 8 approved by BREC on 12/11/2013. Amendment 9 approved by BREC on 12/03/2014.

Registration number in http://clinicaltrials.gov/: NCT01509508

Registration number in the South African National Trial Register: DOH-27-0512-3974-

Sponsor Inserm - ANRS:

Institut National de la Santé et de la Recherche Médicale (Inserm)
Agence nationale de recherches sur le sida et les hépatites virales (ANRS)
101 rue de Tolbiac
75013 Paris, France
Tel.: +33 1 53 94 60 00 • Fax: +33 1 53 94 60 01

Coordinating Investigators:

Prof. François Dabis
Inserm Unité 897, ISPED
Université Bordeaux Segalen
Bordeaux, France
Tel.: +33 5 57 57 14 36
E-mail: francois.dabis@isped.u-bordeaux2.fr
Prof. Deenan Pillay
Africa Centre for Health and Population Studies
University of KwaZulu-Natal
Somkhele, South Africa
Tel.: +27 35 550 7502 • Fax: +27 35 550 7565
E-mail: dpillay@africacentre.ac.za

Prof. Marie-Louise Newell
Faculty of Medicine, Faculty of Social and Human Sciences
University of Southampton
Southampton, UK
Tel +44 2381 208692
Email: m.newell@soton.ac.uk

TasP

Antiretroviral Treatment as Prevention • ANRS 12249
Ukuphila kwami, ukuphila kwethu (my health for our health)

A cluster-randomised trial comparing the impact of immediate versus South African recommendations guided ART initiation on HIV incidence The ANRS 12249 TasP (Treatment as Prevention) trial in Hlabisa sub-district, Kwazulu-Natal, South Africa

---

ANRS 12249 TasP Protocol

Version 2.0 • 09/01/2014
Phase 1 (2011-2013) and Phase 2 (2013-2016)

Modification from the Version 1.2 dated March 8th, 2012, signed 14/01/2013. Amendment 1 approved by the Biomedical Research Ethics Committee of the University of KwaZulu-Natal (BREC) on 02/08/2012. Expedited application approved by BREC on 26/09/2012. Amendment 2 approved by BREC on 20/09/2012. Amendment 3 approved by BREC on 14/12/2012. Amendment 4&5 approved by BREC on 15/03/2013. Amendment 6 approved by BREC on 13/08/2013. Amendment 7 approved by BREC on 10/10/2013. Amendment 8 approved by BREC on 12/11/2013. Amendment 9 approved by BREC on 12/03/2014.

Registration number in http://clinicaltrials.gov/: NCT01509508

Registration number in the South African National Trial Register: DOH-27-0512-3974-

Sponsor Inserm - ANRS:

Institut National de la Santé et de la Recherche Médicale (Inserm)
Agence nationale de recherches sur le sida et les hépatites virales (ANRS)
101 rue de Tolbiac
75013 Paris, France
Tel.: +33 1 53 94 60 00 • Fax: +33 1 53 94 60 01

Coordinating Investigators:

Prof. François Dabis
Inserm Unité 897, ISPED
Université Bordeaux Segalen
Bordeaux, France
Tel.: +33 5 57 57 14 36
E-mail: francois.dabis@isped.u-bordeaux2.fr
Prof. Deenan Pillay
Africa Centre for Health and Population Studies
University of KwaZulu-Natal
Somkhele, South Africa
Tel.: +27 35 550 7502 • Fax: +27 35 550 7565
E-mail: dpillay@africacentre.ac.za

Prof. Marie-Louise Newell
Faculty of Medicine, Faculty of Social and Human Sciences
University of Southampton
Southampton, UK
Tel +44 2381 208692
Email: m.newell@soton.ac.uk

The ANRS 12249 TasP Trial Team

Steering Committee

Coordinating investigators

François Dabis	Coordinating investigator (France)	Inserm Unité 897, ISPED, Université Bordeaux Segalen, Bordeaux, France<br>francois.dabis@isped.u-bordeaux2.fr
Marie-Louise Newell	Coordinating investigator (United Kingdom)	Faculty of Medicine, Faculty of Social and Human Sciences, University of Southampton, UK<br>m.newell@soton.ac.uk
Deenan Pillay	Coordinating investigator (South Africa)	Africa Centre for Health and Population Studies, University of KwaZulu-Natal, Somkhele, South Africa (SA)<br>d.pillay@africacentre.ac.za

Coordinators

Collins Iwuji	Trial Coordinator/HIV Physician	Africa Centre for Health and Population Studies, SA<br>ciwuji@africacentre.ac.za
Joanna Orne-Gliemann	Trial Coordinator/Social Sciences	Inserm Unité 897, ISPED, France<br>joanna.orne-gliemann@isped.u-bordeaux2.fr

Study team

Till Bärnighausen	Health economics	Africa Centre for Health and Population Studies, SA<br>tbarnighausen@africacentre.ac.za
Sylvie Boyer	Health economics	Inserm Unité 912, Marseille, France<br>sylvie.boyer@inserm.fr
Alexandra Calmy	Adult medicine	Unité VIH/Sida, Hôpitaux Universitaires, Genève, Suisse<br>Alexandra.Calmy@hcuge.ch
Marie-Laure Chaix	Virology	EA 3620, Université paris Descartes,, Paris, France<br>marie-laure.chaix@nck.ap-hop-paris.fr
Tulio de Oliveira	Bioinformatics	Africa Centre for Health and Population Studies, SA<br>tdeoliveira@africacentre.ac.za
Rosemary Dray-Spira	Social sciences	Inserm Unité 1018 CESP, Villejuif, France<br>rosemary.dray-spira@inserm.fr
Ken Freedberg	Modelling	Massachusetts Genera Hospital, Boston, MA, USA<br>kfreedberg@partners.org
John Imrie	Social sciences	Futures Group, SA<br>JImrie@futuresgroup.com
Sophie Karcher	Data management	Inserm Unité 897, ISPED, France<br>sophie.karcher@isped.u-bordeaux2.fr
Joseph Larmarange	Social sciences	CEPED (UMR 196 U. Paris Descartes INED IRD), Paris<br>Africa Centre for Health and Population Studies<br>joseph.larmarange@ceped.org
Richard Lessells	Adult medicine	London School of Tropical Medicine and Hygiene, UK<br>rlessells@africacentre.ac.za
France Lert	Social sciences	Inserm Unité 1018 CESP, Villejuif, France<br>france.lert@inserm.fr
Colin Newell	Data management	Africa Centre for Health and Population Studies, SA<br>cnewell@africacentre.ac.za
Nonhlanhla Okesola	Nurse manager	Africa Centre for Health and Population Studies, SA<br>nokesola@africacentre.ac.za
Bruno Spire	Social sciences	Inserm Unité 912, Marseille, France<br>bruno.spire@inserm.fr
Frank Tanser	Epidemiology and Biostatistics	Africa Centre for Health and Population Studies, SA<br>ftanser@africacentre.ac.za
Rodolphe Thiébaut	Epidemiology and Biostatistics	Inserm Unité 897, ISPED, France<br>rodolphe.thiebaut@isped.u-bordeaux2.fr
Johannes Viljoen	Virology Lab Head	Africa Centre for Health and Population Studies, SA<br>jviljoen@africacentre.ac.za
Thembelihle Zuma	Psychology	Africa Centre for Health and Population Studies, SA<br>tzuma@africacentre.ac.za

Sponsor representatives

	Brigitte Bazin	Head of ANRS "Research in Developing Countries" Unit<br>brigitte.bazin@anrs.fr
	Claire Rekacewicz	ANRS Trial Correspondent<br>claire.rekacewicz@anrs.fr
	Alpha Diallo	ANRS Pharmacovigilance unit<br>alpha.diallo@anrs.fr

Scientific Advisory Board

**Chair**	Pr. Bernard Hirschel	Unité VIH/Sida, Hôpitaux Universitaires, Genève, Suisse<br>bernard.hirschel@hcuge.ch
**Members**	Xavier Anglaret, Hoosen Coovadia, , Bruno Giraudeau, Jean-Michel Molina, Lynn Morris,, Francois Venter, Sibongile Zungu. Non-voting members: Eric Fleutelot, Eric Goemaere, Calice Talom Merck and Gilead representatives, 3ie representatives.	

Data Safety and Monitoring Board

**Chair**	Pr. Patrick Yeni	Department of Infectious Diseases, Hôpital Bichat-Claude Bernard, Paris, France<br>patrick.yeni@bch.aphp.fr
**Members**	Nathan Ford, Hakima Himmich, Catherine Hankins, Helen Weiss, Sinead Delany-Moretlwe.	

Sponsor

Institut National de la Santé et de la Recherche Médicale - Inserm
Agence Nationale de Recherches sur le Sida et les hépatites virales - ANRS
101 rue de Tolbiac, 75013 Paris, France
Director: Prof. Jean-François Delfraissy

Atripla® to be used in the trial will be provided by Merck & Co. and Gilead

Abstract

Title: A cluster-randomised trial comparing the impact of immediate versus South African recommendations guided ART initiation on HIV incidence. The ANRS 12249 TasP (Treatment as Prevention) trial in Hlabisa sub-district, Kwazulu-Natal, South Africa

Background: Thirty years after the discovery of the human immunodeficiency virus (HIV), prevention is difficult to achieve and the pandemic does not show any sign of abating. Antiretroviral therapy (ART) is now rolled out at a large scale in lower-income countries. ART with fully suppressive antiretroviral (ARV) drugs combinations lowers HIV viral load (VL) in all body compartments and decreases the risk of transmission to a low level. It is thus legitimate to raise the following question: Could ART contribute to reducing transmission at individual and population level? Not only may earlier treatment reduce HIV incidence (acquisition of new cases of HIV infection through sexual or mother-to-child transmission), it may also benefit the individual. The long-term benefits of starting ART earlier would likely be of particular importance in settings where the incidence of life-threatening HIV-related diseases occurring at relatively high CD4 levels (tuberculosis, invasive bacterial diseases, and possibly malaria) is substantial, a typical situation in most sub-Saharan Africa including South Africa.

Research hypothesis: HIV testing of all adult members of a community, followed by immediate ART initiation of all, or nearly all, HIV-infected participants regardless of immunological or clinical staging will prevent onward transmission and reduce HIV incidence in this population.

Objectives: To estimate the effect of ART initiated immediately after HIV diagnosis, irrespective of CD4 count criteria, on the reduction in incidence of new HIV infections in the general population in the same setting.

Setting: The trial will be conducted in Hlabisa sub-district, Umkhanyakude district, Northern KwaZulu-Natal, South Africa. This rural setting of 1430 km^2 in size has a population of approximately 220 000 Zulu-speaking people. In this sub-district, the Africa Centre for Health and Population studies, a research institute at the University of KwaZulu-Natal (http://www.africacentre.com) carries out socio-demographic and HIV surveillance and clinical research. The KwaZulu-Natal Department of Health and the Africa Centre established in 2004 the Hlabisa HIV Treatment and Care Programme, devolved to all 17 primary health care clinics in the sub-district. By mid-2013, over 28 000 HIV-infected people eligible for treatment had been initiated on ART; patients' treatment eligibility is determined by South African guidelines.

Design: A cluster-randomised trial with 22 (2×11) clusters will be conducted within the Hlabisa sub-district, covering a total population of approximately 22 000 inhabitants aged 16 years and above, of whom an estimated 17 600 will be HIV-negative. A full prevention and HIV testing strategy will be provided in both the intervention and control arms, consisting of the current range of community and clinic testing options plus the implementation of 6-monthly rounds of home-based HIV testing. The adult HIV-infected population residing in the intervention clusters will be offered immediate ART initiation upon HIV diagnosis whereas the HIV-infected population in the control clusters will be offered ART according to national guidelines (CD4 less than 350 cells/ml, WHO stage 3 or 4 disease or MDR/XDR TB). The protocol outlines the overall trial design, which has HIV incidence as primary outcome. The first phase of the trial (24 months) will take place in ten (2×5) clusters, with three rounds of home-based HIV testing and surveillance according to the trial protocol, and has as main outcome

acceptability and feasibility rather than HIV incidence. If results from the first phase indicate acceptability and feasibility, the trial will be rolled-out to the other 12 clusters during the second phase. Possible amendments to the trial for phase 2 will be based on the advice from the Data Safety Monitoring Board and the Scientific Advisory Board.

Trial eligibility criteria: To be aged 16 years and above and a member of a household in the designated cluster (head of household defines membership status in KwaZulu culture).

Treatment eligibility criteria: Those already on ART from the Hlabisa HIV Treatment and Care Programme may opt to transfer their care to the trial; the few (if any) people already on ART from private/other HIV treatment providers will be encouraged to take part in the trial monitoring procedures, and be given the opportunity to change ART provider to the Hlabisa HIV Treatment and Care Programme trial.

Trial treatment: The standard first-line drug regimen will be the combination of tenofovir (TDF) + emtricitabine (FTC) + efavirenz (EFV) once daily, i.e. Atripla®. This will also be applicable to pregnant women irrespective of the trimester of pregnancy.

Follow-up: The treatment and follow up of HIV-infected patients in the first phase will be for a maximum of 24 months (March 2012-March 2014) and this will continue for another 24 months with the additional 12 clusters from 2014.

Data collection: Quantitative data will be collected from participants during each round of HIV counselling and testing in the community to build a comprehensive individual, household and, ultimately, population picture of key social, demographic, behavioural, partnership and economic issues (Individual home-based questionnaire). Clinical, biological and social data will be collected from HIV-infected participants on ART or not attending the trial clinics during routine follow-up appointments (Clinic-based survey). In addition, activity measures will be collected from each of the trial clinics (Structure questionnaire).

Trial primary outcome: HIV incidence, measured at the end of the trial, that is 48 months in the 10 phase 1 clusters and 24 months in the 12 additional clusters in phase 2, using two approaches: 1) using dried blood spots (DBS), with longitudinal follow-up and 2) using a locally validated test for recent infection: capture BED enzyme-linked immunoassay (cBED assay). The latter will be used to validate recent infections in material collected in the first round of testing to confirm assumptions used in sample size calculations.

Trial secondary outcomes Behavioural and socio-economic outcomes (such as acceptability of HIV counselling and testing, sexual partnerships, quality of life, household expenditures, cost-effectiveness) and clinical outcomes (programme retention, mortality, incidence of severe morbidity including tuberculosis, adherence to ART measured by self-report and virological response on treatment, new cases of vertically-acquired HIV infections and MTCT rates in the area, acquired HIV drug resistance) measured after 24 months of follow-up.

First phase outcomes: The first phase will specifically focus on the trial secondary outcomes in order to: 1) assess the acceptability and feasibility of the intervention; and 2) validate and update the parameters of the model used to estimate the trial sample size and HIV incidence, in terms of: uptake of HIV testing, linkage to care upon HIV diagnosis, internal migration and ART initiation.

Timing: The entire trial, including planning, will take place over six years (2011-2016). The first phase will take 24 months. Data analysis and review of results

will be performed during 2013 to inform decision-making regarding process and procedures for phase 2.

Sample size: A fully parameterised, deterministic mathematical model demonstrates that a 34% reduction in cumulative HIV incidence (4.5% versus 2.98%) in HIV-negative participants over two years should be feasible across a wide range of parameter space. Sample size calculations indicate that 22 clusters (11 in each arm), with 1 000 consenting participants >15 years of age in each cluster (N=22 000; 17 600 HIV-negative), are required to achieve this objective. The first phase will be conducted on 10 000 participants in 10 clusters, which allows the measurement of the proportion agreeing to test over three rounds of testing within 1% (95% CI) and uptake of testing in the intervention communities of all HIV-positive participants within 4% (95% CI).

Expected results: We aim to provide proof-of-principle evidence regarding the effectiveness of Treatment-as-Prevention in reducing HIV incidence at the population level. We will collect data from the participants to inform the generalizability of the results, and thus inform policy resulting in wide implementation.

Résumé

Titre : Un essai randomisé en cluster comparant l'impact d'une mise sous traitement ARV immédiate versus les recommandations sud-africaines sur l'incidence du VIH. L'essai ANRS 12249 TASP (traitement par la prévention) dans le sous-district de Hlabisa, Kwazulu-Natal, Afrique du Sud.

Contexte : Trente ans après la découverte du virus de l'immunodéficience humaine (VIH), la question de la prévention du VIH est encore non résolue. Les traitements antirétroviraux (ARV) sont désormais mis en place à large échelle dans les pays à ressources limitées. Or il a été montré qu'un traitement ARV combinant des molécules ayant une forte capacité de suppression virale permettait de réduire la charge virale (CV) dans tous les compartiments corporels et de réduire le risque de transmission du VIH à de très faibles niveaux. Il semble donc légitime de se poser la question suivante : les traitements ARV pourraient-ils contribuer à réduire la transmission du VIH aux niveaux individuels et populationnels ? Non seulement le traitement précoce pourrait réduire l'incidence du VIH (les nouveaux cas d'infection par transmission sexuelle et transmission mère-enfant), mais il pourrait également offrir des bénéfices individuels. Les bénéfices à long terme du traitement précoce seraient d'autant plus grands que l'incidence des maladies opportunistes graves liées au VIH (tuberculose, infections bactériennes invasives et probablement le paludisme) survenant à des taux élevés de CD4 est élevée, comme c'est le cas dans la plus grande partie de l'Afrique sub-saharienne et notamment l'Afrique du Sud.

Hypothèses de recherche : Le dépistage VIH de tous les membres d'une communauté, suivi de la mise sous traitement immédiat de tous, ou quasiment tous, les individus infectés par le VIH, quel que soit leur statut immunologique ou clinique, préviendrait la transmission du VIH et réduirait l'incidence du VIH dans cette population.

Objectifs : Estimer directement l'impact du traitement ARV initié immédiatement après le diagnostic de l'infection par le VIH et quel que soit le niveau de CD4 des patients non encore éligibles au traitement ARV, sur l'incidence de nouvelles infections VIH dans la population générale de la même région.

Environnement : L'essai sera conduit dans le sous-district de Hlabisa, district de Umkhanyakude dans la province du KwaZulu Natal en Afrique du Sud. Cette zone rurale de 1 430km² compte approximativement 220 000 habitants, l'incidence du VIH y est estimée à 3,4%. L'Africa Centre for Health and Population Studies, un institut de recherche de l'Université du KwaZulu Natal (http://www.africacentre.com), y conduit des activités de surveillance démographique et des questions de santé dont l'infection à VIH/Sida. Le Département pour la Santé de la province du KwaZulu Natal et l'Africa Centre ont lancé en 2004 le Programme de Traitement et de Prise en Charge de Hlabisa, décentralisé à l'ensemble des 17 centre de santé primaires du sous-district. Mi-2013, plus de 28 000 individus infectés par le VIH et éligibles au traitement ARV, selon les recommandations actuellement en vigueur en Afrique du Sud, en bénéficiaient.

Méthodologie : Un essai randomisé en grappes (« clusters ») sera conduit dans le sous-district de Hlabisa au sein de 22 (2x11) grappes comprenant un total de 22 000 individus âgés de plus de 15 ans, 17 600 étant séronégatifs au début du programme. Un paquet global et le plus complet possible de services de prévention et de dépistage du VIH sera mis en place dans les deux groupes de grappes. Il s'agira notamment de combiner les services existants de dépistage à la

clinique et au sein de la communauté, et la mise en place de cycles de six puis de quatre mois permettant d'offrir le dépistage du VIH à domicile. La population adulte infectée par le VIH et résidant dans les grappes tirées au sort pour constituer le bras « intervention » pourra être mise sous traitement ARV immédiatement tandis que la mise sous traitement de la population des grappes constituant le groupe de comparaison se fera selon les procédures actuelles recommandées par le gouvernement sud-africain, en incluant les individus présentant avec un taux de lymphocytes CD4 <350 cellules/ml. L'essai sera conduit en deux phases. Le présent protocole décrit le schéma d'étude dans son ensemble, étude dont le critère de jugement principal sera l'incidence du VIH. La première phase de l'étude (24 mois) sera conduite dans 10 (2×5) grappes : trois cycles de dépistage VIH à domicile seront conduits, toutes les procédures du protocole seront mises en œuvre et les critères de jugement principaux seront l'acceptabilité et la faisabilité de l'intervention et non pas l'incidence du VIH. Si les résultats de la première phase montrent l'acceptabilité et la faisabilité de l'intervention, l'essai sera mis en place dans l'ensemble des 12 autres grappes en phase 2. Des amendements possibles au protocole de l'essai pourront être envisagés suivant les recommandations du Comité Indépendant de Surveillance et du Conseil Scientifique.

Critères d'éligibilité pour l'essai : Être âgé de 16 ans ou plus et être un membre d'un ménage de la grappe sélectionnée (dans la culture KwaZulu, le chef de ménage identifie les membres du ménage)

Critères d'éligibilité pour le traitement TasP : Les personnes déjà sous traitement ARV au sein du Programme de Traitement et Prise en Charge de Hlabisa seront recrutés dans l'étude ; le faible nombre de ceux déjà sous traitement ARV et suivis dans le secteur privé ou par d'autres professionnels de santé seront encouragés à participer à l'essai et pourront intégrer le Programme de Traitement et Prise en Charge de Hlabisa s'ils le désirent.

Intervention ARV de l'essai : Le régime ARV de première ligne combinera le tenofovir (TDF) + l'emtricitabine (FTC) + l'efavirenz (EFV) en une prise par jour. Ce régime s'appliquera aussi aux femmes enceintes, quelque soit leur âge gestationnel.

Suivi : La durée de traitement et de suivi pour les patients infectés par le VIH est d'un maximum de 24 mois durant la première phase (Mars 2012-Mars 2014) et continuera pendant 24 supplémentaires dans les 12 clusters additionnels de la phase 2.

Collecte des données : Des questionnaires seront administrés à chaque cycle de dépistage à domicile, afin de décrire au niveau individuel, du ménage et de la communauté les données sociales, démographiques, comportementales et économiques des participants et les paramètres cliniques et biologiques des patients sous traitement ARV. Des données quantitatives seront également collectées auprès des patients suivis au sein des cliniques de l'essai au cours de leurs visites de routine. Enfin, des donnés d'activité et de processus seront collectées au sein de chaque clinique de l'essai.

Critère de jugement principal de l'essai : L'incidence du VIH, mesurée à la fin de l'essai (48 mois dans les clusters de phase 1 et 24 mois dans les 12 clusters additionnels de la phase 2), 1) en utilisant les échantillons collectés sur papier buvard au cours du suivi à long terme et 2) en utilisant un test validé en Afrique du Sud pour estimer l'infection récente, le capture BED enzyme-linked immunoassay (cBED assay)

Critères de jugement secondaires de l'essai : Des indicateurs comportementaux et socio-économiques tels que l'acceptabilité du conseil et du

dépistage du VIH, les partenaires sexuels, la qualité de vie, les dépenses des ménages, le rapport coût-efficacité et des indicateurs cliniques (rétention dans l'essai, la mortalité, l'incidence d'évènements morbides sévères dont la tuberculose, l'adhérence au traitement ARV, les cas d'infections pédiatriques et les cas de résistance virale acquise).

Critères de jugement de la première phase : La première phase de l'essai répondre en particulier aux objectifs secondaires de l'essai pour 1) évaluer l'acceptabilité et la faisabilité de l'intervention et 2) valider et mettre à jour les paramètres du modèle utilisé pour estimer la taille de l'échantillon et l'incidence du VIH, en termes de: dépistage du VIH, accès aux soins de liaison après diagnostic du VIH, les migrations internes et les critères d'initiation ART.

Calendrier prévisionnel : L'essai, y compris la période de mise en place, est prévu sur six ans (2011-2016). La première phase est prévue sur 24 mois. L'analyse des données réalisée en 2013 permettra de se prononcer sur la suite de l'essai et/ou la modification de certaines procédures en phase 2.

Taille de l'échantillon : Un modèle mathématique déterministe et entièrement paramétré a montré la faisabilité sur deux ans d'une réduction de 34% de l'incidence cumulée du VIH (4,5% vs. 2.98%) au sein de participants séronégatifs. Les calculs de taille d'échantillon suggèrent que 22 grappes (11 dans chaque bras) comprenant 1 000 participants de plus de 15 ans dans chaque grappe (N=22 000, 17 600 personnes séronégatives au début de l'essai) sont nécessaires pour répondre à cet objectif. La première phase portera sur 10 000 personnes dans 10 grappes, ce qui permet de documenter à 1% près la proportion de personnes acceptant le test au cours des trois cycles de dépistage (IC 95%) et à 4% près la couverture du dépistage dans les communautés des grappes du bras « intervention » de tous les individus infectés par le VIH

Résultats attendus : Nous espérons montrer que la stratégie du Traitement comme moyen de Prévention de la transmission du VIH peut être mise en place au sein d'une population à forte incidence du VIH, quel que soit le stade de l'infection et les caractéristiques du dépistage des individus de cette population, et que cette approche novatrice contribue à réduire l'incidence du VIH et offre des bénéfices individuels et populationnels. Les résultats documentés dans cet essai devraient être suffisamment forts pour encourager la réplication de cette stratégie dans d'autres pays à ressources limitées et avoir des implications politiques permettant sa mise en place à large échelle.

List of abbreviations and definitions

3TC	Lamivudine
AE	Adverse Event
ANRS	Agence Nationale de Recherches sur le SIDA et les hépatites virales
ART	AntiRetroviral Treatment
ARV	AntiRetroViral (drug)
bd	Twice daily
BIA	Budget Impact Analysis
BMI	Body Mass Index
CAB	Community Advisory Board
CAR	Clinic Activity Report
CAP	Consumer Advisory Panel
CBC	Clinic Baseline Visit (Counsellor) questionnaire
CEA	Cost-Effectiveness Evaluation
CFU	Clinic Follow-Up (Counsellor) questionnaire
CHE	Clinic History and Examination questionnaire
CRF	Case Report Form
d4T	Stavudine
DBS	Dried Blood Spot
DoH	Department of Health
DSA	Demographic Surveillance Area
DSMB	Data Safety Monitoring Board
EFV	Efavirenz
FDC	Fixed-Dose Combination
FTC	Emtricitabine
GCP	Good Clinical Practices
GIS	Geographical Information System

HAART	Highly Active AntiRetroviral Therapy
HASI-P	HIV/AIDS stigma instrument for people living with HIV/AIDS
HAT-QOL	HIV/AIDS-Targeted Quality of Life (scale)
HBsAg	Hepatitis B surface antigen
HBV	Hepatitis B Virus
HHI	TasP Household Information Assets questionnaire
HIV	Human Immunodeficiency Virus
HSV2	Herpes Simplex Virus
ICH	International Conference on Harmonisation
IEC	Information, Education, Communication
IQ	TasP Home-based Individual Questionnaires
IT	Information Technology
LPV/r	Lopinavir/ritonavir (boosted)
MCC	Medicines Control Council of South Africa
MDR TB	Multi-Drug Resistant Tuberculosis
NGO	Non-Governmental Organisation
Nocte	Every night
NVP	Nevirapine
od	Once daily
PEP	Post-Exposure Prophylaxis
PIT	Pill Identification Test
PITC	Provider-Initiated Testing and Counselling
PLWHA	Person Living With HIV/AIDS
PMTCT	Prevention of Mother-To-Child Transmission of HIV
ppy	person per year
PROQOL	Patient Reported Outcomes Quality Of Life
QALY	Quality-Adjusted Life Years
RCT	Randomised Clinical Trial

SAB	Scientific Advisory Board
SAE	Serious Adverse Event
SC	Steering Committee
SCB	Social Science Clinic-based Baseline questionnaire
SCC	Social Science Clinic-based Counsellor-administered questionnaire
SCI	Social Science Clinic-based Interviewer-administered questionnaire
SCS	Social Science Clinic-based Survey (SCB + SCC + SCI)
SOP	Standard Operating Procedure
STI	Sexually Transmitted Infection
TB	Tuberculosis
TDF	Tenofovir disoproxyl fumarate
THR	TasP Household registration questionnaire
VAS	Visual Analogue Scale
VCT	Voluntary Counselling and Testing (HIV)
VL	Viral Load (HIV plasma RNA measurement)
WHO	World Health Organization (Switzerland)
XDR TB	Extensively Drug-Resistant Tuberculosis
ZDV	Zidovudine

Table of contents

The ANRS 12249 TasP Trial Team 3
Abstract 5
Résumé 8
List of abbreviations and definitions 11
1. Rationale 21
1.1 State of knowledge 21
1.1.1 The challenges of prevention of sexual transmission of HIV 21
1.1.2 Antiretroviral therapy can reduce sexual transmission 21
1.1.3 Can ART be provided at earlier stages of HIV infection than currently recommended? 21
1.1.4 Strengthening HIV testing approaches 22
1.1.5 Could combination antiretroviral therapy be used universally to reduce sexual transmission of HIV at population level? If yes, which combination? 23
1.2 Study hypothesis 24
1.3 Study design 24
1.4 Trial phases 24
1.5 Expected results of the trial 24
2. Trial setting 25
2.1 Hlabisa sub-district 25
2.2 The South Africa HIV treatment programme 26
2.3 Hlabisa HIV Treatment and Care Programme 26
2.4 The Africa Centre for Health and Population Studies 27
3. Trial objectives 28
3.1 Overall objectives of the trial (first and second phase) 28
3.1.1 Main objective 28
3.1.2 Specific objectives 28
Among all participants 28
Among HIV-infected participants only 28
Within the health system 28
3.2 Objectives specific to the first phase 29
3.2.1 Main objective 29
3.2.2 Specific objectives 29
Among all participants 29
Among HIV-infected participants 29
Within the health system 29

Other objectives 29
4. Overall trial plan 30
4.1 Description of the two trial phases 30
4.1.1 First phase 30
4.1.2 Second phase 30
4.2 Trial timelines 30
4.2.1 First phase (2011-2013) 32
4.2.2 Second phase (2013-2016) 32
4.3 Description of the trial participants 32
5. Trial components 33
5.1 Component 1: HIV counselling and testing and comprehensive prevention programme 33
5.2 Component 2: ART for HIV infected participants 34
6. Trial conduct 35
6.1 Preparing the community 35
6.2 Baseline assessment, enrolment of participants, HIV counselling and testing 35
6.2.1 Approaching households, obtaining individual consent, and administrating population-based questionnaires 35
6.2.2 HIV counselling and testing procedures 36
6.2.3 Referral and linkage to care procedures 37
6.3 Preventive interventions 38
6.3.1 Information and education, condom promotion and distribution 38
6.3.2 Male circumcision 38
6.3.3 Syndromic management of sexually transmitted infections 39
6.3.4 Post-exposure prophylaxis after sexual assault 39
6.3.5 Family planning 39
6.4 Management and care of HIV-infected participants 40
6.4.1 Pre-treatment assessment visit (W0) 40
6.4.2 ART initiation criteria 41
6.4.3 ART drugs used within the trial 41
6.4.3.1 Description of the drugs 41
6.4.3.2 Supply 44
6.4.3.3 Drug handling and storage 44
6.4.4 Patient follow-up 44
6.4.4.1 ART initiation visit 44
6.4.4.2 Treatment duration 44
6.4.4.3 ART follow-up visits 47

6.4.5 Trial treatment modifications 50
6.4.5.1 Occurrence of new pregnancies 50
6.4.5.2 Toxicity and treatment failure 50
6.4.5.3 Care of patients at the end of the trial 51
6.4.5.4 Concomitant therapies 51
6.4.6 Sample handling and storage 51
7. Trial outcomes and tools 52
7.1 Definition of trial outcomes 52
7.2 Primary outcome 54
7.2.1 HIV status measurement, using DBS with longitudinal follow-up 54
7.2.2 A locally validated test for recent infection: capture BED enzyme-linked immunoassay (cBED assay) 55
7.3 Secondary outcomes 55
7.3.1 Both within the general population level, stratified by HIV status, and among HIV-infected participants 55
7.3.1.1 Acceptability and uptake of HIV testing 55
7.3.1.2 Behavioural changes at individual level and sexual partnerships ... 56
7.3.1.3 Quality of life 56
7.3.1.4 Cost-effectiveness performance 57
7.3.1.5 Community awareness, attitudes and behaviours 58
7.3.1.6 Societal response 58
7.3.2 Only among HIV-infected participants 58
7.3.2.1 Acceptability and uptake of entry in care and treatment 58
7.3.2.2 Therapeutic success/evaluation of ART 59
7.3.2.3 Programme retention 59
7.3.2.4 Mortality, severe morbidity, and tuberculosis 59
7.3.2.5 Adherence 59
7.3.2.6 Paediatric HIV infection 59
7.3.2.7 HIV drug resistance 60
7.3.2.8 Molecular epidemiology 61
7.4 Data collection tools 61
7.4.1 Household and individual home-based questionnaires 61
7.4.2 Social science Clinic-based survey 62
7.4.3 Case Report Forms (CRF) 63
7.4.4 Clinic Activity Reports (CAR) 63
7.4.5 Qualitative data 63
7.5 Sub-studies 64

7.5.1 Survey of health care professionals 64

7.5.2 Budget impact analysis 64

8. Methodological and statistical considerations 65

8.1 Cluster selection 65

8.2 Modelling exercise and sample size calculation 66

8.2.1 Principles of the model 66

8.2.2 Model input parameters 66

8.2.3 Sample size calculation based on model assumptions 68

8.3 Randomization 70

8.4 Plan of analysis 71

8.4.1 Criteria for continuation/discontinuation at the end of the first phase 71

8.4.1.1 Feasibility 71

8.4.1.2 Acceptability 71

8.4.2 Statistical analysis 72

8.4.3 Safety 73

8.5 Adverse events: definitions and reporting 73

8.6 Serious adverse events: definition and reporting 73

8.6.1 Definition 73

8.6.2 Assessment of causality 74

8.6.3 SAE reporting 74

8.6.3.1 Initial notification 74

8.6.3.2 Complementary notification 75

8.6.3.3 Reporting to Ethics Committee / DSMB / KZN Department of Health / Steering Committee 75

8.7 Pregnancy reporting 75

8.8 Annual safety report 76

9. Trial oversight 77

9.1 Steering Committee (SC) and Coordination Team (CT) 77

9.2 Data Safety Monitoring Board (DSMB) 78

9.3 Scientific Advisory Board (SAB) 78

10. Ethic and regulatory aspects 80

10.1 Ethics and competent authorities 80

10.1.1 Africa Centre Community Advisory Board (CAB) 80

10.1.2 Biomedical Research Ethics Committee of the University of KwaZulu-Natal 80

10.1.3 KwaZulu-Natal Department of Health (DoH) 81

10.2 Community, participant and patient information and consent 81

10.3 Data confidentiality 82
10.4 Protocol amendments 83
10.5 Investigators responsibilities 83
10.6 Insurance 83
11. Data management and monitoring 84
11.1 Data collection and storage of forms 84
11.2 Data management 84
11.3 Exchange of data 85
11.4 Quality assurance, monitoring, audits and inspections 85
11.4.1 Monitoring 85
11.4.2 Audits and inspections 86
12. Archiving 87
13. Results publications 88
14. References 89
15. Appendices 95
15.1 Appendix 1. National Department of Health Antiretroviral Treatment guidelines (March 2013) 96
15.2 Appendix 2. Reviewing the available evidence for the choice of trial medications 118
15.3 Appendix 3. Support letter from Department of Health 121
15.4 Appendix 4. Information and consent forms 124
15.5 Appendix 5. Follow-up algorithms 170
15.5.1 Intervention clusters 170
15.5.2 Control clusters 171
15.6 Appendix 6. Insurance certificate 172
15.7 Appendix 7. UKZN Biomedical Research Ethics Committee approval letter 174

Tables List

Table 1 Antiretroviral regimens proposed to TasP patients 43

Table 2 Follow-up calendar for HIV-infected participants eligible for ART 45

Table 3 Topics of Social science clinic-based survey, by questionnaire 48

Table 4 Follow-up calendar of HIV-infected participants not eligible for ART 49

Table 5 Definition of trial outcomes 52

Table 6 Outcomes documented within sub-studies 54

Table 7 Data collection during the HIV testing rounds, among the general population: Household and individual questionnaires (first phase of the trial) 62

Table 8 Parameter settings and characteristics of HIV epidemic as modelled by the STDSIM model 67

Table 9 Projected 5-year impact of cluster-randomized trial of treatment as prevention on HIV incidence and ART coverage in rural KwaZulu-Natal, South Africa. 67

Table 10 Estimated power to show a 34% reduction in cumulative incidence using the Satterthwaite approximate F test for two-sample comparison of proportions with clustering for 22 clusters (2012-2015). 69

Table 11 Participants at risk of sero-conversion and expected number of incident cases 70

Figures List

Figure 1 Hlabisa sub-district, uMkhanyakude district, Northern KwaZulu-Natal, South Africa (shaded area is the area of the Africa Centre surveillance) 25

Figure 2 Timeline for the overall TasP trial 31

Figure 3 Description of the different components of the TasP trial..........................33

Figure 4 Home visit chart. 37

Figure 5 Projected 5-year impact of cluster-randomized trial of treatment as prevention on HIV incidence rates in rural KwaZulu-Natal, South Africa. 68

Figure 6 Trial oversight..77

1. Rationale

1.1 State of knowledge

1.1.1 The challenges of prevention of sexual transmission of HIV

Twenty-five years after the discovery of the human immunodeficiency virus (HIV), successful prevention remains challenging and the pandemic continues unabated (1). A vaccine remains elusive despite recent advances in the past two years (1, 2). Vaginal microbicides without antiretroviral (ARV) drugs have failed to show significant long-term benefit (3); the recent results from the Tenofovir (TDF) - containing microbicide are promising but need confirmation in a further trial (4). Treatment of sexually transmitted infections, including herpes simplex has no measurable effect on HIV transmission (5). Male circumcision halves the risk of HIV transmission from females to males but is not (yet) a widely-used public health measure, although the South African government has started implementing a programme, focussing on young men in April 2010. As a consequence, effective prevention continues to rely on behavioural change and condom use, but these methods have their limitations, as evidenced by the resurgence of unsafe sex in homosexual males in Western Europe and the USA (6), and limited uptake and more importantly sustainability in lower-income countries (7, 8). At their current or anticipated level of use none of these methods is effective enough to contain the pandemic in countries with high incidence and prevalence of HIV infection such as South Africa.

1.1.2 Antiretroviral therapy can reduce sexual transmission

HIV plasma viral load (VL) in the index HIV-infected individual is the dominant determinant of transmission, documented in heterosexual couples and mother/child pairs (9, 10). Antiretroviral therapy (ART) with fully suppressive ARV drugs combinations lowers VL in all body compartments and decreases the risk of transmission to a very low level. It is thus legitimate to raise the following question: **Could ART contribute to reducing transmission at individual and population levels?** Head-to-head comparisons are not available and are unlikely to be promoted, but in cohort studies where routine use of condoms was encouraged in sero-discordant couples, results differed substantially depending on whether index patients were on ART (0.46 case of HIV acquisition per 100 person-years [ppy]) or not (5.64 ppy) (11). In the same meta-analysis, Attia et al estimated a zero (97.5% upper confidence limit of 1.27 ppy) transmission risk for those on ART and with successful viral suppression (VL <400 copies/ml). The HPTN 052 study showed that ART reduces HIV transmission by 96% in stable couples where one partner is HIV-infected and the other is not (12). This NIH trial has not addressed the question of the reduction of HIV transmission at the population level.

1.1.3 Can ART be provided at earlier stages of HIV infection than currently recommended?

Until 2009 international recommendations for initiating ART were generally conservative everywhere in the world, especially in resource-limited settings where ART initiation was recommended only at a CD4+ (CD4) count of 200 cells/mm^3 or less or for end-stage HIV disease. However, recently two pieces of evidence considerably changed this approach: analyses of observational data from the USA and Europe (13, 14) and a randomized clinical trial conducted in

Haiti (15). Further, there is some evidence of benefit of earlier treatment from trials of structured treatment interruption. For instance, the ANRS 1269 TRIVACAN trial conducted in Côte d'Ivoire showed that patients interrupting ART at intermediate CD4 levels had higher risks of morbidity and mortality than patients remaining on ART (16). Finally, observational cohort data from Zimbabwe have shown that the risk of death of untreated HIV-infected women was 6.2 times higher for those with more than 600 CD4 when compared with HIV-negative women (17).

In November 2009, WHO recommended to substantially broaden eligibility for ART, with treatment of all HIV infected people with CD4 <350 cells/mm^3 irrespective of clinical symptoms or at WHO clinical stage 3 or 4 irrespective of CD4 count. Treatment should also be provided for those with a diagnosis of active tuberculosis (TB) irrespective of CD4 cell count, those who have a co-infection with hepatitis B virus (HBV) if the latter requires treatment and for pregnant women fulfilling treatment criteria (18). Two clinical trials are currently investigating whether ART initiated well above 350 cells/mm^3 provides sufficient additional individual benefits in terms of mortality and severe morbidity: the NIH-sponsored START trial (clinicaltrials.gov identifier NCT00867048) and the ANRS-sponsored TEMPRANO trial (clinicaltrials.gov identifier NCT00495651). Although the results of these trials are not expected before the end of 2015, evidence and programmatic experience have continued to shift the risk-benefit ratio towards starting ART earlier. Thus in June 2013, the revised WHO guidelines promoted "expanded eligibility for ART with a CD4 threshold for treatment initiation of 500 cells/mm^3 or less for adults, adolescents and older children. Priority should be given to individuals with severe or advanced HIV disease and those with CD4 count of 350 cells/mm^3 or less. ART is recommended to be initiated regardless of CD4 count for certain populations, including people with active tuberculosis (TB) disease who are living with HIV, people with both HIV and hepatitis B virus (HBV) infection with severe chronic liver disease, HIV-positive partners in serodiscordant couples, pregnant and breastfeeding women and children younger than five years of age" (19).

1.1.4 Strengthening HIV testing approaches

Without HIV testing, bio-medical interventions cannot target HIV-infected individuals. Motivations for testing vary, but many people test to obtain treatment, often when they feel unwell. Restricting treatment to those who have already developed symptoms and meet CD4 count criteria as discussed in the previous section may actually serve as a serious, if unintentional, barrier to the uptake of HIV testing.

Over the past two decades, HIV counselling and testing services have primarily been promoted and provided in the context of Voluntary HIV Counselling and Testing (VCT). However, the limits of VCT approaches in resource-limited settings, particularly regarding achieving early diagnosis, high levels of population coverage and access to "hard-to-reach" groups (20, 21), have led to the development of other innovative forms of HIV testing such as Provider-Initiated Testing and Counselling (HIV testing and counselling recommended by health care providers for any persons attending health care facilities as a standard component of medical care, such as HIV testing in TB clinics, door-to-door testing, partner notification), home-based or mobile HIV testing that take testing services to people (22).

Defining a feasible, acceptable, and efficient strategy to obtain very high rates of testing (e.g. near universal HIV testing repeated bi-annually) may necessitate further development of both existing VCT services available and extension of

PITC. The South African government strongly supports a coherent, consistent HIV services' approach, encouraging all public health facilities – fixed and mobile – to offer HIV testing. Successfully testing the numbers needed to achieve the coverage required for implementation of treatment for all or nearly all infected people is quite different to simply promoting VCT, and will certainly necessitate a more intensive approach to provider-initiated services, taking into account the needs and attitudes of different population groups and considering the relevance of different modes of HIV testing in different populations.

1.1.5 Could combination antiretroviral therapy be used universally to reduce sexual transmission of HIV at population level? If yes, which combination?

In 2009 a mathematical modelling exercise using a hypothetical population and assumptions relating to the South African setting concluded that *"Universal voluntary HIV testing and immediate ART (regardless of CD4 count) combined with present prevention approaches, could have a major effect on severe generalised HIV/AIDS epidemics"* (23). Two accompanying commentaries addressed how and whether this was feasible to deliver in the 'real world' (24, 25). The modelling exercise showed that HIV transmission could be substantially reduced within a few years. The authors argued that current clinical practice, which in nearly every setting, relies on CD4 counts and advanced HIV disease as the trigger to introduce ART, limits its preventive efficacy by leaving key points in HIV's natural history to go unchecked by effective VL-reducing treatment (these key points are the peak VL at seroconversion and the sustained period of somewhat elevated VL set point during the asymptomatic period). Dodd et al developed a deterministic mathematical model to investigate the impact of test-and-treat interventions under a variety of assumptions about the epidemic (26). They showed that such an intervention could substantially reduce HIV transmission, but that the impact might depend on the epidemiological context (notably determined by the sexual partner network, such as heterogeneity, concurrency and mixing).

Providing that a universal test and treat approach is appropriate, the question of the choice of ARV drug combination for wider and prolonged use becomes central. This ARV regimen should fulfil the following criteria: 1) Appropriateness for all CD4-cell strata to simplify its use; 2) Minimal side effects in otherwise "healthy" patients to avoid treatment drop-outs; 3) High potency to maximize effect on transmission; 4) High genetic barrier to minimize acquisition of viral resistance; 5) Sustainability for many years to limit and delay switches to second-line ARV regimens; 6) Low pill burden to facilitate treatment adherence; 7) Minimal laboratory requirements for follow-up; 8) Safety; 9) Affordability and 10) Coverage of special populations (TB co-infection, hepatitis B co-infection, pregnant women). A fixed-dose-combination (FDC) of (efavirenz [EFZ 600mg / tenofovir disoproxil fumarate [TDF] 300mg / emtricitabine [FTC] 300mg) available in South Africa as Atripla® or other generic forms administered once daily fulfils most of these criteria.

Not only may earlier treatment reduce HIV incidence at a population level, it may also benefit the individual. The long-term benefits of starting ART earlier would likely be even more important in settings where the incidence of life-threatening HIV-related diseases occurring at relatively high CD4 levels (tuberculosis, invasive bacterial diseases, and possibly malaria) is substantial, a typical situation in most sub-Saharan Africa including South Africa. Two randomised clinical trials have been designed or are in progress to address the "when to start" question in different settings and at different CD4 levels. The TasP trial addresses

the same issue but expands it further, although prevention of new infections is its "raison d'être" (26).

1.2 Study hypothesis

HIV testing of all members of a community, followed by immediate ART initiation of all HIV-infected individuals regardless of immunological or clinical staging will prevent onward sexual transmission and reduce HIV incidence in this population.

1.3 Study design

TasP will be **a population-based cluster-randomised trial (RCT), with small communities used as the units for randomisation. It aims at evaluating whether ART delivered at an earlier stage of HIV infection than currently recommended can prevent onward transmission of HIV.** This effect on HIV incidence among HIV-negative subjects will be the direct consequence of the treatment initiation among those non-eligible for ART according to the current standards. TasP aims to address this very important question and **produce such evidence, from a rural area in KwaZulu-Natal, the province with the highest HIV incidence and antenatal prevalence in South Africa, the country with one of the highest HIV burdens in sub-Saharan Africa, the continent where 70% of new HIV infections occur**.

1.4 Trial phases

The TasP trial will be implemented in **a two-phase approach**:

- The **first phase** will be conducted in a selected number of clusters (see section 8). All trial procedures to be implemented in this first phase will be as planned in this full trial protocol.
- If in the first phase the procedures and approach are shown to be feasible and acceptable and if the aims of the trial are still deemed relevant within the context of international research advances and the research strategy of the Africa Centre and partner institutions, and in agreement with the Steering Committee (SC), recommendation of the Scientific Advisory Board (SAB) and the Data Safety Monitoring Board (DSMB), the protocol will be implemented in the remaining clusters (see section 8) in the **second phase.**

1.5 Expected results of the trial

We aim to contribute to the evidence-base that a Treatment as Prevention approach can be applied to an entire population with a high incidence of HIV infection, irrespective of the stage of HIV disease and circumstances of HIV testing of participants in that population, and that Treatment as Prevention contributes to a reduction in HIV incidence while yielding further population and individual benefits.

The evidence obtained should be strong enough for this approach to be replicated in a wide range of resource-limited settings, to have policy implications to result in wide implementation.

2. Trial setting

2.1 Hlabisa sub-district

The trial will be conducted in Hlabisa sub-district, Umkhanyakude district, Northern KwaZulu-Natal, South Africa (Figure 1). The Hlabisa health sub-district is a rural setting of 1 430 km^2 in size, with a population of approximately 220 000 Zulu-speaking people of whom 3.3% are located in a formal urban township (KwaMsane), 19.9% in peri-urban areas and the remainder (76.8%) classified as living in a rural area. The rural population lives in scattered homesteads that are not concentrated into villages or compounds (as would be the case in many other parts of Africa).

Figure 1
Hlabisa sub-district, uMkhanyakude district, Northern KwaZulu-Natal, South Africa (shaded area is the area of the Africa Centre surveillance)

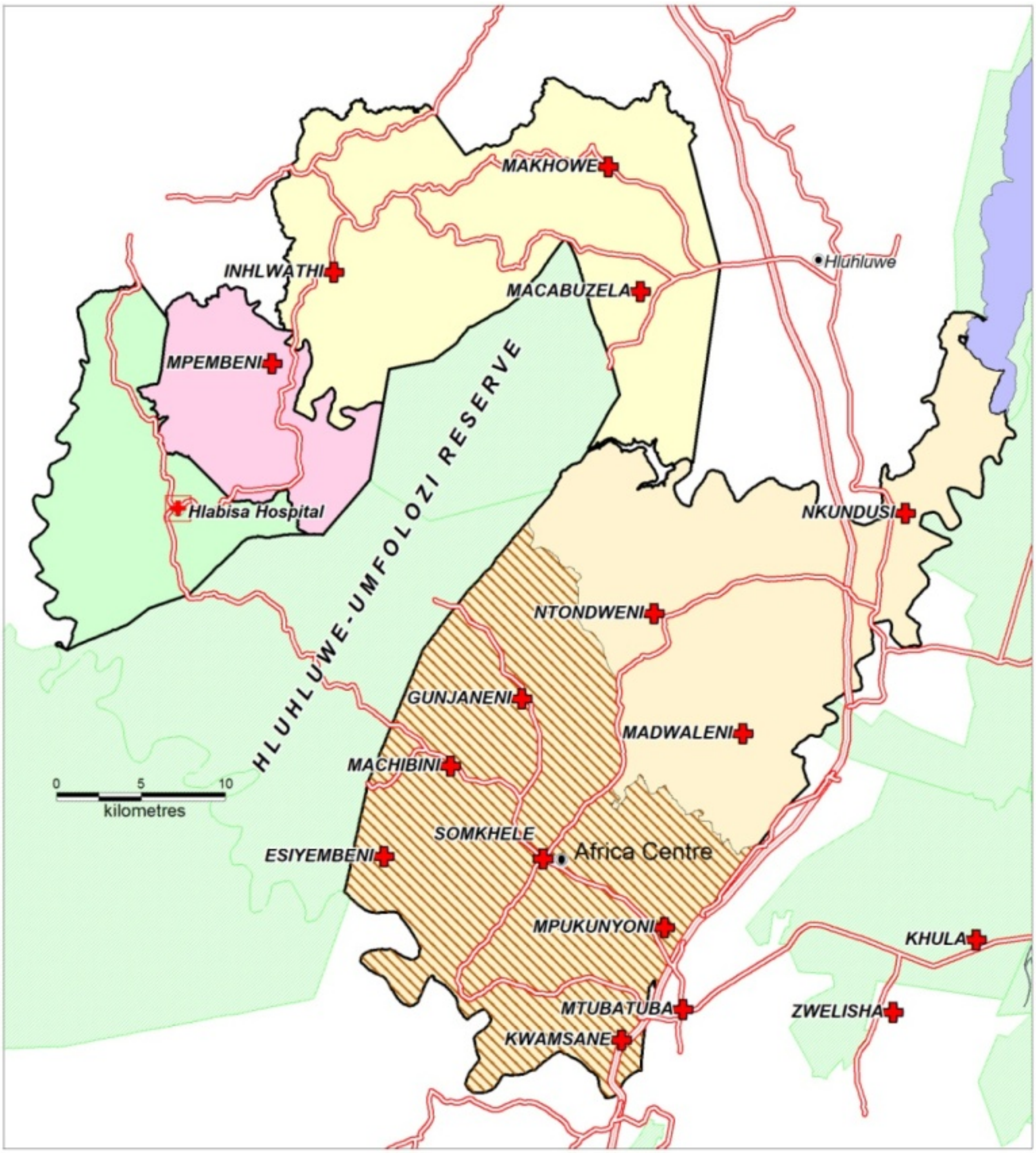

Health facilities in the Hlabisa sub-district are provided by one central community hospital (300 beds) and 17 fixed primary health care clinics

(Department of Health clinics), which provide the bulk of the health care for the population of the sub-district. All clinics provide care for minor ailments, family planning services, antenatal and postnatal care, conduct deliveries, treat sexually transmitted diseases, provide child immunisations, diagnose and manage TB, and care for chronic illnesses such as diabetes and hypertension (and HIV treatment and care, see below section 2.3). In addition to the 17 fixed clinics, 31 mobile clinic points are provided twice monthly, mainly for childhood immunisations and maternal and child health care. The sub-district is also serviced by approximately 130 community health workers, each of whom is expected to regularly visit a group of assigned homesteads.

2.2 The South Africa HIV treatment programme

The HIV treatment programme in South Africa is the largest in the world, with 2 million individuals estimated to have been receiving HAART in mid-2012 (27) (HSRC July 2013). One key aim of the current National Strategic Plan for HIV is to provide access to treatment, care and support to 80% of HIV-infected people and there has been significant scale-up of activity in the past few years (28). There remains, however, a large unmet need for treatment, with an estimated coverage in 2009/2010 of approximately 58% of those eligible for treatment, although this varies across districts (28).

The national response to HIV and the development of implementation plans is informed by the national strategic plan on HIV, STIs and TB (28) (ref) and the delivery of ART is guided by the recently updated National Antiretroviral Treatment (29). Until early 2010, HIV-infected adults were eligible for ART if CD4 cell count <200 cells/mm^3 or WHO stage IV condition. In April 2010, new guidelines were implemented where pregnant females and individuals with TB disease became eligible for ART if CD4 cell count <350 cells/mm^3, and in August 2011 these guidelines were further extended to eligibility starting at 350 for all HIV infected people in addition to all those with WHO stage 3 or 4 disease or anyone with MDR or XDR TB. The 2013 South African revised antiretroviral treatment guidelines also include all pregnant women to be eligible for ART for the duration of pregnancy and breastfeeding (29) (see Appendix 15.1). The current first-line drug regimens in use in South Africa are TDF + 3TC/FTC + EFV/NVP (see Appendix 15.1).

2.3 Hlabisa HIV Treatment and Care Programme

The Hlabisa HIV Treatment and Care Programme is a partnership between the KwaZulu-Natal Department of Health (DoH) and the Africa Centre, aiming to provide an accessible, equitable and comprehensive service to all people living with HIV infection (30). Established in 2004, the service is decentralized to all 17 primary health care clinics in the sub-district, and is nurse- and counsellor-driven, with scheduled physician visits to initiate patients on treatment and manage clinical problems. Recently, agreement was reached regarding nurse-led initiation and a number of nurses within the programme are enrolled on training programmes to allow this to happen. By September 2011, over 18,000 HIV-infected people eligible for treatment had started HAART; patients' eligibility is determined by South African guidelines (see section 2.2). There are no waiting lists for treatment initiation, approximately 250 adults and 30 children are started on treatment each month, and around 2,500 voluntary counselling and testing sessions are conducted monthly in the clinics or within the mobile and home-based testing services. In addition to those on treatment, more than 20,000 individuals are being monitored prior to requiring therapy. The Hlabisa HIV

Treatment and Care Programme also includes TB screening and treatment, PMTCT, PITC and VCT services, the latter delivered in the fixed clinics, as well as through mobile and home-based units (30). For individuals on ART between 2004 and 2008, retention in care at one year in the programme was 84.0% and mortality in the first year was 10.9% (31). Recent evidence shows a 46% decrease in early mortality for those who initiated ART in 2011/12 compared to the reference period 2008/9 (32).

2.4 The Africa Centre for Health and Population Studies

Hlabisa sub-district hosts the Wellcome Trust-funded Africa Centre for Health and Population Studies, a research institute of the University of KwaZulu-Natal, with a focus on HIV epidemiology and prevention (www.africacentre.com); the Centre partners with the Department of Health in the delivery of HIV treatment and care in the Hlabisa sub-district. A core activity of the Africa Centre is the longitudinal socio-demographic (bi-annual) and HIV (annual) surveillance in a geographically defined area in the south of the sub-district, covering about 40% of the area and of the population in the sub-district – the Demographic Surveillance Area (DSA) (see shaded area of Figure 1).

The TasP trial will take place in the sub-district outside of the Africa Centre surveillance area, so that the on-going HIV surveillance in the Africa Centre area can continue to monitor changes in behaviours and risks over time in a population very similar to the one where the trial will take place. The trial area contains six of the 17 fixed primary health care clinics in the sub-district.

3. Trial objectives

3.1 Overall objectives of the trial (first and second phase)

3.1.1 Main objective

- To compare the effect of ART initiated immediately after HIV diagnosis, irrespective of CD4 count criteria versus South African guidelines, on the reduction in incidence of new HIV infections in the general population in the same setting.

3.1.2 Specific objectives

Among all participants

- To compare the acceptability and feasibility of providing HIV testing to all members of a community between the two trial arms, and more specifically:
    - Acceptability/uptake of initial and repeat HIV counselling and testing (see section 7.3.1.1)
    - Behavioural changes at individual level: sexual behaviours, prevention practices, disclosure (see sections 7.3.1.2 and 7.3.1.3)
    - Community awareness, attitudes and behaviours (see section 7.3.1.5).
    - Societal response (see section 7.3.1.6)
    - Household expenditures, cost-effectiveness and other economic consequences of the trial intervention at individual level (see section 7.3.1.4)

Among HIV-infected participants only

- To compare acceptability and uptake of entry into care and ART between the two arms
- To compare participant retention, mortality and morbidity, TB, virological treatment failure, acquired HIV drug resistance, toxicity and cases of vertically-acquired HIV infections between the two arms (see section 7.3.2)
- To compare HIV testing experience, ART knowledge and perception, self-reported adherence to ART, quality of life, between the two arms (see section 7.3.2)

Within the health system

- To evaluate the challenges faced by the health care system and health care professionals in providing the trial intervention and coping with the increased number of trial participants (see section 7.5)

3.2 Objectives specific to the first phase

3.2.1 Main objective

- To validate and update the parameters of the model used to estimate the trial sample size and HIV incidence, in terms of: uptake of HIV testing, linkage to care upon HIV diagnosis, internal migration and ART initiation.

3.2.2 Specific objectives

Among all participants

- To estimate the acceptability and feasibility of providing HIV testing to all members of a community, and more specifically:
    - Acceptability/uptake of initial and repeat HIV counselling and testing (see section 7.3.1.1)
    - Behavioural changes at individual level: sexual behaviours, prevention practices, disclosure (see sections 7.3.1.2 and 7.3.1.3)
    - Community awareness, attitudes and behaviours (see section 7.3.1.5).
    - Societal response (see section 7.3.1.6)
    - Household expenditures, cost-effectiveness and other economic consequences of the trial intervention at individual level (see section 7.3.1.4)

Among HIV-infected participants

- To estimate acceptability and uptake of entry into care and ART
- To estimate participant retention, mortality and morbidity, TB, virological treatment failure, acquired HIV drug resistance and toxicity (see section 7.3.2)
- To estimate HIV testing experience, ART knowledge and perception, self-reported adherence to ART, quality of life (see section 7.3.2)

Within the health system

- To evaluate the challenges faced by the health care system and health care professionals in providing the trial intervention and coping with the increased number of trial participants (see section 7.5)

Other objectives

- To calculate the incidence of HIV infections in the general population in the clusters selected for the first phase to confirm the assumptions made in the sample size calculations for the main trial
- To better define the trial procedures on the basis of experience in the first phase as the acceptability of HIV testing and entry into care may present unexpected challenges
- To revise the protocol, if changes in national ART guidelines or new scientific evidence on the effect of early ART were to become available.

4. Overall trial plan

4.1 Description of the two trial phases

4.1.1 First phase

The first phase of the trial is planned over a 24-month period (see section 4.2). The aim of this first phase is to validate the hypothesis defined for the overall trial design (number of clusters, number of participants, incidence, HIV prevalence) and to verify the feasibility and acceptability of the intervention within the community.

The first phase will be conducted on a limited number of participants (n=10 000) and a limited number of clusters (2×5, see section 8).

In the initial four clusters, three rounds of home-based HIV testing of six, four and four months will be conducted. HIV-infected participants identified in all clusters will be followed-up between 7-24 months depending on the time of entry in the trial (first or third HIV testing rounds, see Figure 4).

For the additional 6 clusters, 2 rounds of home-based HIV testing of six months each will be conducted and this will coincide with the last 12 months of follow-up in the initial 4 clusters. Follow up of HIV-infected participants in these 6 clusters will be 1-12 months in the first phase.

4.1.2 Second phase

If in the first phase the procedures and approach are shown to be feasible and acceptable, if the aims of the trial are still deemed relevant within the context of international research advances and the research strategy of the Africa Centre and partner institutions, and in agreement with the Steering Committee (SC) and recommendation of the Scientific Advisory Board (SAB) and the Data Safety Monitoring Board (DSMB), the trial will be rolled-out to the remaining clusters (n=12, see section 8) in the second phase.

Four HIV testing rounds of six months each will then be conducted in all 22 clusters. HIV infected people from the phase 1 will continue to be followed in the TasP trial clinics until the trial ends when their care will be transferred to the programme as appropriate.

4.2 Trial timelines

Figure 2 summarises the timeline of the overall trial, first and second phase.

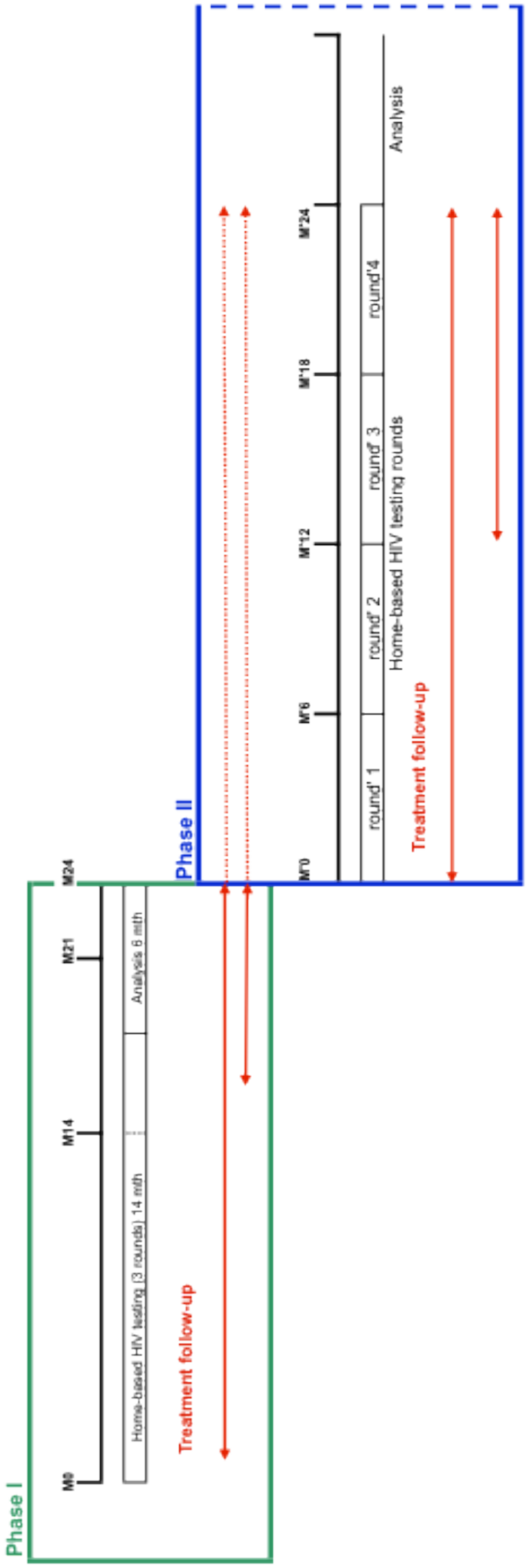

Figure 2
Timeline for the overall TasP trial

4.2.1 First phase (2011-2013)

For the initial 4 clusters

- October 2011 – February 2012: Preparation of the trial, recruitment and training of staff, logistics, ethics application, liaison with Department of Health.
- March – October 2012 inclusive: First round of HIV testing and enrolment in the four (2×2) clusters (this first round will take six months to allow registration of household members and formal introduction of the trial to households, etc.).
- Nov 2012 – August 2013: Two further rounds of HIV testing of four months each

For the additional 6 clusters

- August – December 2012: preparation for the addition of 6 new clusters
- January 2013 - February 2014: Two rounds of HIV testing of six months each

For all 10 clusters

- September 2013 – April 2014: Data analysis and review of results to date to inform decision regarding continuation of the trial. The final decision will be made by the SC according to the recommendations of the SAB and the DSMB.
    - In case of discontinuation: HIV-infected participants enrolled in the trial clinics will be transferred to the Hlabisa ART programme.
    - In case of continuation: HIV-infected participants will be followed-up during the second phase for a further 24 months (see section 6.4)

4.2.2 Second phase (2013-2016)

- November 2013 – March 2014: Preparation of the trial in the 12 new clusters
- April 2014 – March 2016: Enrolment in the 12 (2×6) new clusters and follow-up in the 10 phase 1 clusters during four rounds of HIV testing of six months each
    - April – December 2016: Data analysis and publication of final results according to the recommendations of the SAB and the DSMB

4.3 Description of the trial participants

Trial participation will be offered to all individuals meeting the following criteria:

- aged ≥16 years;
- member of a household in the designated cluster (head of household defines the membership status in Zulu culture);
- able and willing to give written informed consent for trial participation and/or HIV counselling and testing.

Note: Individuals considered unable to provide informed consent will include those with severe uncontrolled psychiatric disorders, and those with neurological impairment resulting in an inability to participate in the informed consent process.

5. Trial components

The TasP trial consists in HIV testing of all members of the community at regular intervals (component 1) and comparing two ART initiation strategies for HIV-infected participants (component 2) as summarised in Figure 3 below.

Figure 3
Description of the different components of the TasP trial

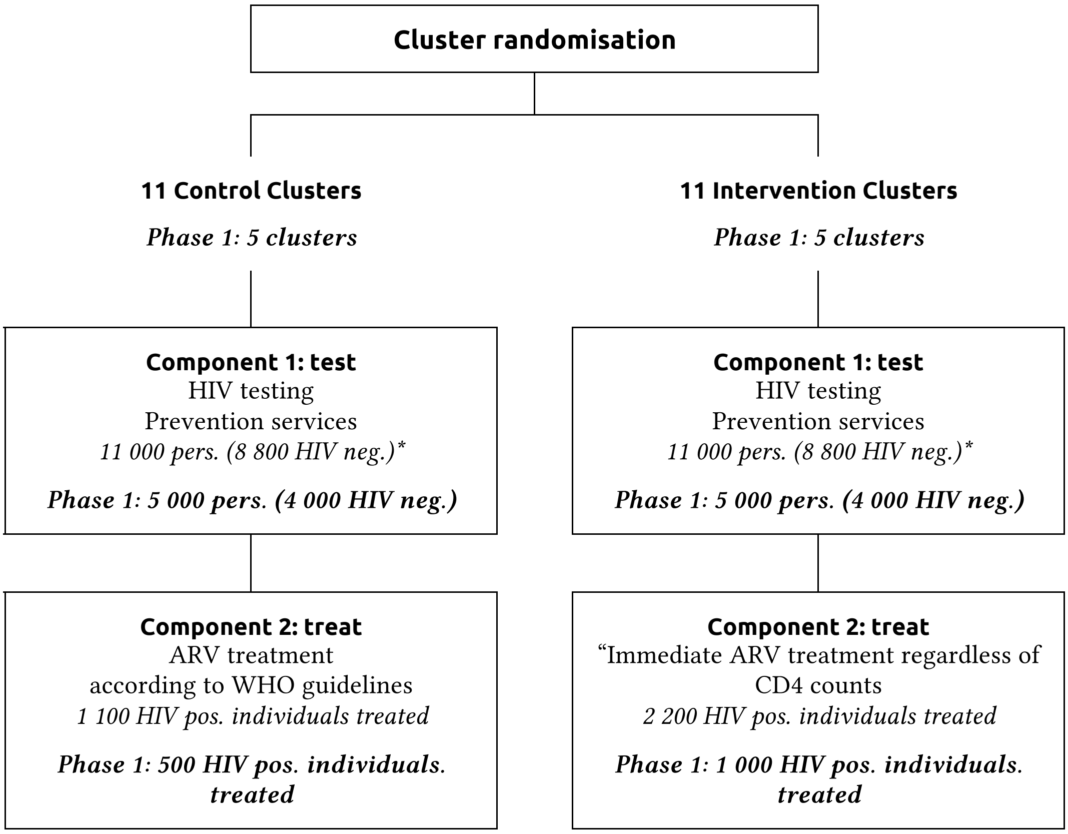

* *expected number of subjects within the 22 clusters.*

5.1 Component 1: HIV counselling and testing and comprehensive prevention programme

This first trial component will be identical in both intervention and control clusters:

- Provision of HIV counselling and testing to all members of the trial clusters (see section 6.2.2)
- Access to a full set of preventive services for all trial participants, services already available in DoH clinics for HIV-negative participants and made available within the trial clinics for HIV-infected participants; this will include Information, Education and Communication (IEC) and condom distribution, circumcision services, syndromic management of STIs and post-exposure prophylaxis, family planning (see section 6.3)

5.2 Component 2: ART for HIV infected participants

Within the second component, HIV-infected patients identified within the trial will be offered ART according to different eligibility criteria between the intervention and control clusters:

- in the intervention clusters: all HIV-infected adults will be offered ART regardless of their immunological and clinical staging;
- in the control clusters, HIV-infected people will be assessed clinically and immunologically and when eligible for treatment as per South African guidelines will be offered ART.

6. Trial conduct

6.1 Preparing the community

At the beginning of phase 1, a detailed community entry, education and trial promotion strategy that builds on the Africa Centre's extensive community engagement programme will be implemented based on the preparatory fieldwork conducted during late 2010, funded by the ANRS and the Africa Centre.

Key elements of the Africa Centre's existing and ongoing community engagement programme that provide opportunities to promote the trial include: bi-weekly sponsored community 'edu-tainment' road shows; annual community soccer and netball tournaments; annual community 'fun-run'; a widely circulated community magazine in isiZulu *(Umbiko)*; participation in a local community radio and a new programme of 'edu-tainment' music CDs distributed and played by operators of the local mini-bus taxi association.

The funded exploratory work involved a community consultation process using in-depth interviews and consumer research panels with a range of key community informants meeting several times during the pre-pilot phase to determine what additional community education and awareness activities are needed for the first phase. The resulting community entry strategy for the trial itself will be developed jointly by the investigators and the Africa Centre's Community Liaison Office and tested with the community research panels in the preparatory period of the trial (see section 7.4.5).

6.2 Baseline assessment, enrolment of participants, HIV counselling and testing

6.2.1 Approaching households, obtaining individual consent, and administrating population-based questionnaires

The procedures presented below apply to each round of HIV testing. They are the same for the intervention and control clusters, for phase 1 and for phase 2.

All households within each of the trial clusters will have been identified and GPS coordinates noted. A team of trial community testers, all of whom will be DoH-trained VCT counsellors, will approach the household and seek permission to enter from the household head (or most senior household member present if head is absent). After entering, the testing team will explain the trial and the procedures for testing and seek the permission of the household head to offer trial participation and HIV counselling and testing to adult members of the household. Specific information sheets for each component will be provided to all individuals (See Appendix 15.4).

Once the head of household has agreed to allow the team to enter the household, he/she will be asked to complete the *Tasp household registration questionnaire* (THR) on the netbook and the *TasP household information assets questionnaire* (HHI) (see section 7.4.1).

Once permission to proceed has been granted by the head of household, a private space will be identified and all individual adult household members will be invited to give written permission to complete the *TasP home-based individual questionnaire* (IQ), see section 7.4.1), with/without anonymous sampling of blood for HIV surveillance, with/without undergoing confidential HIV counselling and testing (see section 6.2.2 below). Individual household members may participate

in the trial by agreeing to the individual questionnaire with/without HIV testing (separate consent forms, *Home-Based Individual Questionnaire and DBS Consent Form – CZ1 – and Home-Based HIV Testing Consent Form – CZ2*). People who do not want to be tested for HIV in the household can attend any of the DoH clinics, or the trial clinic, for testing and will be informed of this possibility.

The content of IQ is described section 7.4.1.

Participants who give consent to the anonymous collection of blood for HIV-related tests will have blood collected by field workers by finger prick and stored on filter paper as dried blood spots (DBS) (see testing procedures section 6.4.6).

The HIV counselling and testing procedures are described below (see section 6.2.2).

In each round of testing, the team will be able to return on up to two more occasions to offer trial participation and/or HIV testing to members of the household who were not present during the initial visit, after which the untested members of the household will receive a written invitation to attend the trial clinics where HIV counselling and testing will also be available.

For participants who decline both HIV counselling and testing and the questionnaires, we will obtain basic demographic data from the household head. Participants who decline either testing or the questionnaire in the follow-up rounds will be asked to provide key, basic socio-demographic, knowledge of HIV status and HIV testing history data to the testing team (see section 7.4.1).

6.2.2 HIV counselling and testing procedures

Different HIV testing strategies currently operate in the trial area. HIV testing is available in DoH hospital and the 17 fixed primary health care clinics. This includes VCT clinics and PICT (provider initiated counselling and testing), including the routine testing of pregnant women and TB patients in clinics. In addition, some non-government organisations (NGOs) provide testing at fixed sites in Mtubatuba town and KwaMsane and may take testing to community venues and events. Further, the DoH/Africa Centre partnership programme, the Hlabisa HIV Treatment and Care Programme (see section 2.3), provides the largest and most comprehensive range of HIV testing services available, employing DoH-trained counsellors. The testing options available in the Hlabisa HIV Treatment and Care Programme include mobile clinics, offering testing in communities routinely, mobile testing clinics at community events, and home-based VCT offer in households across the whole of the Hlabisa sub-district, an initiative in the Programme started in 2009 which has proved to be highly acceptable with a high uptake rate (see section 1.1.4).

Within the TasP trial we will extend the provision of home-based VCT in households on a more regular basis with six monthly visits and combine the current range of community and clinic testing options, thus achieving a maximum (near universal) HIV testing coverage in the area and increasing options for repeat HIV testing.

During each HIV testing round, individuals providing written informed consent will receive pre-test HIV counselling privately and confidentially, delivered by a DoH trained counsellor.

Rapid HIV testing will be performed. The screening test will be Determine, and the confirmatory test will be First Response. Test results will be provided approximately 20 min after testing.

All participants who test HIV-positive will be referred to the trial clinic for further assessment, including a point-of-care CD4 count. Results from the baseline viral load test will confirm HIV positive status. Where there is discrepancy in results between the VL test and the rapid antibody test, the DBS from the home-based testing stored in the Africa Centre virology laboratory can be accessed for confirmatory antibody testing.

The individual post-test HIV counselling session will take place as per routine DoH procedures, covering the prevention of acquisition of HIV for negative people and the implications of HIV infection for positive people.

The same home-based HIV VCT procedures will be used in successive rounds of community testing to ensure a high uptake of testing and repeat testing overall, particularly as those who decline testing in one round become familiar with the procedures.

A standardised referral procedure will be developed to provide an opportunity to children and minors to be tested according to the South African guidelines at the DoH clinics and other facilities.

Figure 4 summarises the home-based procedures at each round of testing.

Figure 4
Home visit chart.

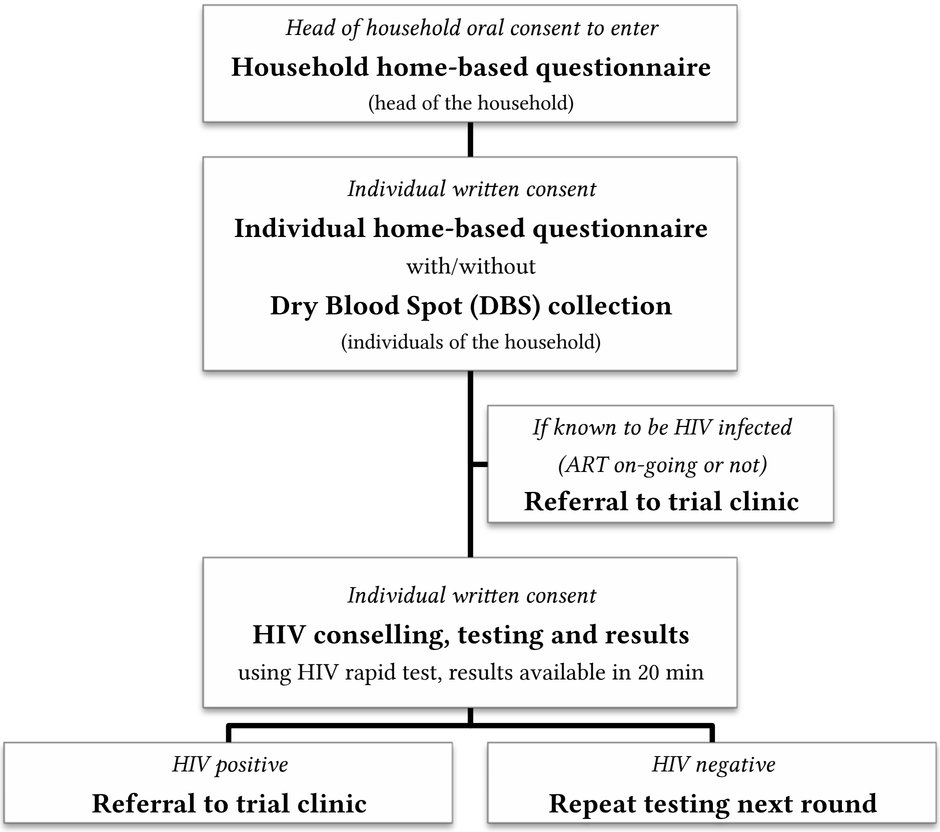

6.2.3 Referral and linkage to care procedures

During each home-based VCT round, at the end of the counselling and testing session, all participants will be given the same referral/prevention card (regardless of their test result and/or parts of the trial they may have consented to). The referral card will contain information about the TasP trial clinics and the available services (treatment and care, VCT, condoms, referral to circumcision,

etc.). Each participant will be asked to take their referral card with them when they attend clinics in order to identify them as a trial participant. A thumbprint will be taken at the TasP clinic to confirm a participant's correct identity and their correct service eligibility (i.e. treatment, care and prevention or VCT and prevention).

During post-test HIV counselling, HIV-infected participants will all be referred to the trial clinic in their cluster for further assessment as to their treatment eligibility (see section 6.4 below). HIV-infected participants will be informed that, if they have not attended the trial clinic within 3 months of referral, they will be contacted by a member of the study team (tracking team) either by phone or during a home visit for additional counselling and support to facilitate linkage to care.

A trained research psychologist will work with the tracking team to explore cognitive, psychological and normative aspects, which determine clinical linkage among individuals who test positive in the trial, but have not linked to care within 3 months of testing. All participants will be asked a screening question regarding the information received on the trial to test recall and understanding of opportunities to link to care. Thereafter psychological assessments will be completed using short validated screening tools with participants in their homes. This assessment includes psychological measures of depression, anxiety, somatisation (using the PHQ9; PHQ15 and GAD7) and health perceptions (MOS-36); normative aspects including stigma (HIV Stigma Scale - Revised) and family functioning (FAD). A brief cognitive screen (Oxford Cognitive Screen) on executive function will be administered using a tablet. This assessment will take approximately 45 minutes.

6.3 Preventive interventions

All participants, whether from intervention or control clusters, will have access to a full set of preventive interventions described below. HIV-negative participants will access these services, as does the general population, within DoH fixed clinics. All HIV-infected participants will be provided these services during their follow-up visits within the trial clinics.

6.3.1 Information and education, condom promotion and distribution

Specific behaviour change information and education, condom promotion and distribution activities, in line with what is currently provided in the Hlabisa HIV Treatment and Care Programme, will operate alongside those sponsored by national and provincial governments as well as various NGOs. VCT counsellors and clinic staff are trained in essential behaviour change counselling and available to provide this to trial participants both during VCT sessions or clinic visits. Social marketed condoms are currently routinely available through the DoH clinics as well as for purchase in small convenience shops (*spaza* shops) in the community, more formal shops, petrol stations and other outlets. On the basis of our audit and mapping of prevention and HIV testing services additional condom distribution plans for both intervention and control clusters will be implemented.

6.3.2 Male circumcision

The phased implementation of a national programme of male circumcision has started in South Africa and KwaZulu-Natal's roll-out started at test sites in 2010. During 2011, the plan involved the setting up of mobile surgical facilities (in

'camps') where a trained physician performs the procedure for young adult males. The Africa Centre works closely with the DoH in implementing the programme in the sub-district as part of the Hlabisa HIV Treatment and Care Programme. However, since then the mobile camps have not been regular, and the programme has been taken over by NGO's working in the district; in addition circumcision is available from the district hospital, but there is limited capacity. Male circumcision is also available from private medical practitioners in the area and is covered by some employees' medical-aid schemes. Rates of male circumcision among participants in the Africa Centre's HIV surveillance have been low.

6.3.3 Syndromic management of sexually transmitted infections

All HIV-infected participants (male and female) will undergo a standardised STI symptom screen at the time of enrolment in the trial clinic, and at regular intervals once enrolled in HIV care. Symptom screen for women will include: lower abdominal pain, vaginal discharge, dysuria, and genital ulcers. Symptom screen for men will include: urethral discharge/dysuria, genital ulcers, and scrotal swelling/pain. This will be treated syndromically according to South African guidelines. The importance of partner treatment will be stressed and partner notification slips will be issued as per routine practice. All management will be in line with national guidelines and no additional diagnostic services will be included in the trial phase.

6.3.4 Post-exposure prophylaxis after sexual assault

All incidents of sexual assault for trial participants will be reported immediately to the police and management will follow standard guidelines. Post-exposure prophylaxis (PEP) will be offered to HIV-negative participants reporting penetrative anal or vaginal sexual assault who present within 72 hours**.** This consists of ZDV 300 mg bd + 3TC 150 mg bd (Combivir) and LPV/r (Kaletra) 400/100 mg bd for four weeks and will be administered either at Hlabisa hospital or at the existing crisis centre.

6.3.5 Family planning

All female participants will be asked about their use of family planning methods at the time of trial enrolment. Family planning advice will be offered to both HIV-positive and HIV-negative female participants, as part of the post-test counselling within the household. For HIV-infected people, there will be more opportunity for discussion at the trial clinics, whereas HIV-negative people will be referred to the DoH services. Dual methods (hormonal contraception + barrier) will be encouraged for all participants with no immediate pregnancy intentions. Any request to commence contraception will require referral to the fixed DoH primary health care clinic – this will be facilitated with a standardised referral form. HIV-negative participants will ordinarily be offered either oral contraceptives (several options) or injectable contraceptive (medroxyprogesterone acetate 150mg, 12-weekly). HIV-positive participants will be encouraged to use an injectable contraceptive.

6.4 Management and care of HIV-infected participants

6.4.1 Pre-treatment assessment visit

All participants newly diagnosed with HIV infection during any of the home-based VCT rounds will be referred to their trial clinic for immediate further assessment. Trial clinics will be informed about whom to expect.

Trial clinics (one per cluster) will be situated in close proximity to residences (< 45 minutes walking distance for all participants within the cluster).

These trial clinics will be staffed by a counsellor and a nurse. A referent physician will be available weekly at each clinic and on-call if necessary.

At the trial clinics, **all newly documented HIV-infected participants** will be provided standard counselling/ART education and adherence sessions by the ART counsellor and nurse, over one to three visits. They will be asked using separate documents to: 1) provide self-reported information and an anonymous blood specimen for viral load testing (see below) and 2) receive care and/or treatment at the trial clinics as per DoH standards. The HIV-infected participants consenting to treatment will then undergo a clinical evaluation which includes medical history, physical examination, WHO clinical staging (review of current and previous morbidity), basic anthropometry (weight and height), as per DoH procedures using the following CRFs: *Clinic baseline visit - Counsellor (CBC), Clinic Follow-up - Counsellor (CFU), Clinic history and examination-nurse (CHE).*

Patients will be referred to the ART counsellor to respond to the *Social science clinic-based baseline questionnaire* (SCB) and specifically questions regarding HIV testing experience, ART perception, disclosure and economic situation (see Table 3). This baseline interview will take place anytime during the pre-treatment visits.

In the intervention clusters, the clinical evaluation will involve completion of the following:

- Blood tests: CD4 count (point of care*), VL, haematology (full blood count), biochemistry (urea, creatinine, electrolytes, liver function tests, glucose, lipids), hepatitis B surface antigen (HBsAg) and plasma storage,
- Urine tests: dipstick urinalysis, beta hCG to all women of childbearing age.
- Other screening: Sputum will be collected for TB investigation (microscopy and culture), as appropriate following screening on clinical symptom

In the control clusters, the pre-treatment assessment will be decided after obtaining the CD4 count measured at point of care and/or according to the clinical staging (see ART initiation criteria in section 6.4.1 below):

- Patients eligible for ART will undergo baseline investigations performed as above
- Patients not eligible for ART will have a blood sample collected for baseline VL measurement and storage; they will be invited to return to the study clinic in 4-6 months based on the CD4 count

All participants will be seen within two weeks of this W0 pre-assessment visit, to review this pre-therapeutic assessment and, if indicated, initiate treatment at W2.

In both clusters, **patients refusing ART** will be offered the opportunity to remain in care and will be asked to consent to six-monthly clinical assessment: describe (see section 6.4.4.3).

In both clusters, **participants already established on ART** from the Hlabisa HIV Treatment and Care Programme will be encouraged to be included in the trial and to transfer their care to the trial clinics. Participants already established on ART from private/other HIV treatment providers will be encouraged to take part in the trial monitoring procedures. They will be offered the option of 1) transferring follow-up to the trial clinic (the same follow-up procedures as for patients newly established on ART will then apply) or 2) continuing follow-up from their normal provider, without any discriminatory measures applied to them. During the home visit, all HIV-infected participants will be asked to give consent for their clinical records to be obtained from their normal provider should they choose not to attend the trial clinics. Information accessed would include ART status, ART regimen if on ART, CD4 counts and viral loads. This information will be used to estimate the ART treatment coverage in each cluster as this is directly linked to HIV transmissions. The nurse at each trial unit will liaise weekly with the Hlabisa HIV treatment programme clinics to ensure ease of information collection for patients who received clinical care outside of the trial clinics.

* *Note on CD4 point of care:* We will use the Alere PIMA™ device tool (http://pimatest.com/en/home.html) to measure CD4 in the clinics. The system has built-in quality control mechanisms, in addition to which the trial will develop its own quality control procedures (in association with CD4 laboratory, NHLS). Quality control will be performed on a 10% of participants selected by systematic random sampling using the enrolment log of each clinic as a sampling frame. All subsequent samples from the identified individuals requiring haematology assessment will also be assessed for CD4 count at the NHLS laboratory at Hlabisa hospital.

6.4.2 ART initiation criteria

In the intervention clusters, there are no immunological criteria for starting ART; all HIV-infected participants are eligible for ART.

In the control clusters, HIV-infected participants will be eligible for ART in line with the revised March 2013 South African guidelines (see Appendix 15.1).

- CD4 count ≤ 350 cells/mm^3 irrespective of clinical symptoms
- WHO clinical stage 3 or 4 irrespective of CD4 count
- MDR or XDR TB
- All pregnant women for the duration of pregnancy and breastfeeding (option B)

6.4.3 ART drugs used within the trial

6.4.3.1 Description of the drugs

All ART drugs that will be used in the trial are those recommended in the National Department of Health Adult HIV management guidelines (see Table 1).

The standard first-line drug regimen for HIV-infected participants will in both arms be the fixed drug combination (FDC) Tenofovir (TDF – 245 mg) + Emtricitabine (FTC – 200 mg) + Efavirenz (EFV – 600 mg) or Atripla® once daily. Participants who transfer their care while on the generic formulation of the FDC Tenofovir (TDF – 245 mg) + Emtricitabine (FTC – 200 mg) + Efavirenz (EFV – 600 mg) which is available in the government HIV treatment programmes will be able to continue on this but for logistic reasons could be switched to Atripla® as

they would then be less dependent on supplies from the government (See Appendix 15.1).

Table 1
Antiretroviral regimens proposed to TasP patients

**1st Line**		
All new patients needing treatment, including pregnant women	TDF + FTC (or 3TC) +EFV<br>Fixed drug combination (FDC) preferred	Replace EFV with NVP in patients with significant psychiatric co-morbidity or intolerance to EFV and where the neuro-psychiatric toxicity of EFV may impair daily functioning, e.g. shift workers.
Contraindications to EFV	TDF + (FTC or 3TC) + NVP	Use NVP based regimen: In patients with significant psychiatric co morbidity or intolerance to EFV and where the neuro-psychiatric toxicity of EFV may impair daily functioning, e.g. shift workers.
Contraindication to TDF	AZT+ 3TC +EFV or (NVP)	Renal disease or the use of other nephrotoxic drugs e.g. aminoglycosides
Contraindication to TDF and AZT	d4T + 3TC+ EFV (or NVP)	Renal disease and anaemia or the use of other nephrotoxic drugs, aminoglycosides
Contraindication to TDF, AZT and d4T	ABC + 3TC + EFV (or NVP)	Renal disease, anaemia, peripheral neuropathy, the use of other nephrotoxic drugs
Currently on d4T-based regimen	TDF + FTC(or 3TC) + EFV<br>FDC preferred	Mandatory if patients experience toxicity and patients who are at high risk of toxicity (high BMI or pregnant). Switch to TDF if virally suppressed and the patient has normal creatinine clearance, even if well tolerated.
**2nd Line**		
Management of virological failure		If plasma HIV RNA >1000 copies,<br>Check for adherence, compliance, tolerability and drug- drug interaction and assess psychological issues.<br>Repeat VL test 2 months later.<br>If plasma VL confirmed >1000copies change regime to second line therapy
Failing on a TDF-based 1st line regimen	AZT+3TC+ LPV/r	Patients with anaemia and renal failure switch to ABC
Failing on a d4T-based 1st line regimen	TDF+3TC (or FTC) and LPV/r	
Dyslipidaemia or diarrhoea associated with LPV/r	Switch LPV/r to ATV/r	
**Third line**		
Failing any 2nd line regimen	Specialist referral	
Should be expert and genotype resistance testing based decision and supervised care<br>Patients failing on second line therapy will be managed by an expert panel. The drugs for third line will be managed centrally. More discussion is required to deal with the modalities	Most likely regimen would be Raltegravir/Darunavir/ /Etravirine adjusted according to genotype Interpretation. Should be by expert and take into account prior exposure and predictable mutations	

6.4.3.2 Supply

Atripla® will be provided by MSD during the study period, All other drugs will be provided by the KwaZulu-Natal Provincial Department of Health according to the standard of care.

6.4.3.3 Drug handling and storage

Trial drugs provided and shipped by MSD will be held centrally at Hlabisa Hospital pharmacy, under the supervision of the trial pharmacist. Supplies will be delivered weekly to the treatment sites within the trial area and kept in secure storage facilities.

At each delivery, the pharmacist will record the trial patient identification code, the name of the drug, the number of the batch, the expiry date and the accountability on the Drug Accountability form.

All trial drugs will be labelled with the patient name as per South African standards, trial number, and dosing requirements.

Patients will be provided with 4-weekly supply of drugs initially. Participants who have undetectable viral loads and no ongoing medical problems will be allowed 8-weekly supply of drugs to decrease frequency of clinic attendance to save them time and transport costs.

Participants will be requested to return all empty bottles and to bring any bottles in use to their follow-up visits. Unused drug must be returned to the trial site if a participant withdraws from the trial. Unused drugs will be disposed of through the existing disposal mechanisms in place at Hlabisa Hospital pharmacy.

6.4.4 Patient follow-up

The follow-up procedures for HIV-infected participants will be the same in both intervention and control clusters.

6.4.4.1 ART initiation visit

ART initiation will take place ideally within two weeks of enrolment at the trial clinic, unless purposely delayed for clinical reasons (e.g. TB treatment).

The results of baseline investigations of all patients eligible for ART initiation and consent for treatment will be reviewed with the nurse. Nurses will initiate ART in uncomplicated cases. As per the Hlabisa HIV Treatment and Care Programme, there will be specific guidelines regarding clinician referral prior to ART initiation (e.g. for patients with persistent TB symptoms but negative sputum smear, and patients with abnormal renal or liver function). Appendix 15.5 presents a flowchart of the follow-up provided to HIV-infected participants in the intervention and control clusters.

For patients already on ART and transferred from private/other HIV treatment providers, their ART initiation date will be the date of their first visit to a TasP clinic (for the trial purposes, it will not reflect their duration on ART).

6.4.4.2 Treatment duration

Once the decision to initiate ART has been made, patients will receive ART and be followed-up within the trial for a maximum duration of four years depending on time of enrolment in trial (see Table 2).

Table 2
Follow-up calendar for HIV-infected participants eligible for ART

	W0	W2	W4	W6	W10	M3	M4	M5	M6	M7	M8	M9	M10	M11	M12	M13	M14	M15	M16	M17	M18	M19	M20	M21	M22	M23	M24
Consent	×																										
Medical history	×																										
**Nurse**																											
Physical examination	×			×	×	×	×	×	×	×	×	×	×	×	×	×	×	×	×	×	×	×	×	×	×	×	×
Weight, height	×					×			×			×			×			×			×			×			×
WHO clinical staging	×			×	×	×	×	×	×	×	×	×	×	×	×	×	×	×	×	×	×	×	×	×	×	×	×
Morbidity/hospi	×			×	×	×	×	×	×	×	×	×	×	×	×	×	×	×	×	×	×	×	×	×	×	×	×
TB/STI screening (if appropriate)	×			×	×	×	×	×	×	×	×	×	×	×	×	×	×	×	×	×	×	×	×	×	×	×	×
CD4 point of care	×					×			×						×						×						×
ART initiation		×							×						×						×						×
Adherence monitoring			×	×	×	×	×	×	×	×	×	×	×	×	×	×	×	×	×	×	×	×	×	×	×	×	×
**Laboratory**																											
HIV VL	×					×			×						×						×						×
Genotyping	As clinically indicated, in cases of confirmed virological failure																										
CD4 counts (quality control)	×								×						×						×						×
Haematology	×								×						×						×						×
Biochemistry (U&Es, LFTs)	×					×			×						×						×						×
HBsAg	×																										
Beta hCG	×					×			×						×						×						×
Urinalysis	×					×			×						×						×						×
Plasma storage (-80°C)	×					×			×						×						×						×
Blood volume (ml)																											
**Clinic-based survey**																											
HIV testing experience	×																										
ART perception, decision	×								×						×						×						×
Disclosure and couple	×								×						×						×						×
Social and community support	×								×						×						×						×
Stigma and discrimination	×								×						×						×						×
Health expenditure	×								×						×						×						×
Economic situation	×								×						×						×						×
Sexual behaviour									×						×						×						×
ART knowledge									×						×						×						×
Self-reported ART adherence									×						×						×						×
HIV quality of life									×						×						×						×
Satisfaction with care									×						×						×						×

TB: Tuberculosis; STI: Sexually Transmitted Infections; ART: Antoretroviral Treatment; VL: Viral Load; U&Es: Urea and Electrolytes test; LFT: Liver function tests.

	M24	M25	M26	M27	M28	M29	M30	M31	M32	M33	M34	M35	M36	M37	M38	M39	M40	M41	M42	M43	M44	M45	M46	M47	M48
Consent																									
Medical history																									
**Nurse**																									
Physical examination	×	×	×	×	×	×	×	×	×	×	×	×	×	×	×	×	×	×	×	×	×	×	×	×	×
Weight, height	×			×			×			×			×			×			×			×			×
WHO clinical staging	×	×	×	×	×	×	×	×	×	×	×	×	×	×	×	×	×	×	×	×	×	×	×	×	×
Morbidity/hospi	×	×	×	×	×	×	×	×	×	×	×	×	×	×	×	×	×	×	×	×	×	×	×	×	×
TB/STI screening (if appropriate)	×	×	×	×	×	×	×	×	×	×	×	×	×	×	×	×	×	×	×	×	×	×	×	×	×
CD4 point of care	×			×			×						×						×						×
ART initiation	×						×						×						×						×
Adherence monitoring	×	×	×	×	×	×	×	×	×	×	×	×	×	×	×	×	×	×	×	×	×	×	×	×	×
**Laboratory**																									
HIV VL	×			×			×						×						×						×
Genotyping	As clinically indicated, in cases of confirmed virological failure																								
CD4 counts (quality control)	×						×						×						×						×
Haematology	×						×						×						×						×
Biochemistry (U&Es, LFTs)	×			×			×						×						×						×
HBsAg																									
Beta hCG	×			×			×						×						×						×
Urinalysis	×			×			×						×						×						×
Plasma storage (-80°C)	×			×			×						×						×						×
Blood volume (ml)																									
**Clinic-based survey**																									
HIV testing experience																									
ART perception, decision	×						×						×						×						×
Disclosure and couple	×						×						×						×						×
Social and community support	×						×						×						×						×
Stigma and discrimination	×						×						×						×						×
Health expenditure	×						×						×						×						×
Economic situation	×						×						×						×						×
Sexual behaviour	×						×						×						×						×
ART knowledge	×						×						×						×						×
Self-reported ART adherence	×						×						×						×						×
HIV quality of life	×						×						×						×						×
Satisfaction with care	×						×						×						×						×

TB: Tuberculosis; STI: Sexually Transmitted Infections; ART: Antoretroviral Treatment; VL: Viral Load; U&Es: Urea and Electrolytes test; LFT: Liver function tests.

Any patient deciding to permanently discontinue ART will be offered the opportunity to transfer to the DoH fixed clinics while remaining under active follow-up in the trial: participants will be tracked during the household HIV testing rounds, their patient records may be accessed (see section 6.4.1).

During the first phase, all HIV-infected participants will be followed-up between 1 and 24 months. At the end of the first phase, HIV-infected patients will either be transferred to the Hlabisa ART programme if the trial stops, or followed-up until the end of the second phase.

6.4.4.3 ART follow-up visits

- **Participants receiving ART**

Participants initiating ART will be reviewed by the trial nurse monthly when they collect their medication until they have two consecutive undetectable viral loads measured three months apart following which follow up will become 8-weekly in those with no on-going medical issues.

Clinical evaluation (4-weekly or 8 weekly)

The nurse will document weight and proceed with a review of current morbidity and illnesses/hospitalizations since last visit (CHE form).

Interview/Questionnaire (six-monthly)

HIV-infected participants, whether on treatment or not, will be administered the social science clinic-based survey and interviewed at M6, M12, and 6-monthly until M48 (counting from entry into care), regarding specific topics according to visits (see Table 3). The clinic-based survey will be divided in two parts:

- one part administered by the ART counsellor who will ask non-sensitive questions such as disclosure or health expenditures: the *Social science clinic-based counsellor-administered questionnaire* (SCC);
- one part administered by an independent interviewer who will ask sensitive questions (which are subject to social pressure and thus to social desirability bias) such as sexual relations or adherence: the *Social science clinic-based independent interviewer-administered questionnaire* (SCI).

Here below in Table 3 is presented the distribution of topics to be asked either by the counsellor or interviewer.

Table 3
Topics of Social science clinic-based survey, by questionnaire

Topic	W0	M6	M12	M18	M24	M30	M36	M42	M48
HIV testing experience	C								
Economic situation	C	C	C	C	C	C	C	C	C
Health expenditures	C	C	C	C	C	C	C	C	C
ART perception and decision	C	C	C	C	C	C	C	C	C
Disclosure and couple	C	I	I	I	I	I	I	I	I
Social and community support	C	I	I	I	I	I	I	I	I
ART knowledge		I	I	I	I	I	I	I	I
Sexual behaviour		I	I	I	I	I	I	I	I
Self-reported adherence to ART*		I	I	I	I	I	I	I	I
HIV Quality of Life		I	I	I	I	I	I	I	I
Stigma and discrimination		I	I	I	I	I	I	I	I
Satisfaction with care		I	I	I	I	I	I	I	I

C: counsellor-administered questionnaire
I: independent interviewer-administered questionnaire;
** only for ART-treated patients.*

Adherence monitoring

Both an independent interviewer and the ART counsellor (who is not responsible for the treatment delivery) will monitor adherence at each visit for all participants receiving ART. This will include three categories of adherence measurement tools:

- Subjective (monthly): self-report (as per standard South African treatment guidelines, see section 7.3.2.5)
- Objective, technical (monthly): pill count
- Objective, biological (quarterly): viral load + resistance test if viral failure

Sub-optimal adherence will prompt an individual session with the HIV counsellor and nurse. Persistent sub-optimal adherence (over three consecutive visits) will prompt referral to the trial clinician. Specific problems may warrant referral to members of the Hlabisa HIV Treatment and Care Programme multidisciplinary team (pharmacist, social worker, psychologist, dietician, and home-based carers).

Laboratory assessments (M3, M6, M12, 6-monthyl up to M48)

Participants initiating a Tenofovir-based ART regimen such as Atripla will require monthly urea, electrolytes and creatinine for the first 3 months, at 6 months and then 6 monthly.

Participants initiating a Zidovudine-based ART regimen will require monthly FBC for the first three months, at 6 months and then 6 monthly.

Other screening (as required):

Sputum will be collected for TB investigation (GeneXpert, microscopy and culture), as appropriate following screening on clinical symptom

- **Participants not receiving ART**

Participants not receiving ART (not yet eligible for treatment or refusing treatment) will be referred to the trial nurse for pre-ART care and positive prevention services (see section 6.3). Their six-monthly clinical assessment will include:

- Clinical evaluation with physical examination, WHO clinical staging (review of current morbidity and illnesses/hospitalizations since last visit), STI screening, TB screening, pregnancy test if appropriate, weight.
- Biological assessment: CD4 counts (point of care) 4-6 monthly to check treatment eligibility in the control clusters.
- *Interview/Questionnaire:* HIV-infected participants will be administered the *clinic-based questionnaires* (SCC and SCI) and interviewed regarding specific topics according to visits (see Table 3).
- Based on their immunological status, treatment initiation will be proposed according to the procedures (pre-treatment assessment, treatment initiation and follow-up) described in sections 6.4.1, 6.4.2, 6.4.3 and 6.4.4 above.

Table 4
Follow-up calendar of HIV-infected participants not eligible for ART

	W0	M6	M12	M18	M24	M30	M36	M42	M48
Consent	×								
Medical history	×								
**Nurse**									
Physical examination	×	×	×	×	×	×	×	×	×
Weight, height	×	×	×	×	×	×	×	×	×
WHO clinical staging	×	×	×	×	×	×	×	×	×
Morbidity/hospi	×	×	×	×	×	×	×	×	×
TB/STI screening if appropriate	×	×	×	×	×	×	×	×	×
CD4 point of care	×	×	×	×	×	×	×	×	×
**Laboratory**									
Beta hCG	×	×	×	×	×	×	×	×	×
**Clinic-based survey**									
HIV testing experience	×								
ART perception	×	×	×	×	×	×	×	×	×
Disclosure and couple	×	×	×	×	×	×	×	×	×
Social and community support	×	×	×	×	×	×	×	×	×
Stigma and discrimination	×	×	×	×	×	×	×	×	×
Health expenditure	×	×	×	×	×	×	×	×	×
Economic situation	×	×	×	×	×	×	×	×	×
Sexual behaviour		×	×	×	×	×	×	×	×
ART knowledge		×	×	×	×	×	×	×	×
HIV quality of life		×	×	×	×	×	×	×	×
Satisfaction with care		×	×	×	×	×	×	×	×

W0 = date of entry into care
TB: Tuberculosis; STI: Sexually Transmitted Infections; ART: Antiretroviral Treatment.

- **Participants on ART who prefer to remain within the Hlabisa HIV treatment and care programme**

Participants on ART who, after visiting the trial clinic, would prefer to remain within the Hlabisa HIV treatment and care programme will be asked permission for their records to be accessed by the trial nurse as per trial protocol (trial nurses will attend the nearest fixed clinic once a week). During home visits, all participants would be asked to give consent for their HIV records to be accessed by the trial team. Information required will be ART regimen, CD4 count and viral load. Linkage to existing records will be done using South African identification number and date of birth.

Adherence support

The importance of long-term adherence will be emphasised in the pre-treatment period during the treatment literacy classes, as per standard DoH procedures. In addition, at each trial site a peer support group will operate for the duration of the trial and all participants will be encouraged to actively participate.

Further, the trial clinics are positioned at sites no more than 45 minutes walking away, which for most people will be considerably closer than the DoH clinics. HIV-infected participants will also face a considerably shorter waiting time, in relatively pleasant surroundings.

6.4.5 Trial treatment modifications

Trial treatment modifications will be done according to national guidelines and developed in specific trial guidelines.

6.4.5.1 Occurrence of new pregnancies

Pregnancy testing (urinary hCG) will be offered at monthly follow-up to all women of childbearing age receiving trial drugs. In line with updated national ART guidelines, Atripla® will be used for women of childbearing age and during pregnancy irrespective of trimester of pregnancy (29).

6.4.5.2 Toxicity and treatment failure

In case of toxicity or intolerance, single drug substitutions will be allowed, as per South African guidelines (29, 33). The toxicities monitored will be renal dysfunction (TDF), liver function (NVP) and teratogenicity among pregnant women (EFV).

In case of treatment failure, switch to second-line therapy will be recommended (see appendix 15.1). VL and CD4 cell count will be performed 3 and 6 months after initiation and then 6-monthly for all participants on ART. The decision to switch to second-line ART will be taken on the basis of two consecutive HIV RNA measurements > 1 000 copies/ml at least two months apart (as per current South African national guidelines) (29, 33). Information from genotyping will also be made available in real-time to the trial clinician to guide the decision whether to switch regimens. The standard second-line regimen will be ZDV + 3TC + LPV/r.

Participants positive for HBsAg will continue TDF in their regimen should they need to switch to second-line therapy.

Trial participants fulfilling criteria for immunological and/or clinical failure in the absence of criteria for virological failure will be reviewed by the trial clinician before any switch in drug regimen. Second-line regimen will be prescribed following a

confirmed virological failure while the patient is on therapy, in accordance with South African guidelines.

6.4.5.3 Care of patients at the end of the trial

Trial participants receiving ART at the end of the trial will be transferred into the Hlabisa HIV Treatment and Care Programme and will remain on the same drugs. The drugs will then be provided by the KwaZulu-Natal Department of Health, whether first-line or second-line drug regimens (see Appendix 15.3).

6.4.5.4 Concomitant therapies

- Isoniazid preventive therapy (isoniazid 300 mg orally od) will be provided to HIV-infected participants in the intervention and control clusters as per national and provincial policies at the time of the trial (implementation in progress).
    - Co-trimoxazole (960 mg od) will be provided to HIV-infected participants in the intervention and control clusters if WHO stage II, III, or IV, or CD4 cell count < 200 cells/mm^3.
    - Multivitamins (1 tablet od) supplementation will be provided to HIV-infected participants on ART in the intervention and control clusters as per South African guidelines.

6.4.6 Sample handling and storage

Blood samples will be collected by trial field staff at the time of enrolment and by the nurse during subsequent visits to the trial clinics for HIV-infected trial participants according to the schedule presented table 1. The trial will utilise both the National Health Laboratory Service (NHLS) laboratory at Hlabisa Hospital and the Africa Centre laboratory at the University of KwaZulu-Natal, Nelson R. Mandela School of Medicine in Durban. Storage of samples will take place only at the Africa Centre laboratory. No samples will be transported out of South Africa without seeking appropriate permissions and approvals from the Ethics Committee, the University and South African regulators. Detailed procedures for sample collection, transport, processing, and storage will be included in a separate laboratory procedures manual/protocol.

The DBS samples collected during the HIV testing rounds will be transported daily via the sample processing unit at the Africa Centre to the Africa Centre laboratory in Durban. Two (2) of the DBS will be used on arrival at the laboratory for HIV testing. The remaining DBS will be stored at -80°C in the Africa Centre Virology Laboratory in Durban.

Blood will also be collected by venepuncture:

- at the initial clinic visit in all HIV-infected participants in the intervention clusters and in HIV-infected participants meeting ART initiation criteria in the control clusters
- at subsequent clinic visits for all HIV-infected participants on ART (M3, M6, M12, and 6-monthly up to M48) according to the schedule presented in Table 2.

A total of 30 ml will be drawn each time:

- 10 ml of blood will be sent to the NHLS laboratory for routine haematology, biochemistry, and HBV testing (these samples will not be stored)
- 20 ml of blood (2×10 ml tubes) will be sent to the Africa Centre laboratory for plasma viral load testing and storage at -80°C.

7. Trial outcomes and tools

7.1 Definition of trial outcomes

Table 5 summarises the definition of each trial outcome. If not explicitly mentioned, these outcomes will be documented in both phases of the trial, within the 10 clusters for the first phase and within 22 clusters during the overall trial.

Table 5
Definition of trial outcomes

Outcome	Definition, indicators	Comparison	Tool	See
**General population level**				
HIV incidence	⊙ Results of DBS, molecular epidemiology findings (clusters), primary resistance<br>⊙ Incidence estimates assessed by socio-demographic characteristics	IC vs.CC<br>*Second phase only*	IQ	7.2
Acceptability of initial HIV counselling and testing	⊙ HIV testing history at baseline<br>⊙ Attitudes towards HIV testing at baseline and changes over time<br>⊙ Different estimates of coverage/uptake of HIV testing per method of calculationSocial determinants of HIV testing uptake at individual and community levels	IC vs.CC<br>HIV+ vs. HIV-	IQ<br>IDI	7.3.1.1
Prevention practices	⊙ Circumcision uptake over time<br>⊙ Contraceptive use and pregnancies over time	IC vs.CC<br>HIV+ vs. HIV-	IQ	7.3.1.2
Sexual behaviours	⊙ Sexual partnerships patterns over time, disinhibition<br>⊙ Safe sex and condom use over time<br>⊙ Conjugal relationships (disclosure, unions, communication)	IC vs.CC<br>HIV+ vs. HIV-	IQ, SCS	7.3.1.2
Quality of life	⊙ Quality of life at baseline and over time	IC vs.CC<br>HIV+ vs. HIV-	IQ,<br>SCS,<br>CAP,<br>IDI	7.3.1.3
Household health care expenditures Treatment/care cost and cost-effectiveness	⊙ Cost analysis<br>⊙ Economic impact of HIV infection on the household welfare (health care use and health care expenditures)<br>⊙ Budget impact	IC vs.CC<br>HIV+ vs. HIV-	THR,<br>HHI,<br>IQ,<br>SCS,<br>CRF	7.3.1.4
Community awareness	⊙ Stigma toward PLWHA, perception of stigma<br>⊙ Perception about ART preventing HIV infection	IC vs.CC<br>HIV+ vs. HIV--	IQ,<br>SCS,<br>IDI	7.3.1.5

Outcome	Definition, indicators	Comparison	Tool	See
**HIV-infected participants**				
Acceptability / uptake entry into care	⊙ Expectations and perceptions of early treatment over time<br>⊙ Knowledge of HIV care and treatment<br>⊙ Time to initiation of treatment<br>⊙ Modalities of entry into care (health structures used)<br>⊙ Socio-cultural and economic determinants of entry into care	IC vs.CC	IQ, HHI, CRF, SCS, CAR	7.3.2.1
Therapeutic success	⊙ Patterns of retention and referral over time and their determinants	IC vs.CC	SCS, CRA	7.3.2.2
Programme retention	⊙ Patterns of retention and referral over time and their determinants	IC vs.CC	SCS, CRA	7.3.2.3
Morbidity, mortality and treatment	⊙ Mortality and severe morbidity (leading to hospitalization)<br>⊙ Tuberculosis (incidence)<br>⊙ Other morbidity events (infectious or not) by CD4 strata<br>⊙ Immunological and virological response<br>⊙ Socio-economic determinants of response to treatment<br>⊙ First-line treatment, durability, switches to second-line treatment, adverse events leading to drug discontinuation<br>⊙ Hepatitis B co-infection	IC vs.CC	CRF, SCS, IQ	7.3.2.4
Adherence to ART	⊙ Estimates, patterns and measurement of adherence over time<br>⊙ Determinants of adherence by CD4 strata	IC vs.CC	CRF, SCS, IDI, CAP	7.3.2.5
Acquired HIV drug resistance	⊙ Prevalence and incidence of acquired and transmitted drug resistance	IC vs.CC	CRF	0
Virological failure	⊙ Persistent VL > 1000 copies/ml	IC vs.CC	CRF	6.4.5.2
Toxicity	⊙ Monitoring of renal dysfunction (TDF), liver function (NVP), teratogenicity (EFV)	IC vs.CC	CRF	6.4.5.2
Vertically-acquired HIV infection	⊙ Uptake of PMTCT intervention(s) and relation with care program<br>⊙ Pregnancy outcomes with Efavirenz first-line ART and transmission<br>⊙ Overall HIV paediatric testing/treatment patterns in sample	IC vs.CC *Second phase only*	CRF	7.3.2.6

IC: Intervention cluster; CC: Control cluster;
HIV+: HIV-infected participants (treated or not); HIV-: HIV-negative participants;
THR: TasP Household Registration questionnaire; IQ: TasP Individual Questionnaire;
HHI: TasP Household Information Assets questionnaire; SCS: Social science Clinic-based Survey (SCB, SCC and SCI)
CRF: Case Report Form (CBC, CFU, CHE);
CAR: Clinic Activity Report; IDI: in-depth interviews; CAP: Consumer Advisory Panel.

Table 6 presents the outcomes that will be measured within the planned sub-studies if funded.

Table 6
Outcomes documented within sub-studies

Outcome	Definition, indicators	Comparison	Tool	See
**Health care system**				
Experience of care	⊙ Training and experience in HIV care, working conditions, practices and knowledge about ART management	IC vs.CC *Second phase only*	SCS, SS CAR	7.5.1
Characterisation of health facilities and activity	⊙ Type of health services, clientele, human resources, working times, technical equipment, HIV-care organization<br>⊙ Unit costs of the resources used	IC vs.CC	CAR, SS	7.4.3
Budget impact	⊙ Impact of TasP (level of coverage, rate of ART switches, drug prices) on health care system	IC vs.CC	CAR, THR, IQ, HHI, SCS	7.5.2

IC: Intervention cluster; CC: Control cluster;
HIV+: HIV-infected participants (treated or not); HIV-: HIV-negative participants;
THR: TasP Household Registration questionnaire; IQ: TasP Individual Questionnaire;
HHI: TasP Household Information Assets questionnaire; SCS: Social science Clinic-based Survey (SCB, SCC and SCI)
CAR: Clinic Activity Report; SS: Specific Survey.

7.2 Primary outcome

HIV incidence will be measured after multiple HIV testing periods of 24 months in 12 clusters, 36 months in 6 clusters and 48 months in 4 clusters depending on the person years of observation in individuals initially identified to be HIV negative.

7.2.1 HIV status measurement, using DBS with longitudinal follow-up

HIV testing will be longitudinally linked at the level of the participant and be conducted in six-monthly intervals. HIV testing will follow the standard South Africa DBS protocols used in the Africa Centre population-based HIV surveillance in past years (34). For the first phase of the trial, the duration of the three testing rounds will be six, then four and four months for the first 4 clusters and 6-monthly for the additional 6 clusters. For the second phase of the trial, four rounds of 6-monthly HIV testing will be conducted in all 10 phase 1 clusters and the additional 12 clusters in phase 2 over a period of 24 months.

Serologic status will be determined by antibody testing with a broad-based HIV-1/HIV-2 ELISA (Vironostika® HIV-1 Microelisa System (Biomérieux, Durham, NC, USA) followed by a confirmatory ELISA if the first test is positive (Wellcozyme HIV 1+2 GACELISA; Murex Diagnostics Benelux B.V., Breukelen, The Netherlands). In cases of discordant results a third ELISA test will be carried out.

7.2.2 A locally validated test for recent infection: capture BED enzyme-linked immunoassay (cBED assay)

For HIV-positive DBS a cBED assay will be performed during the first phase only to establish whether the HIV infection is recent (less than 6 months) or non-recent and confirm incidence estimates.

The addition of a test of recent infection to the standard longitudinal HIV testing serves three purposes, it will:

- Allow an immediate assessment of HIV incidence in the trial population at baseline (to confirm the assumptions made re sample size; without the need for a second longitudinal assessment).
- Improve the precision of the overall longitudinal measurement.
- Allow inclusion of individuals who refuse repeated testing but consent to testing once in the sample for incidence estimation and thus increase the power of the trial.

The cBED assay has been locally calibrated (35) and is thus an appropriate measure of HIV incidence at baseline for a community-randomized trial. Moreover, some of the calibration problems encountered in applications of the BED assay in other settings (36), become irrelevant in repeated application of the BED assay to estimate HIV incidence *differences* over time, since it is reasonable to assume that calibration parameters remain constant over time intervals of a few years.

While it would thus theoretically be possible to rely solely on BED assay testing in cross-sections of the population, which are not longitudinally linked at the individual level, such a measurement strategy does not seem advisable. The BED assay has not yet achieved acceptance as a routine approach to measure HIV incidence. Relying solely on the BED assay could thus reduce the perceived strength of evidence emanating from the TasP trial. However, we will use the cBED assay to identify people with likely recent seroconversion in the first round.

7.3 Secondary outcomes

7.3.1 Both within the general population level, stratified by HIV status, and among HIV-infected participants

7.3.1.1 Acceptability and uptake of HIV testing

Acceptability and uptake of HIV testing, current knowledge of HIV status, recent HIV testing history will be calculated for each successive rounds of home-based HIV testing and will be based on data collected during the home visit.

Acceptability of HIV testing will be estimated among different population subgroups, and will be defined among others as:

- the proportion of participants who are tested for HIV among all those eligible (individuals identified though the household registration)
- the proportion of participants who are tested for HIV among those not previously tested
- the proportion of participants who are tested HIV-negative at first round and who return to be tested once or more during the subsequent testing rounds. In addition, the uptake of HIV testing estimations will be refined using the data

from trial clinics and the DoH clinics on persons who attend for VCT. Participants from the trial sites who attend either service with their referral cards, given out during the rounds of home-based testing. Their data can be added into the total to provide estimates of testing coverage and the acceptability of different testing modalities. These outcomes will be calculated for successive rounds of testing. Essential socio-demographic data on non-responders (i.e. those declining HIV testing and the questionnaires and not subsequently attending either trial or DoH clinics for VCT) will be collected in each round and can in turn be linked in order to estimate an overall uptake rate over individual and over successive rounds of home-based HIV VCT. It will also be possible to estimate the appropriateness of the time intervals between testing rounds to maximise uptake.

Other proxy acceptability indicators can be calculated including uptake of HIV treatment and care among those testing HIV-positive either in the home-based HIV VCT or the local clinics (trial or DoH). We will also have individual level data on participants' HIV-testing histories, disclosure of HIV status, perceptions and expectations of treatment and collected at initial enrolment into treatment and care. It will be possible to calculate these estimates for each individual round of testing and cumulatively over the trial period.

7.3.1.2 Behavioural changes at individual level and sexual partnerships

Measurement of sexual partnerships, relationship status, prevention behaviours (e.g. condom use and HIV testing outside the trial), circumcision status, changes in contraceptive usage, attitudes towards PLWHA, perception and knowledge of HIV treatment, will be collected from all enumerated participants during the successive rounds of home-based testing. This will facilitate longitudinal as well as repeat cross-sectional analyses regardless of participants' self-reported HIV status.

7.3.1.3 Quality of life

Quality of life will be explored:

- At cluster-level, among all participants responding to the *IQ questionnaires* during the first and last rounds of home-based HIV testing during the first phase. The EQ-5D scale will be used for this purpose since this instrument is a short generic health instrument which can be administered to both HIV-infected people and HIV-negative people or people of unknown HIV status.
- Among all HIV-infected participants attending the study clinics. These measurements are planned for at W0 (i.e. date of entry into care for treatment ineligible patients and any of the pre-treatment visits for ART-eligible participants), M6, M12, and 6-monthly up to M48 (see Table 2, Table 3 and Table 4). The quality of life module will be designed using standardised validated instruments appropriate to the context and culture of the participant population:
    - the *Patient Reported Outcomes Quality Of Life specific to HIV* (PROQOL-HIV): this instrument comprises 43 items (39 items for health-related quality of life and 4 individual items) and takes into account a comprehensive set of dimensions related to the quality of life in PLWHA such as sleep, treatment's perception and treatment management, perceived HIV-symptoms and impact of treatment's side effects, which are not (or not adequately) taken into account in other HIV instruments like the WHOQOL-HIV (37). Its psychometric

properties have been evaluated and validated in different context including Sub-Saharan Africa and the instrument has been shown to be sensitive to differences in culture, gender and ethnicity (38). As the PROQOL-HIV has not been validated as an isiZulu language instrument, a short linguistic validation study could be implemented (to be described at a later stage)

- the *HIV/AIDS stigma instrument for PLWHA* (HASI-P): this tool assesses stigma perceived in PLWHA, which is one of the dimensions of the quality of life. This instrument has been built to measure perceived stigma by PLWHA in Southern African countries and its psychometric properties have been validated in IsiZulu language (39). This face-to-face questionnaire includes 33 items regarding the occurrence of different events, which may have happen because of the HIV status (e.g. ask to not touch child, blamed for HIV status, called bad name) as well as thoughts or feelings.

7.3.1.4 Cost-effectiveness performance

A cost-effectiveness analysis (CEA) will be carried out based on data from the TasP trial and will be completed with a mathematical (Markov) model to estimate the effectiveness and costs of the strategies compared over time, i.e. comparing treatment initiation as soon as HIV infection is diagnosed versus delayed treatment initiation according to WHO recommendations.

The main outcomes of this CEA will be the number of life years gained and also the number of Quality Adjusted Life Years (QALYs) saved.

A generic health instrument is required to determine different healthcare states which can be classified according to preferences. We will use the EQ-5D scale, which has been widely used in the literature to conduct cost-utility evaluation, both in Northern and in Southern countries, including South Africa (40, 41). This instrument has been found to discriminate between different health states amongst PLWHA and has been validated in isiZulu formats (42, 43).

Both direct costs, as well as indirect costs will be evaluated both at the cluster level and among HIV-infected participants. The questions for the CEA will be integrated into the *IQ* at the cluster level within the *CRF* and *the clinic-based questionnaires* to be administered to HIV-infected participants. The following costs will be collected throughout the trial period at different time periods:

- At the cluster level:
    - Direct costs: travel costs and out-of-pockets expenditures for health;
    - Indirect costs: income loss due to the illness (human capital approach, based on the estimation of patients' and family income loss due to illness and due to health care utilization);
- Among HIV-infected participants:
    - Direct costs documented using the CRF (ART prescribed, laboratory tests, physician visits, hospitalizations, and concomitant treatments) and CAR (human resources dedicated to HIV-care, consultation time, costs of laboratory tests...);
    - Direct costs within the clinic-based questionnaires: travel costs, out-of-pockets healthcare expenditures;

- Indirect costs: income loss due to the illness documented at baseline (date of entry into care), at treatment initiation, and during follow-up for HIV-infected people (see Table 2, Table 3 and Table 4).

7.3.1.5 Community awareness, attitudes and behaviours

Community awareness, attitudes and behaviours will be extensively studied in the first phase. These are measured at the individual level in the *IQ questionnaires* completed during each successive round of home-based testing. Other global indicators will be derived from participation rates in successive rounds of home-based testing. The extent to which the same methodology or a more complex one will be used in the second phase will be decided at the end of the first phase.

7.3.1.6 Societal response

Societal response is a composite outcome, which will be assessed during the second phase of the trial only, using a variety of methods and indicators:

- changes in attitudes regarding testing, treatment and persons living with HIV measured during home surveys;
- changes in individuals community, economic and social participation (employment, living in couple, parenthood,) and experience of people living with HIV (stigma and discrimination) surveyed at trial clinics using the social science clinic-based questionnaires and explored within in-depth interviews of HIV+ individuals on treatment;
- perception and analysis of the social impact of the programme in panels of people in different subgroups (young adults, male and female adults separately, HIV+ individuals, key informants of social leaders and traditional healers)

With these various outcomes we will be able to compile an extensive array of measures that can be combined and compared between clusters and between individuals (HIV-infected and non-infected). This will in turn be supplemented with qualitative data, which will provide a nuance image of the overall social impact of the programme and the trial.

7.3.2 Only among HIV-infected participants

7.3.2.1 Acceptability and uptake of entry in care and treatment

Using the patient identifiers and mechanisms already described we will be able to monitor acceptability and uptake of treatment at several levels.

At the individual level we will be able to estimate the time from receipt of HIV test result to enrolment into treatment at the trial clinics or DoH fixed clinics (if any participant chooses that option). We will be able to monitor the transfer of care of patients currently being monitored (as yet ineligible for ART) in the fixed clinics to the trial clinics. The employment of a tracker will ensure that people eligible for treatment in the trial clinics who do not attend, and those that subsequently default from care, are followed-up, are appropriately assisted to access the trial clinics, if necessary, and that they are not otherwise lost to follow-up.

In addition to measurement of these key public health outcomes, with the data collected at treatment enrolment it will be possible to characterise in detail the population entering into treatment and care and to estimate social factors that facilitate entry (e.g.

household composition, social support, disclosure to family, treatment knowledge). All of these intermediate measures will be useful in determining the need and content of any special interventions to support treatment uptake in the main trial.

7.3.2.2 Therapeutic success/evaluation of ART

This will be estimated as the proportion of HIV-infected participants who have undetectable VL after six months of ART.

7.3.2.3 Programme retention

Retention in care will be assessed at 12 months, 24 months and 36 months post-enrolment. Retention will be defined as those still under active follow-up in the trial. Loss to follow-up on ART will be defined as ≥ 3 consecutive missed appointments. For those not eligible for ART, loss to follow-up in control clusters will be defined as > 9 months from last clinic visit and/or CD4 cell count.

7.3.2.4 Mortality, severe morbidity, and tuberculosis

Secondary endpoints to be compared between the intervention and control clusters include all-cause mortality, HIV-related mortality, WHO stage IV disease, serious non-AIDS events, and tuberculosis. Serious non-AIDS events include cardiovascular disease (stroke, myocardial infarction), end-stage renal disease, decompensated liver disease, and non-AIDS-defining cancers. Tuberculosis includes both pulmonary and extra-pulmonary disease, either with or without microbiological confirmation.

7.3.2.5 Adherence

Adherence will be measured every 4 to 8 weeks depending on frequency of clinic attendance for patients on ART using a scale that has already been translated and validated by Africa Centre researchers with patients from the Hlabisa Treatment and Care Programme where previous analysis has focused on consistency of reported non-adherence (44). This tool, using visual analogue scale, pill identification test and pill count, will be included in the CFU questionnaires.

However, as non-adherence was rarely reported and as the questions used performed poorly in identifying patients with treatment failure (44), we will test an additional scale constructed to limit both recall and social desirability bias and which has been tested in different settings (45-47). This tool includes several questions related to dose taking during the previous 4 days and the respect of the dosing time schedule during the previous 4 weeks. Adherence scores which are computed using a validated algorithm allowing to classify patients into highly adherent, moderately adherent and lowly adherent have been found to be significantly associated with viral load (46). Another item focusing on the occurrence of treatment interruptions lasting more than 2 consecutive days during the previous 4 weeks was found to be a predictor of resistance development (48) and has already been tested in another context (45). This second measure of ART adherence will be surveyed every 6 months (i.e. at M6, M12, and 6-monthly up to M48) and included in the *Social science clinic-based interviewer-administered questionnaire* (SCI).

7.3.2.6 Paediatric HIV infection

The sub-district has a well-functioning PMTCT programme, which includes HIV DNA testing (PCR on a DBS) of infants at six weeks of age, repeated one month after

cessation of all breastfeeding. A PMTCT database has been developed in the sub-district, charting the progress of pregnant women from the time of antenatal booking through to infant HIV testing after cessation of all breastfeeding. It has been shown in this programme that ART introduction in mothers and infants may already have had more impact on early life mortality than PMTCT itself (49).

In the trial intervention clusters all HIV-infected women will be initiated on ART irrespective of the CD4 count or the clinical stage. In the control clusters, women who are not yet eligible for HAART (i.e CD4>350 cells/μL) will be managed according to the best available PMTCT guidelines approved by the South African national authorities (Currently WHO option B) (29). This involves the prescription of FDC triple therapy (TDF+FTC+EFV) to pregnant women and breastfeeding mothers with CD4>350 until one week after the complete cessation of breastfeeding. This is adapted in certain situations such as the presence of active psychiatric illness or renal disease. Women receiving TDF/FTC/EFV for prophylaxis rather than for their own health will undergo, WHO clinical staging and hepatitis B testing prior to discontinuation of ART. If a woman treated with TDF/FTC/EFV has stage 3 or 4 disease or evidence of hepatitis B infection (hepatitis B surface antigen positive) then the regimen will not be stopped after the cessation of breastfeeding but will continue lifelong. CD4 count will be done 6 months after the cessation of breastfeeding or if the last CD4 count was done more than 12 months ago.

Women in both the intervention and control clusters will be asked about pregnancy at each of their routine visits; if pregnant, data will be collected as part of the trial including date of delivery, mode of delivery, stillbirth, premature termination, infant outcome, and infant DNA status at 6 weeks and after cessation of all breastfeeding if known, as antenatal care, delivery and post-natal care are managed in the DoH clinics.

7.3.2.7 HIV drug resistance

HIV-1 genotypic resistance testing will be performed for:

- HIV infected individual initiating ARV and identified with virological failure during the trial. The virological failure is defined as a viral load > 1 000 copies/ml on two separate occasions two months apart. The HIV-1 genotypic resistance will be performed on paired samples from the same participant: one from time of virological failure and second from pre-treatment if available
- HIV infected individual already on ARV at trial entry and identified with virological failure at enrolment
- all participants who seroconvert within the trial

The HIV-1 genotypic resistance test will be done at the Africa Centre laboratory utilising stored plasma samples of HIV infected individual receiving ART and stored DBS. Procedures will follow an existing and internationally validated protocol established for an on-going clinical study of ART failure in the HIV Treatment & Care Programme. Results will be returned to the trial clinician within 14 days of sample collection. Genotyping for all participants who seroconvert within the trial - these results will not be available to clinicians in real-time.

The pre-treatment samples will be used to assess the surveillance of transmitted drug resistance during the trial whereas virological failure genotypes will be used to estimate the level of developed resistance mutations. Sequences will be produced using the ANRS guidelines for genotyping and analysed using drug resistance algorithms, such as the Stanford HIVdb, ANRS and Rega algorithms.

Further, given the short time of the trial and potency of the ARV drugs to be used in the trial, in a sub-study (n~10) we will also consider genotyping at the DNA level of participants with undetectable VL while on ART to assess development of resistance at low viral replication levels and sequencing minority populations using either direct PCR or pyrosequencing methods in order to study the link between minority populations and clinical outcome.

7.3.2.8 Molecular epidemiology

We will analyse all sequences using a phylodynamics framework. Phylodynamics can be used in order to estimate the date of origin of epidemiologically important events such as the introduction of a new viral strain in a geographical area and identification of transmission networks between intervention and control clusters.

7.4 Data collection tools

A number of detailed standard operating procedures (SOPs) will be developed for the counsellors involved in the home-based testing and surveillance, the trial clinic nurses and counsellors, the physicians, the pharmacy and the laboratory technicians. A combination of quantitative and qualitative trial instruments will be used to assess key intermediate process, behavioural and outcome indicators in the trial.

7.4.1 Household and individual home-based questionnaires

Throughout the trial (first and second phase), in each round of home-based HIV testing, we will collect basic information using home-based questionnaires at household and individual level.

In the first round, the *TasP household registration questionnaire* (THR) will be administered to the head of household to document household-level social and demographic characteristics: number and age of residents. Housing characteristics (water, electricity), household income, food security among others will be documented using the *TasP household information assets questionnaire* (HHI). These household questionnaires will be repeated to allow for the updating of information about household members at each round in order to document changes in household.

During the first phase of the trial, the *TasP home-based individual questionnaires* (IQ1 to IQ3) will be composed of a core questions repeated at each round and a set additional questions specific to certain rounds: individual-level social and demographic characteristics, HIV testing behaviour, sexual behaviour, partnership and sexual network patterns, attitudes and beliefs about HIV infection, HIV testing and treatment, stigma and disclosure, healthcare use and healthcare expenditures and changes within the households, and quality of life (see Table 7).

In phase 2 of the trial, identical *home-based individual questionnaires* designated IQ will be administered during the six-monthly rounds of HIV testing. The phase 2 IQ will address all components listed in Table 7.

Table 7
Data collection during the HIV testing rounds, among the general population: Household and individual questionnaires (first phase of the trial)

	IQ1	IQ2	IQ3
**Home-based Household questionnaire**			
Household head verbal consent	×	×	×
Household composition and socio-economic characteristics	×		
Changes in household composition (including in-out migrations/mortality/newly eligible)		×	×
**Home-based Individual questionnaire**			
Individual consent for questionnaire	×	×	×
Knowledge/beliefs about HIV infection	×		×
Knowledge/expectations about treatment	×	×	×
HIV testing history	×		
HIV testing attitudes/beliefs	×		×
Uptake of testing opportunities	×	×	×
Self-reported knowledge of HIV status	×	×	×
Disclosure of HIV status	×		×
Sexual partnerships	×		×
Condom use	×		×
Contraceptive use	×		×
Circumcision status	×		×
Risk behaviours (alcohol etc.)		×	×
Quality of life	×		×
Stigma towards PLWHA		×	
Health care use		×	×
Health care expenditure		×	
**Home-based HIV testing**			
Individual consent for DBS and/or HIV testing	×	×	×
DBS	×	×	×
Home HIV counselling and testing (rapid test)	×	×	×

7.4.2 Social science Clinic-based survey

The clinic-based survey exploring HIV-infected participants' behaviours and socio-economic status will be composed of different sets of questions administered repeatedly every six months or specifically at baseline (i.e. date of entry into care for non-eligible patients and date of any of the pre-treatment visits for ART-eligible patients), as presented in Table 3. The topics explored will be: testing experience (baseline only), ART knowledge, ART perception and decision, disclosure, sexual behaviour, self-reported ART adherence, HIV Quality of Life, stigma and discrimination, social and community support, economic and health expenditures, satisfaction with care.

The clinic-based survey will be divided in three questionnaires:

- the *Social science Clinic-based Baseline questionnaire* (SCB), administered by the ART counsellor at baseline;

- the *Social science Clinic-based Counsellor-administered questionnaire* (SCC) on non-sensitive questions such as disclosure or health expenditures, administered each six months of follow-up and
- the *Social science Clinic-based independent Interviewer-administered questionnaire* (SCI) with sensitive questions (which are subject to social pressure and thus to social desirability bias) such as sexual relations or adherence, administered each six months of follow-up.

7.4.3 Case Report Forms (CRF)

The case report forms will be completed by the counsellors and the research nurses in the trial clinics. There will be baseline (CBC) and follow up (CFU) CRFs to be completed by the counsellors and a combined history and clinical examination (CHE) CRF to be completed by the research nurses. The same CRFs will be completed for all participants irrespective of their ART status at enrolment into the trial (See Table 5).

7.4.4 Clinic Activity Reports (CAR)

Throughout the trial, each trial clinic will keep detailed monthly activity records. In addition in phase 2 we will attempt to audit patient waiting times, staffing levels, stock outage, the adequacy of trial logistics and support using tools developed for the task.

7.4.5 Qualitative data

In the first phase only, two qualitative studies are planned to address acceptability of repeat testing, adherence and quality of life among those starting treatment in the trial clinics.

In the first of these we will convene a **consumer advisory panel** (CAP) in each of the four clusters. This panel will work with the social science team and the trial's community liaison officer to provide an on-going monitoring of the community experiences of the trial, perceptions and understanding of the intervention and early advice on any possible problems. The members of each panel will also serve as key informants and expert advisors to help the trial team ensure that community entry, awareness and education plans are fully developed for the second phase of the trial. The four community consumer panels will be conveyed according to a purposive sampling framework. Africa Centre researchers are currently using this methodology to develop and deliver youth HIV prevention interventions. In this instance we will assemble and engage repeatedly with the four purposively selected groups on a bi-monthly basis to discuss specific issues, which we believe the panel members, shared in common. Our community consumer panels will comprise four purposively selected groups from among the different communities (two members from each community). The four panels will be comprised of women, men, elderly, health care workers, and traditional authorities. Each panel will meet on six separate occasions with the same facilitator. All sessions will be video-recorded with participants' permission. Panel meetings will adopt a combination of different approaches including conventional focus group discussions and participatory action methods including joint problem solving and assumption of expert advisor roles.

The second small qualitative study will use repeat **in-depth interviews** (IDI, n=15) to explore the adherence and quality of life in HIV-positive people initiating treatment in the trial clinics. This is a unique opportunity to examine adherence and quality of life qualitatively, and in the context of people who would not normally be receiving treatment. It also offers an important opportunity to explore understandings of the

importance of adherence in the context of both personal health benefits and HIV prevention.

7.5 Sub-studies

7.5.1 Survey of health care professionals

The implementation of universal testing and treat immediately intervention will represent major challenges for the health care system and especially for human resources which are one of the key factors for the success of the intervention. Sub-Saharan Africa countries currently encounter lack of qualified health care professionals, unequal geographical distribution of health resources, generalized low wages, difficult working conditions and lack of carrier development (50, 51). Such factors have been shown to have an impact on human performance and may jeopardize quality of care, as well as HIV treatment delivery (52).

To identify potential obstacles to the implementation of TasP in HIV care, treatment and prevention services and to identify changes needed to improve the adherence to TasP principles by service delivery personnel, a quantitative survey will be conducted among health care providers in charge of PLWHAs and working in the facilities included in the TasP trial, both the trial clinics and the DoH fixed clinics. Data will be collected using a quantitative survey instrument previously used in a research programme about HIV care among medical professionals in Cameroon (53). Data collected will include information on socio-demographic characteristics, training and experience in HIV care, working conditions, practices and knowledge about HIV and ART management, opinions about the TasP intervention. This survey will be carried out once separate from the routine care and in the second phase of the trial.

In addition, data relating to the characteristics of the health care facilities where TasP will take place, such as types of health services, size of HIV clientele, number of ART-treated patients, human resources in charge of HIV care, working time devoted to the care of PLWHAs and staff compensation to estimate the cost of human resources involved in patient care, shall be obtained through access to institutional reports, computer systems and interviews of each health centre’s managers and staff. This second survey will be carried out at baseline and at 12-month, both in the second phase of the trial.

7.5.2 Budget impact analysis

A budget impact analysis (BIA) will be planned in the first and second phase of the trial to examine the potential financial impact, at macro level, that is on national, regional or local health budgets, of the introduction of the TasP strategy into the healthcare system. This approach will be a complement of the CEA but does not include additional data collection. The impact on the total costs will be estimated. A variety of sensitivity analyses will be carried out varying for example the level of coverage, the rate of switching to second-line regimens, or drug prices.

8. Methodological and statistical considerations

8.1 Cluster selection

The randomisation units within the TasP trial will be clusters within Hlabisa sub-district outside of the DSA (see Figure 1). Randomisation will occur at the start of the first phase.

The area consists of 150 local areas (neighbourhoods). These were aggregated into 34 clusters of between one and six contiguous neighbourhoods, each cluster comprising an average of 1 000 individuals >15 years of age. Clusters were designed to encompass social networks based on earlier studies in the DSA (54), to keep the potential for cross-arm contamination to a minimum. To further reduce the potential for contamination, we ensured that all resulting clusters were of significant geographical size (>15km^2 in area). This meant that the number of individuals in peri-urban clusters were larger than their rural counterparts.

Adjacent communities representing relatively distinct social and sexual networks[1] were grouped together to form the units of randomization (median population ≥ 15 = 1 000; median area = 19 km^2). From a statistical efficiency perspective (least sample size) more clusters of fewer people should be used. However, having very small units of randomization would result in a large potential for contamination. With this trade-off between statistical efficiency and contamination potential in mind we combined adjacent neighbourhoods together to form relatively large randomization units (with similar numbers of participants) with a distinct social identity. This considerably reduces the risk of contamination whilst still retaining a relatively large number of clusters for the trial (56).

To further evaluate the potential impact of contamination of the results on the outcome of the trial we have run extensive geographical simulations of possible randomization scenarios. The results of these analyses demonstrate that chance groupings of neighbouring communities randomized to the same arm of the trial result in 'super-clusters' with a median of aprooximately 4 000 participants ≥ 15 years of age and median size of 48 km^2. Empirical data from a sexual behaviour survey conducted in 2005 showed that 55% of men interviewed reported that their most recent partner in the past year lived in the same immediate *izigodi* (a Zulu term for an administrative area headed by a local chief), which have a median area of 16.9 km^2. Extrapolating from these results, we are expect that only a very small proportion of trial participants will have sexual partners outside these 'super-clusters' (median size=48 km^2). The exceptions to this rule will be people residing near the borders of these 'super-clusters' and the chance occurrence of singleton communities in the randomization.

We considered trying and further reducing the small amount of inter-arm contamination by the designation of a series of 'buffer' communities around each community used in the trial. However, this was rejected for two reasons: a) It would have the undesirable effect of reducing the size of the 'super-clusters' and hence increase the chance of having partners outside the area to which the participant was randomized and b) It would increase the risk of an HIV-negative participant in an intervention community of having an HIV-positive untreated partner in the

[1] The 312 neighbourhoods in the sub-district were demarcated by visiting each of the 26 000 homesteads in the sub-district and asking a key respondent to name the local area (neighbourhood) where they lived. From this data, a GIS methodology was used to digitize local areas around clusters of responses to the local areas question (55).

neighbouring buffer communities (because the level of ART coverage in buffer communities would be even lower than control communities where ART coverage will increase through increased access to VCT services delivered as part of the trial).

8.2 Modelling exercise and sample size calculation

8.2.1 Principles of the model

We used STDSIM to provide estimates of the potential impact of the TasP trial. STDSIM is a well-established stochastic microsimulation model of the spread and control of HIV (57-71). The model that simulates the life course of individuals in a dynamic network of sexual contacts and consists of four modules: demography, sexual behaviour, transmission and natural history, and interventions. The demography module covers the processes of birth, death, and migration. Processes for initiation and dissolution of sexual relationships, mixing according to age preference, sexual contacts within relationships, and sexual contacts between female sex workers and their male clients are defined in the sexual behaviour module. In the transmission and natural history module, transmission probabilities per sexual contact are specified for HIV and 5 other STDs. The interventions module specifies the timing and effectiveness of control measures in curbing transmission (e.g. condom use) or enhancing survival (e.g. antiretroviral therapy). STDSIM has been extensively used to evaluate behavioural interventions (60-62, 72), syndromic treatment for STIs (63, 69), male circumcision (70), explain different HIV epidemics in sub-Saharan Africa (64), and, more recently, the impact of ART on HIV epidemics (57-59, 73). For the impact estimates of the TasP trial we used the previously published quantification for rural KwaZulu-Natal, South Africa. The model was built using data from the Africa Centre and accurately captures demographic, behavioural, and epidemiological characteristics of the local epidemic (58, 59, 73).

8.2.2 Model input parameters

Table 8 gives an overview of the main parameters and assumptions related to the trial impact estimates. We simulated an intervention of bi-annual screening of the population aged 15 and over, and compared the impact of immediate ART for all HIV infected individuals (treatment-arm) with ART only for those with CD4 cell counts of ≤350 cells/μL. We ran the model separately for all clusters in order to capture the phased roll-out design of the trial. We assumed the intervention to be rolled-out in a total of 22 clusters: four clusters start in 2012, six in 2013, and a further 12 in 2014. We examined the impact on incidence rates, cumulative incidence, and ART coverage up to end-2016. The parameter settings used are those that gave a 30% reduction in cumulative incidence.

Table 8
Parameter settings and characteristics of HIV epidemic as modelled by the STDSIM model.

Parameters and baseline epidemic characteristics	Assumptions
Reduction in infectiousness due to ART	90%
Proportion having partners in clusters in opposite arm of the trial	10%
HIV testing uptake (both arms)	
HIV test offer among those registered	90%
Test acceptance among those offered	80%
Linkage to care upon diagnosis among those accepting the test	70%
HIV prevalence in end-2011 (population aged 15+)	24%
HIV incidence in 2011 (population aged 15+)	2.4/100 PY
Proportion of all HIV infected people on ART in end-2011	39% (64% of those eligible at ≤350 cells/μL)

Note: Model was quantified to represent the HIV epidemic in rural KwaZulu-Natal, South Africa, using demographic, behavioural, and epidemiological data from the Africa Centre. Model and quantification described in detail elsewhere (58, 59, 73).

Our model predictions give an estimated reduction of cumulative incidence in end-2016 of 31.7%, or 28.8% by end-2015 (Table 9). Incidence rates in the control arm would decline from 2.4/100 PY in 2012 to 1.4/100 PY at the end of 2016. In the intervention arm, incidence would decline from 2.4/100 PY to 0.7/100 PY over the same period (see Figure 5). Mid-2015 we will be in a strong position of conduct an interim analysis to show whether a significant reduction in incidence could be detected either at the end of 2015 or 2016. The details of this analysis are described in detail below.

Table 9
Projected 5-year impact of cluster-randomized trial of treatment as prevention on HIV incidence and ART coverage in rural KwaZulu-Natal, South Africa.

	Early-2012	Mid-2014	End-2015	End-2016
Incidence rate (per 100 PY)				
Control-arm	2.4	1.7	1.4	1.4
Treatment-arm	2.4	1.3	0.8	0.7
Average incidence over 5 year period (per 100 PY)				
Control-arm	n/a	n/a	n/a	1.7
Treatment-arm	n/a	n/a	n/a	1.2
Cumulative incidence reduction				
Treatment-arm versus control-arm	n/a	22.9%	28.8%	31.7%
Proportion of HIV infected people on treatment				
Control-arm	39%	59.4%	64.5%	66.5%
Treatment-arm	39%	81.6%	89.7%	90.8%

Note: Population is screened bi-annually. All HIV infected people in the treatment-arm are offered immediate ART, and HIV infected people in the control arm are offered ART when their CD4 cell count is below 350 cells/μL.

Figure 5
Projected 5-year impact of cluster-randomized trial of treatment as prevention on HIV incidence rates in rural KwaZulu-Natal, South Africa.

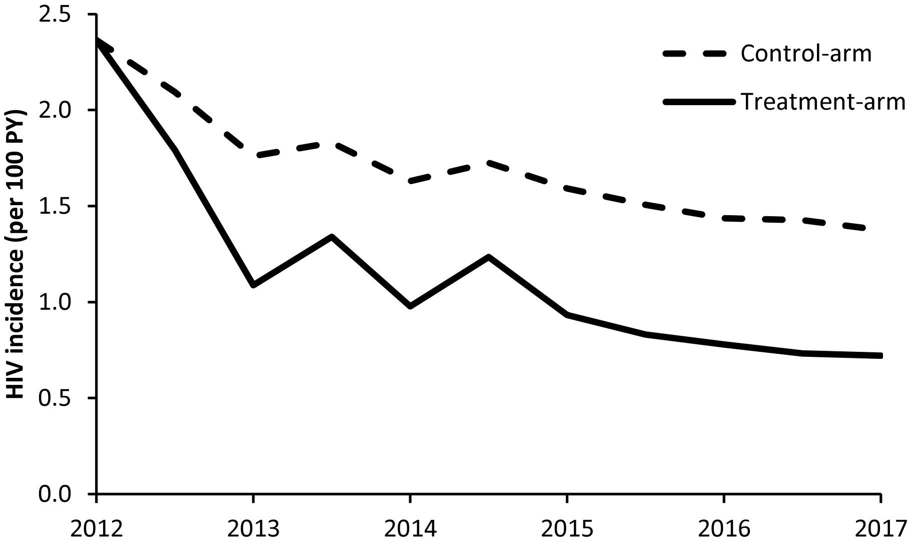

Note: Population is screened bi-annually. All HIV infected people in the treatment-arm are offered immediate ART, and HIV infected people in the control arm are offered ART when their CD4 cell count is below 350 cells/μL. Spikes in incidence are explained by the enrolment of additional clusters (6 in 2013, 12 in 2014).

8.2.3 Sample size calculation based on model assumptions

The initial design proposed a cluster-randomised trial with 2x17 clusters, covering a total population of 34,000 adults aged 16 years and above followed over two years. However, a phased approach of trial implementation was adopted as feasibility and acceptability were deemed critical for the overall evaluation of the intervention. In this design at the end of 2015, four clusters would be followed up for four years, six clusters for three years and a further 12 clusters for two years. This would give a total of 22 clusters, 58 cluster-years of follow up for a total trial size of 22,000.

- **Primary Outcome**

Our sample size calculations are based on an overall 34% reduction in cumulative HIV incidence in HIV-negative participants over 58 cluster-years of observation (see Table 10). To have sufficient numbers to be able to demonstrate this difference, our sample size calculations indicate that 22 clusters (11 in each arm), with 1,000 participants >15 years of age in each cluster (N=22,000; 17,600 HIV-negative), are required to achieve this objective. Underlying statistical parameters are as follows: a 80% power to detect this difference, an alpha-type-one error of 5% (two-tailed) and an allowance of 20% of study participants lost to follow up. We assume also a coefficient of variation of 0.25 to account for within-group variation between clusters.

Table 10
Estimated power to show a 34% reduction in cumulative incidence using the Satterthwaite approximate F test for two-sample comparison of proportions with clustering for 22 clusters (2012-2015).

Reduction in cumulative HIV incidence	34% end 2015<br>(1.49 vs 2.25%/year)
CV	0.25
Power	80%
Number of Clusters	22
Cluster Size	1 000
Total number of HIV negative adults	17 600
Total number of cluster-years	58<br>*4 for 4 yrs*<br>*6 for 3 yrs*<br>*12 for 2 yrs*

In the control arm we expect an average HIV incidence of 2.25 per 100 person-years over the course of the trial (this is 17% lower than the incidence currently observed in the Africa Centre DSA and recognises that incidence will likely decline in the overall population as a result of public sector roll-out of ART according to South African guidelines).

In the intervention arm we expect an average HIV incidence that is 34% lower than the control arm i.e. 1.485 per 100 person-years.

To take into account the different follow up times in the different groups of clusters opened at different times, we based our calculations simply on the proportion sero-converting in the intervention versus control clusters over the course of the trial. This approach will yield appropriately conservative sample size estimates.

In each cluster we expect to register 800 HIV-negative individuals of whom we expect to be able to successfully follow up 640. The average follow-up time for each individual is derived by subtracting half a year from the follow-up time in the cluster in which they reside. So for example, in the two control clusters that are followed up from 2012 to 2015, the total person-years of follow up = 640*2*3.5 years =4,480 which at an annual incidence of 2.25 per 100 person-years would equate to 100.8 sero-conversions observed by 2015.

Overall, the total person-years of follow up in each arm of the trial will be an estimated 15,040 (and 30, 080 person-years of observation in the whole trial). Thus, at an annual incidence of 2.25%, 4.8% of those observed to be HIV-negative at baseline would have been observed to seroconvert in the 11 control clusters by the end of 2015 that is 338 new HIV infected. The corresponding figure for the 11 intervention clusters would be 3.1725% for around 223 new HIV infected. Therefore, the number of HIV infected patients in the 11 intervention clusters will be 2098 (338+1760) and 1983 in the 11 control clusters (see Table 11). (Note that this method, using average follow up time provides very similar total person-years as in the approach accounting for different follow up times in different clusters.)

Table 11
Participants at risk of sero-conversion and expected number of incident cases

	Expected annual HIV incidence	Total	Nb of HIV+ at T0	Nb of HIV+ followed	Nb HIV- at T0	Nb HIV- followed*	Total HIV- person-years	Nb new HIV+ by end 2015
Cont	2.25%	11,000	2,200	1760	8,800	7,040	15,040	338
Interv	1.485%	11,000	2,200	1760	8,800	7,040	15,040	223

* assuming 20% of loss to follow-up

- **Secondary outcomes**

Within the overall trial, two kinds of secondary objectives are distinguished: those measured on the total population (acceptability and uptake of HIV testing, behavioural changes…) and those measured in HIV-infected patients only (morbidity and mortality, virological response and resistance). We describe below some of our expectations regarding the latter.

Mortality rates in HIV-infected patients

Assuming a 9 per 100 person-years rate of early mortality in the control clusters based on published data from the Hlabisa HIV treatment and care programme (32) for the post-2011 CD4 350 eligibility threshold, and assuming that the median duration of follow-up while being infected is two years for prevalent cases and one year for incident cases, HIV infected patients will contribute 3858 person-years in the 11 control clusters and 3743 person-years in the 11 intervention clusters. Following these assumptions, we will be able to show a reduction of at least 33% (incidence of 6 per 100 py) in the intervention clusters.

If we were to use only data from phase 1 on the 5+5 clusters, then we would be able to detect a reduction of 50% in the rate of early mortality (from 9 to 4.5 per 100 person-years of follow up) only.

Proportions in HIV-infected patients (adherence, virological success, resistance)

Assuming about 182 HIV-infected patients per cluster (around 4000 infected patients in total) we will be able to show, with a statistical power of 80%, at least a 17% difference for characteristics with a prevalence of 60% or 22% for a prevalence of 80% or 25% for a prevalence of 90% (that is for example, an adherence of 90% in control vs. 65% in intervention clusters).

If we were to use only data from phase 1 on 5+5 clusters, then we would be able to detect a difference in the proportions by cluster arm in a variable that has a 90% prevalence of 37% (compared to the 25% noted above for the 22 clusters).

Calculations were performed according to Hayes & Bennett formulas for rates and proportions assuming 20% of loss to follow-up, 5% type I error.

8.3 Randomization

Randomisation was performed by the trial statisticians (F. Tanser, R. Thiebaut) at the start of the trial for all 34 clusters. Random numbers were generated for all 34 communities in the trial area using MapInfo 10.0. Communities were randomly allocated in equal measure to control and intervention communities (17:17). To minimise the degree of between-cluster variation, communities were stratified on the

basis of predicted HIV prevalence. Randomisation was carried out within each stratum to derive an equal number of control and intervention communities per stratum.

8.4 Plan of analysis

8.4.1 Criteria for continuation/discontinuation at the end of the first phase

Given our proposed phased approach, interim analyses will be conducted at three possible time-points over the course of the trial. As well as analysing levels of uptake of HIV testing and coverage of treatment, we will analyse differences in cumulative HIV incidence between intervention and control communities at the end of 2013 (≈≈19% of total person-time of observation accrued) and 2014 (≈≈53% of total person-time of observation accrued). Our modelling results demonstrate that any initial reduction in HIV incidence is likely to be relatively modest given the expected non-linear decline in incidence overtime (74). However, the trial will be discontinued if the interim analyses provide clear evidence of sexual disinhibition or reduced treatment adherence in the intervention clusters relative to the control clusters, that is, that the hazard ratio of acquiring new infection (intervention versus control) is >1.0 ($p<0.05$). In 2014 we will conduct a predictive power analysis to quantify the likelihood of observing a significant reduction in incidence if any observed reduction is sustained to the end of 2015. The predictive power probabilities will be calculated using the available information and several prior distributions. Futility will be concluded if the predictive power probabilities are < 0.2 or efficacy if > 0.8.

8.4.1.1 Feasibility

Clear indication that given the parameters measured during the first phase (HIV prevalence in the clusters and assessment of incidence at baseline on the basis of cBED, initial HIV testing uptake, repeated HIV testing uptake, ART treatment uptake, migration and the extent of sexual partnerships with people outside the trial setting) the TasP trial will lack the statistical power to detect significant HIV incidence differences between the intervention and control communities. Such observations will lead to the conclusion that the trial is not feasible.

The criteria used to decide on continuation of the trial after the first phase are those that substantially affected the impact of the intervention in mathematical modelling simulations: prevalence of undiagnosed HIV infection before the trial, CD4 level of HIV-infected participants who are undiagnosed or diagnosed but not yet on ART before the trial, ART initiation criteria in the control arm, internal migration, test acceptance and the linkage to care. The thresholds are defined according to the results of the mathematical model (see section 8.2).

8.4.1.2 Acceptability

Clear indication from the clinic-based survey (section 7.4.2) and the qualitative in-depth interviews conducted during the first phase that the TasP approach is not acceptable in our setting. Acceptability will be assessed using a combination of quantitative and qualitative analyses of community attitudes and beliefs about HIV, HIV VCT, stigma and disclosure, participation in TasP, and the acceptability of ART for the benefit of other community members.

The DSMB will, in addition to the criteria indicated in the protocol, be able to consider additional parameters measured during the first phase such as tuberculosis incidence.

A complete “pause” between the TasP first and the full trial phase is not feasible logistically, because it would imply discontinuation of structures built during the first phase in the intervention and control communities which would need to be utilized during the full trial phase. However, detailed data analyses and interpretation will take place in half-yearly intervals throughout the first phase, ensuring that TasP trial procedures are adopted in real-time to fully take into account the lessons learned during the first phase.

8.4.2 Statistical analysis

An intention-to-treat analysis, assuming sexual partnerships occur within clusters, based on the randomisation clusters, will be used for the primary trial outcome (incidence of HIV-1 infection). Incidence rates per 100 person-years will be calculated for the whole follow-up period and compared between the intervention and control arm. Cluster randomized trials require more a complex analysis than that for individual randomised controlled trials (75). Observations on participants in the same cluster tend to be correlated; therefore it is imperative that the intra-cluster variation must be accounted for during the analysis of the trial. If this correlation is ignored in the analysis and the same techniques are employed as for individual randomised controlled trials, the associated variance of the estimate would be underestimated and lead to unduly narrow confidence intervals. To ensure valid estimates, adjusted incidence rate ratios in the intervention group relative to the control group will be based on a multi-level Poisson regression taking into account the intra-cluster correlation and the repeated measurements when needed.

In detail: we will perform analyses using cluster-level approaches as robustness analyses for checking consistency of the results. However, we will focus on Poisson regression (including random effects) for the main outcome (HIV incidence) and logistic regression with generalized estimated equation (GEE) approach for binary outcomes, thus allowing for both levels (individual and cluster levels) with easy adjustment for confounding factors (76). As the method does not perform well for a low number of clusters, we plan to use the correction (of the sandwich estimator and statistics distribution i.e. t-distribution) proposed by Mancl & Derouen (77) which is particularly appropriate when the cluster size does not vary.

Because of the possibility that the randomisation may lead to some imbalance in the distribution of risk factors across the trial arms, an adjustment for baseline characteristics known to be associated with HIV transmission risk in this population will be undertaken. These include HIV prevalence of the cluster, age, sex, marital status, education level attained and migration status.

In addition, the extensive data related to population movements and partnership patterns which will be collected over the course of the trial will be used in a parallel set of secondary analyses to identify risk factors that influenced the risk of transmission. If the intervention does not have a significant impact, these important analyses will help to identify which factors were likely to have led to the trial result.

Data analysis and preparation of papers will be led by various members of the TasP team as commensurate with their expertise. The TasP team is composed of researchers from the Africa Centre, from INSERM U897/ISPED – Bordeaux, from INSERM U108 – Paris, from INSERM U912 – Marseille, from CEPED UMR 196, from the Hôpital Cantonal in Geneva, from CEPAC and Massachusetts General Hospital – Boston and from EA 3620 – Paris.

Rules will be established to define data analysis, communication and publication plans. New concepts sheets for analyses not yet identified will be developed over the course of the trial. These documents will be developed by the SC.

8.4.3 Safety

During the trial, each HIV-infected participant receiving ART will be followed carefully within the TasP clinics, by the TasP clinic nurse and the physician "on call", in order to detect the occurrence of any adverse event. The physician will be the trial coordinator (south), specialist in HIV medicine. He will rotate around the trial clinics, with scheduled visits at each trial clinic per week, but will be available to see urgent cases, including those with adverse events, if required. In addition the TasP trial will appoint a trial nurse manager who will be a trained 'NIMART' nurse[2]: (nurse-initiated management of ART). The trial nurse manager will also rotate around the trial clinics and be available to give advice and see patients with adverse events.

This follow-up will include interviewing each patient and conducting a clinical examination to identify any change in the patient's condition or any event that has occurred since the last protocol visit. The medical management of a patient experiencing an adverse event is under the responsibility of the physician/investigator. All adverse events will be reviewed by the HIV physician and must be reported and followed until resolution.

8.5 Adverse events: definitions and reporting

An adverse event (AE) is defined as any unfavourable, expected or unexpected clinical or biological sign or symptom occurring during the trial, whether or not considered related to the trial drug or to participation in the trial.

All AE observed or reported by the patients will be recorded at each trial visit (whether scheduled or unscheduled) in the CRF, regardless of their severity and the causal relationship to the trial drug. They will not be limited to AE related to one or more drugs but will also include any signs, symptoms or defined illnesses that occur during the trial.

In the case of an AE, the investigator's responsibility is to assure proper clinical management and follow-up until resolution or stabilization. If the AE becomes worse, it should be notified and followed as stated in section 9.2 below.

All AE will be graded for severity according to the "ANRS scale to grade the severity of adverse events in adults" version 1.0 dated November 4th, 2008 (English translation of the French version 6 dated September 9th, 2003). The investigator must indicate whether, in his/her opinion, this AE could have been expected or not.

8.6 Serious adverse events: definition and reporting

8.6.1 Definition

A serious adverse event (SAE) is any untoward medical occurrence that at any dose:

[2] NIM-ART is a new initiative in SA to respond to the scale-up of ARV treatment in the country with a severe shortage of health personnel. NIMART equips nurses with the skills to diagnose, initiate and manage patients on ARVs, and is being rolled out in all provinces in SA.

- results in death
- is life-threatening
- requires hospitalisation or prolongation of existing hospitalisation
- results in persistent or significant disability or incapacity
- results in a congenital abnormality/birth defect

Within the frame of the trial the SAE also include:

- grade 4 clinical and biological events
- acute renal failure or Fanconi's syndrome

All events meeting the definition of SAE have to be reported. The SAE notification form must be completed by the investigator in charge of the patient follow-up, with due care being paid to the grading and causality.

8.6.2 Assessment of causality

All adverse events are to be assessed for the relationship to the study drug. Until proven otherwise, it should be assumed that the event is related to the study drug.

Causality will be assessed based on the following definitions:

- **Not related**: The event is clearly related to other causes, such as the clinical event of the patient or a concomitant treatment without any pharmacological interaction with the experimental drug.
- **Possibly related**: Clinical or biological event with a compatible chronological, aetiological and semi logical relation.
- **Relationship impossible to determine**: A potential causal relation between the experimental drugs and the event may exist, it may neither be affirmed nor excluded at the time of the declaration through a lack of clinical elements.

8.6.3 SAE reporting

The SAE should be reported if they occur:

- from the date of the informed consent signature for ART (even if no drugs were administered)
- during the entire duration of the trial
- up to one month after the end of the trial if the event is suspected to be related to the trial
- **and** any time after the completion of the trial if the event is suspected to be related to ART

8.6.3.1 Initial notification

The trial co-ordinator/HIV physician will notify each SAE to the trial investigator at the Africa Centre as soon as he is aware of the event. The trial co-ordinator/HIV physician will complete the trial specific "Initial Serious Adverse Event Notification" form. The form will include: study ID of participant, date of SAE and detailed description of the SAE.

The trial investigator at the Africa Centre will sends the notification to the ANRS pharmacovigilance unit within two working days of being notified of the event.

ANRS pharmacovigilance unit
Fax: +33 1 53 94 60 02
Mail: pharmacovigilance@anrs.fr

8.6.3.2 Complementary notification

The investigators or their designee are responsible for the clinical, therapeutic and biological follow-up of each SAE until resolution or stabilization.

The investigators have to report each SAE evolution (resolution, aggravation, death, final status at the end of the trial) using a "Complementary SAE Notification Form". Complementary information has to be reported in the following cases:

- systematically within 8 days in case of death and life-threatening event to clarify any relevant complementary information
- to report new information on the SAE diagnosis, its evolution, or the causal relationship to the study drug
- to answer any complementary information requested by ANRS pharmacovigilance unit

Complementary information reporting should follow the same procedure as describe above for the initial notification.

8.6.3.3 Reporting to Ethics Committee / DSMB / KZN Department of Health / Steering Committee

All SAE will be reported to the UKZN Biomedical Research Ethics Committee, the trial DSMB, the trial Steering Committee and the KwaZulu-Natal Department of Health.

8.7 Pregnancy reporting

Pregnancy is not considered as an AE or SAE. However the investigator must report all pregnancies occurring in HIV-infected women on ART during the trial on the "*Pregnancy notification form*" as soon as these are confirmed. Women of child bearing age will be asked about the possibility of being pregnant at each trial clinic visit; women who consider they may be pregnant will be offered an immediate pregnancy test

As soon, as a pregnancy becomes known, the nurse in charge of the patient should immediately report to the physician to adapt ART as needed.

Any pregnancy occurring during the trial and its outcome should be immediately reported to ANRS, using the trial "pregnancy notification form". The participant has to agree with collecting data on her pregnancy.

The investigator has to notify each pregnancy as soon as the investigator is aware to coordinating centre (same system as for SAE above, we can just say see section 9.2).

The ANRS pharmacovigilance unit reports all pregnancies in the International Antiretroviral Pregnancy Registry.

The medical surveillance of the women and their children will be reinforced, specifically regarding serious pathology occurring during pregnancy and any congenital abnormalities in the infant at delivery. A SAE initial report form should be filled if any anomaly is detected.

Any Voluntary Interruption of Pregnancy, Therapeutic interruption or miscarriage needed a hospitalisation is considered as a SAE to notify as mentioned in section 9.2 above.

All pregnancy outcomes should be reported using the “Pregnancy outcome notification form” using the same procedure described above.

8.8 Annual safety report

The ANRS pharmacovigilance unit coordinates the production of an Annual Safety Report in order to assess the benefit/risk balance throughout the duration of the trial.

This report is written in collaboration with the coordinating investigators and the clinical project manager. It is submitted to the research coordination team. It is sent to the trial coordinating team and may be used for communication with the University of KwaZulu-Natal Biomedical Research Ethics Committee and KwaZulu-Natal Department of Health.

9. Trial oversight

Figure 6 below summarises the trial oversight organisation.

Figure 6
Trial oversight

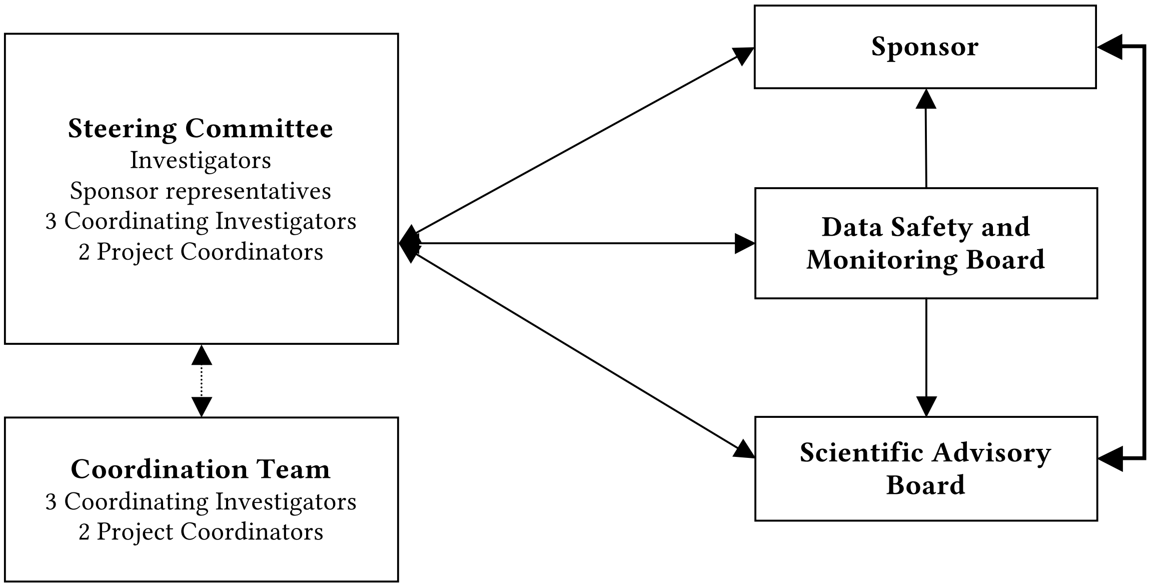

9.1 Steering Committee (SC) and Coordination Team (CT)

The **Steering Committee (SC)** will be responsible for the conduct of the trial and its overall organization. The SC is the trial decision body, for all scientific and administrative aspects. The SC will be co-chaired by the Coordinating Investigators. It will comprise the team investigators in South Africa, France and Switzerland and representatives of the Sponsor as listed on page 3 (The ANRS 12249 TasP Trial Team). The SC will meet as regularly as needed on conference calls and face-to-face meetings.

The SC ensures the correct implementation of the study and compliance with the protocol, and verifies its ethical compliance. It decides about any relevant changes to the protocol, necessary for continuation of the study.

The SC is responsible for the scientific promotion and communication of the trial data. Any sub-studies using the trial data should be discussed and approved by the SC.

The SC written report is sent to the investigators and to the Sponsor.

The Coordination Team (CT) is composed of the three Coordinating Investigators, the two Project Coordinators (one in South Africa and one in France), and any relevant participants to discuss specific issues. The CT undertakes the day-today management of the trial. The CT will meet by teleconference, which will usually be held at monthly

intervals although may be needed more frequently in the initial phases, frequency to be decided by the Project Coordinators.

The CT reports regularly to the SC for updates on trial progress and potential issues and, sends a *quarterly* progress reports (the content will be defined jointly at the beginning of the trial) to the Sponsor and to the Steering Committee.

The CT together with the SC coordinates and prepares the progress and scientific reports, communications and presentations to the SAB, DSMB, Ethical Committees and national regulatory bodies.

9.2 Data Safety Monitoring Board (DSMB)

The Data Safety Monitoring Board (DSMB) is the group that monitors the main safety and efficacy outcome measures and the overall conduct of the trial, with the aim of protecting the safety and the interests of the trial participants. The DSMB is appointed by the Sponsor before the implementation of the study and follows the current ANRS procedures “Independent Committee for ANRS sponsored clinical trials in developing countries”.

The DSMB is an independent consultative committee in charge of alerting the scientific committee, the Coordinating Investigators and the Sponsor of any modification in the trial risk benefit ratio.

The DSMB is composed of six independent experts covering the main disciplines of the trial (biostatistics, HIV adult medicine, prevention, and bioethics). All members must be free from any direct involvement with the trial; any competing interests, both real and potential, must be declared.

The DSMB meets on a regular basis (frequency is decided by the board) throughout the trial and at least once a year. In the event of a serious or unexpected problem, an extraordinary meeting may be requested by the SC, the SAB Chair or the Sponsor to discuss on questions relative to the scientific and ethical integrity of the study. The first session of the DSMB will be held prior to the implementation of the trial to agree with the trial investigators the stopping rules needed to monitor the safety issue.

The DSMB, appointed for the needs of the study, will have access to all intermediate data and results decoded by cluster, as well as to any information justifying any change affecting the course of the study. It will monitor the trial regularly with a focus on issues relating to quality of trial conduct, such as rates of recruitment, adherence to trial interventions, visit schedules, losses to follow-up, respect of ethical principles and safety data.

DSMB meetings will be organized in two parts: an open session with the trial investigators and sponsor representatives followed by closed sessions with DSMB members and the trial statistician if relevant.

DSMB written reports signed by the chair will be sent to the Coordinating Investigators, the chair of the trial SAB, the chair of the Steering Committee and the sponsor.

9.3 Scientific Advisory Board (SAB)

The Scientific Advisory Board (SAB) is the group that oversees the overall conduct of the trial. Its mission is to make sure and to report, particularly to the Sponsor, whether the study is carried out properly scientifically, ethically and logistically.

The SAB is appointed by the Sponsor before the implementation of the study.

A first meeting will be convened prior to the beginning of the trial and the following meetings will be scheduled at least once a year till the end of the trial. In the event of a serious or unexpected problem, an extraordinary meeting may be requested by the SC, the SAB Chair or the Sponsor. Meetings of the SAB will be organized whenever possible in South Africa.

The SAB formulates written recommendations to the SC and to the Sponsor. The SC is expected to give its written feed back to the SAB and the Sponsor.

10. Ethic and regulatory aspects

10.1 Ethics and competent authorities

This trial will be conducted in compliance with the protocol and with the following:

- the ethical principles outlined in the most recent version of Declaration of Helsinki
- the guideline for Good Clinical Practice (ICH E6 May 1996)
- the ANRS Ethics charter for research in developing countries (May 2002, amended October 2008)
- in accordance with the approval from the University of KwaZulu-Natal Biomedical Research Ethics Committee
- the Research Committee of the KwaZulu-Natal Department of Health
- and approval of the local population through the Africa Centre Community Advisory Board.

The trial protocol was submitted for approval to the Biomedical Research Ethics Committee of the University of KwaZulu-Natal (see Appendix 15.7) and for health authorities' authorisation to the KwaZulu-Natal Department of Health in South Africa.

This final version of this protocol was approved and signed by the three Coordinating Investigators and the Sponsor (cf. signatures on page 1).

10.1.1 Africa Centre Community Advisory Board (CAB)

The Africa Centre's Community Advisory Board (CAB) is an autonomous body consisting of approximately 30 members chosen by the community, whose main role is to link the Africa Centre with the community. The CAB membership comprises representatives from the traditional authorities, local councillors, community members and representative from the local offices of key provincial government departments including Health, Education and Social Development. Through the CAB, community input will be provided into trial design (cohort selection criteria), questionnaire content, participant follow-up plans, informed consent procedures, risk reduction interventions, community education and outreach, recruitment, retention planning and dissemination of trial findings. All research initiatives at the Africa Centre are presented to the CAB for discussion before seeking ethics approval from the University of KwaZulu-Natal's Ethics Committee.

10.1.2 Biomedical Research Ethics Committee of the University of KwaZulu-Natal

The Biomedical Research Ethics Committee is mandated to fulfil its function by the Senate of the University of KwaZulu-Natal. The essential function of the Committee is to review the protocols of all human subjects health research projects proposed to be undertaken by students and members of staff of the University. The purpose of this review is the protection of the dignity, rights, safety and well-being of all human participants of research. Special attention is given to research that may include vulnerable participants. The Committee is available to review, advise on, and approve or reject research protocols involving human participants submitted to it by researchers. Research to be reviewed will be in accordance with the stipulations of the National Health Act of the Republic of South Africa.

The membership of the Committee comprises of a Chairperson, two Deputy Chairs, at least two laypersons with no affiliations with the institution (preferably from the community), at least one member with knowledge of and current experience in research areas that are regularly considered by the Committee, at least two members with knowledge of and current experience in professional care, counselling or treatment (e.g. general practitioner, psychologist, etc.), at least one member who is legally trained. Decisions are made at meetings at which at least a quorum of 50 % plus one is present.

10.1.3 KwaZulu-Natal Department of Health (DoH)

The mission of the KwaZulu-Natal Provincial DoH is to develop a sustainable, co-ordinated, integrated and comprehensive health system at all levels, based on the primary health care approach through the district health system. In total there are 11 health districts in the province, with Africa Centre situated in the Umkhanyakude District. The District has five district hospitals. The hospital doctors are general practitioners, many with special interests. Despite not having specialist posts, the hospitals all perform surgery including caesarean sections, and offer excellent opportunities for surgical and anaesthetic experience. Supported by the hospitals are 52 residential clinics staffed with primary health care nurses. Most of these clinics are visited twice monthly by medical and paramedical staff. Since 2004 the Africa Centre has partnered with the DoH in the Hlabisa sub-District in the roll-out of antiretroviral treatment, with the financial support from PEPFAR. The Africa Centre's support has focused on the Hlabisa hospital and its 17 peripheral clinics. The Africa Centre provides assistance with infrastructure, training and transfer of skill; including management skills. The focus of the Africa Centre's HIV Treatment and Care Programme has always been one of building sustainability. The DoH has provided a letter of support to the TasP project (see Appendix 15.3).

10.2 Community, participant and patient information and consent

Community engagement and provision of adequate patient/participant information are keys in the success of the trial. The KwaZulu-Natal DoH has developed and approved an extensive array of community and patient information materials relating to all aspects of HIV – prevention, treatment and care. These are already widely available in the clinics and will be made available in the trial clinics as well. The social science team, with the community liaison office, is developing other information and community education materials for use in the trial as part of the initial preparatory work started in 2010. Examples of the materials being prepared include a community information leaflet (see Appendix 15.4), and the referral cards given to all participants during the home-based testing rounds. The four community advisory panels described above and Africa Centre Community Liaison Office will also contribute to the development of community, participant or patient information materials to ensure their appropriateness. Development and provision of any printed materials will follow normal approval processes in respect to their scientific content, input from trial Steering Committee, trial ethics and the provincial DoH as appropriate.

We will use the routine community road shows that are part of the Hlabisa Treatment and Care Programme's activities to ensure that there is a continuous feedback loop between the investigators and the communities. Over the years these road shows have been demonstrated to be a highly effective communication tools to promote HIV testing and treatment, and to educate the communities about the range of services and options

provided by the programme. The content of any materials to be disseminated through the road shows will be approved by the trial investigators.

Consent for the collection of data and the retention of residual samples will be collected from individuals following the procedures of the Africa Centre and approved by the University of KwaZulu-Natal Biomedical Research Ethics Committee. Consent forms are presented in appendix 15.4. In each instance when consent is sought an individual will be fully briefed by the counsellor or clinical staff member and asked to provide a signature, the signature will then be countersigned by the staff member with the inclusion of a date time. For participants unable to write their own name, they will be asked to 'make their mark' (customarily an 'X'). In the event that a participant needs to do this the counsellor will need to have a second witness, normally another household member or Africa Centre counsellor, to verify the mark. This will only apply during the home-based testing rounds and when a person is not in possession of a South African national identity document. For participants that attend trial clinics for treatment and care, who are unable to write and do not possess a South African national identity document the process of making a mark/providing a thumb-print will be witnessed by two clinic staff members, unless the person attends with a 'treatment supporter' or family member, to avoid the potential for inadvertent disclosure of HIV status.

10.3 Data confidentiality

Current Africa Centre procedures require all staff to sign confidentiality and Acceptable Use Policy agreements, stating they have read and understood them and that they agree to abide by them. There are also formal policies in place regarding e.g. password usage and email security.

Each trial subject will be identified by a unique trial number and this alone will be used throughout the trial to identify the participant.

Personal identification details (Name, Id. No, clinic numbers, address, etc.) will only be made available to a) those whose job within the operational activities of the trial makes having such information absolutely essential, and b) to senior members of the trial administration (coordinating investigators, ANRS trial monitors, DSMB statistician) at the discretion of the trial coordinating investigators.

Completed questionnaires will be stored in a secure environment (in locked cabinets within the secure, access-controlled, Data Centre) and access will be granted only on a 'need to know' basis. These completed questionnaires and other study-related documents will be digitally archived according to the SOP developed in the AC for this purpose following strict quality control procedures.

Staff will be required (and trained) to always email personal information in password-protected attachments, not in the body of email, nor in unprotected attachments. Similarly, transmission of personal information via text (SMS) or fax messages, even for valid operational purposes, will be forbidden.

Data used for analysis, as opposed to day-to-day operational activities, will never contain personal identifying information, and will only be issued under a formal Data Use Agreement which must be signed by the data user, the Africa Centre Director, and the trial coordinating investigators. It imposes conditions on data use including that data will be stored securely, will not be passed to others, used only for the agreed purposes, and that it will be destroyed after completion of the agreed analyses, as per Africa Centre standard procedures.

10.4 Protocol amendments

Any substantial change to the protocol will be described in an amendment, which will be ratified by the trial SC, and forwarded to the sponsor for agreement and to the pharmaceutical company partners in the trial (where appropriate) for information and comments. These amendments will then be forwarded to the Biomedical Research Ethics Committee of the University of KwaZulu-Natal for approval. Any amendment should be signed by the Sponsor, the Coordinating Investigators and the Biomedical Research Ethics Committee of the University of KwaZulu-Natal before its implementation.

10.5 Investigators responsibilities

The Coordinating Investigators agree to conduct this trial in full accordance with the provisions of this protocol and will comply with all requirements regarding the obligations of clinical investigators as outlined in the ICH – E6 good clinical practice (GCP) guidelines. They agree to maintain all trial documentation until the trial sponsor consents to disposal of files in writing. They are fully aware of the potential risks and side effects of the products under investigation, and will ensure that all associates, colleagues, and employees assisting in the conduct of the trial are informed about the obligations incurred by their contribution to the trial.

10.6 Insurance

The ANRS, as the Sponsor, will take a civil liability insurance policy, for the trial to cover all HIV infected patients receiving ARV treatment. A copy of the insurance certificate is given in Appendix 15.6.

11. Data management and monitoring

11.1 Data collection and storage of forms

Questionnaires, electronic or paper-based CRFs will be used for data collection on site. The questionnaires and CRFs will be designed, completed, stored and relayed according to GCP principles and using local accredited GCP trainers. The completed questionnaires, CRFs and supporting documentation will be kept securely, in locked cabinets at the Africa Centre location, as trial source documents for potential review and/or audit during or after the trial. No source documents will be destroyed without specific permission in writing from the project coordinators. Only the coordinating investigators, the project coordinators and the authorised trial personnel will have access to the completed questionnaires, CRFs and supporting documents. Data capture and storage will be undertaken using computer systems compliant to GCP. To ensure correct operation according to SOP all system users will be trained and evaluated on a regular basis in line with Africa Centre policy. In-country and external monitoring will be organized to ensure that all the trial procedures are respected on site and to verify the data validity and reliability.

11.2 Data management

The Africa Centre Information Technology (IT) network is a technologically advanced Microsoft-based setup, professionally designed and maintained according to Microsoft 'best practices', by a large South African IT services company in collaboration with the Africa Centre IT Manager and his staff. Servers (mostly virtualised) are all located at the Africa Centre in a secure computer room and the network is protected by uninterrupted power supply (UPS) firewalls, up-to-date virus and malwares scanning software. Users are all given their own logins and sign confidentiality and 'Acceptable Use Policy' agreements. A comprehensive system of backups and archiving is in place, with some held off-site (transported and stored by a professional security company). A disaster recovery plan is in place and reviewed regularly.

The data collection, entry and management for the trial will be modelled closely on the current practices and procedures for existing Africa Centre surveys and trials for which a large suite of SOPs and policy documents exist (see www.africacentre.com) and which have been developed over more than 10 years of field operations. Many of the staff involved have considerable experience of managing clinical trials and the surrounding business processes as well as our surveillance systems and various demographic and health surveys. Currently the Data Centre prints and processes about 1.2 million pages per annum.

Every questionnaire will be individually bar-coded, with its details recorded in a database, and scanned at each stage of its life from printing and issue to fieldworkers through to final archiving, hence achieving an end-to-end 'chain of ownership' and clear visibility of where every form is at any moment. All completed forms will, on return from the field, first be checked visually by our Quality Control Department before being passed to staff in our secure Data Centre for data entry, and subsequent digital scanning and archiving. All entered data will be carefully checked for consistency and accuracy and, if necessary, correction.

All data will be stored in a MS-SQL Server database located on one of our own database servers, managed by professional Database Administrators. Access to read, enter, modify or delete data will be granted via the standard authentication and access-control

features of MS-SQL Server and MS-Windows. Laboratory results will be transmitted, using already-established procedures, directly into the database, via a secure (https) connection from the Africa Centre Laboratory's Laboratory Information System. Data issued for analysis by scientists will all be anonymised and covered by formal, signed Data Use Agreements, which cover acceptable use, security, destruction after use etc.

Overall, the Africa Centre offers a very high quality, tried and tested professional data collection and management service, conforming to good clinical practice and trial specifications. Compliance with the ANRS rules and regulations will be verified before trial start.

11.3 Exchange of data

The trial data are stored at the Africa Centre. All members of the SC can have access to specific trial data as per their research interests, as appropriate and following agreed procedures. Requests for data for specific analyses must be accompanied by a data analysis plan, as well as, if required, ethics certification by recognised ethics committees abroad. All requests for analysis will need to be discussed and approved by the SC.

Any exchange of trial data will be through secure connections, password-protected (see trial SOPs for further details).

11.4 Quality assurance, monitoring, audits and inspections

All trial procedures will be standardised and compiled in a manual of operations. A training session will be scheduled before the trial starts for all trial staff.

In accordance with Good Clinical Practice (GCP) to ensure the quality of the trial, each investigator accepts monitoring visits, audits and inspections.

11.4.1 Monitoring

A **Trial Initiation Visit** will take place before the first patient enrolment in the trial. During this visit, the monitor/monitoring team (from UKZN clinical trials centre or from ANRS) will review the trial material: documents compiled in an Investigator file, trial products and will verify that the investigational team understands the protocol and GCP requirements.

During the trial, a representative of the Africa Centre/monitoring team will make regular **Monitoring Visits** to the clinical service, hospital pharmacy and laboratories involved in the trial to (i) ensure that the study is conducted according to the protocol and GCP and (ii) to help the investigational team in solving problems. During these monitoring visits, the monitor will have access to the source document for patient validation data reported in CRF (at any time, the investigator or his representative can be contacted for any matter relating to the protocol, its practical application or the measures to take facing certain events).

Once all participants have been enrolled, and all study procedures have been completed, and all clinical data has been duly recorded in the CRFs and reported to the Sponsor, a **Close-out Visit** must be done by the monitor, to ensure that the Investigator File and other trial documents are archived properly; in addition, the monitor must collect all unused trial material, documents and products.

The monitor will submit a written report to the sponsor or its representative after each trial-site visit or trial-related communication.

11.4.2 Audits and inspections

The trial may be audited by the Sponsor or with his express authorization by other agencies.

The purpose of a sponsor's audit, which is independent of and separate from routine monitoring or quality control functions, is to be able to evaluate trial conduct and compliance with the protocol, SOPs, GCP, and the applicable regulatory requirements.

12. Archiving

The investigators on site will digitally archive the patient information sheet, informed consent forms, protocol and amendment(s), CRF model, correspondences, patients' CRF and sources data/documents and will keep this information for 15 years at the Africa Centre, in accordance with GCP for clinical trials.

13. Results publications

The investigator team will meet to define the core publications from the first phase, responsibility for overseeing additional papers and plans for analysis (including the core papers) will be overseen by a publication committee.

The main trial results on the effectiveness of 'treatment as prevention' must receive prior approval from the coordinating investigators and SC in respect of the agreement contracted between ANRS and the pharmaceutical company, which provided drugs.

The publication of the main trial results should include the name of the sponsor, all the investigators who included or followed-up participants in the trial, the composition of the SC and the possible participation of pharmaceutical company, which provided drugs. Each publication in a scientific journal or subjected to a scientific conference or for the media should include:

- that the sponsor is Inserm-ANRS: "*French National Institute of Health and Medical Research - French National Agency for Aids and Viral Hepatitis Research (Inserm-ANRS) is the sponsor of the TasP trial.*";
- the source of funding if co-financing and/or if different from the sponsoring ("*French National Institute of Health and Medical Research - French National Agency for Aids and Viral Hepatitis Research (Inserm-ANRS) and the Deutsche Gesellschaft für Internationale Zusammenarbeit (GIZ) funded TasP phase 1*" and "*The French National Institute of Health and Medical Research - French National Agency for Aids and Viral Hepatitis Research (Inserm-ANRS) and the International Initiative for Impact Evaluation (3ie) funded TasP phase 2*"
- the pharmaceutical company which provided drugs ("*Trial conducted with the support of MERCK & Co. Inc and Gilead Sciences.*").

14. References

1. Merson MH, O'Malley J, Serwadda D, Apisuk C. The history and challenge of HIV prevention. Lancet. 2008 Aug 9;372(9637):475-88.

2. Rerks-Ngarm S, Pitisuttithum P, Nitayaphan S, Kaewkungwal J, Chiu J, Paris R, et al. Vaccination with ALVAC and AIDSVAX to prevent HIV-1 infection in Thailand. N Engl J Med. 2009 Dec 3;361(23):2209-20.

3. Skoler-Karpoff S, Ramjee G, Ahmed K, Altini L, Plagianos MG, Friedland B, et al. Efficacy of Carraguard for prevention of HIV infection in women in South Africa: a randomised, double-blind, placebo-controlled trial. Lancet. 2008 Dec 6;372(9654):1977-87.

4. Abdool Karim Q, Abdool Karim SS, Frohlich JA, Grobler AC, Baxter C, Mansoor LE, et al. Effectiveness and safety of tenofovir gel, an antiretroviral microbicide, for the prevention of HIV infection in women. Science. 2010 Sep 3;329(5996):1168-74.

5. Gray RH, Wawer MJ. Reassessing the hypothesis on STI control for HIV prevention. Lancet. 2008 Jun 21;371(9630):2064-5.

6. Taylor MM, Aynalem G, Olea LM, He P, Smith LV, Kerndt PR. A consequence of the syphilis epidemic among men who have sex with men (MSM): neurosyphilis in Los Angeles, 2001-2004. Sex Transm Dis. 2008 May;35(5):430-4.

7. Ngubane N, Patel D, Newell ML, Coovadia HM, Rollins N, Coutsoudis A, et al. Messages about dual contraception in areas of high HIV prevalence are not heeded. S Afr Med J. 2008 Mar;98(3):209-12.

8. Chimbindi NZ, McGrath N, Herbst K, San Tint K, Newell ML. Socio-Demographic Determinants of Condom Use Among Sexually Active Young Adults in Rural KwaZulu-Natal, South Africa. Open AIDS J. 2010;4:88-95.

9. Quinn TC, Wawer MJ, Sewankambo N, Serwadda D, Li C, Wabwire-Mangen F, et al. Viral load and heterosexual transmission of human immunodeficiency virus type 1. Rakai Project Study Group. N Engl J Med. 2000 Mar 30;342(13):921-9.

10. Garcia PM, Kalish LA, Pitt J, Minkoff H, Quinn TC, Burchett SK, et al. Maternal levels of plasma human immunodeficiency virus type 1 RNA and the risk of perinatal transmission. Women and Infants Transmission Study Group. N Engl J Med. 1999 Aug 5;341(6):394-402.

11. Attia S, Egger M, Muller M, Zwahlen M, Low N. Sexual transmission of HIV according to viral load and antiretroviral therapy: systematic review and meta-analysis. Aids. 2009 Jul 17;23(11):1397-404.

12. Cohen MS, Chen YQ, McCauley M, Gamble T, Hosseinipour MC, Kumarasamy N, et al. Prevention of HIV-1 Infection with Early Antiretroviral Therapy. N Engl J Med. 2011 Jul 18.

13. Kitahata MM, Gange SJ, Abraham AG, Merriman B, Saag MS, Justice AC, et al. Effect of early versus deferred antiretroviral therapy for HIV on survival. N Engl J Med. 2009 Apr 30;360(18):1815-26.

14. Sterne JA, May M, Costagliola D, de Wolf F, Phillips AN, Harris R, et al. Timing of initiation of antiretroviral therapy in AIDS-free HIV-1-infected patients: a collaborative analysis of 18 HIV cohort studies. Lancet. 2009 Apr 18;373(9672):1352-63.

15. Severe P, Juste MA, Ambroise A, Eliacin L, Marchand C, Apollon S, et al. Early versus standard antiretroviral therapy for HIV-infected adults in Haiti. N Engl J Med. 2010 Jul 15;363(3):257-65.

16. Danel C, Moh R, Minga A, Anzian A, Ba-Gomis O, Kanga C, et al. CD4-guided structured antiretroviral treatment interruption strategy in HIV-infected adults in west Africa (Trivacan ANRS 1269 trial): a randomised trial. Lancet. 2006 Jun 17;367(9527):1981-9.

17. Hargrove JW, Humphrey JH, Zvitambo Study Grp. Mortality among HIV-positive postpartum women with high CD4 cell counts in Zimbabwe. Aids. [Article]. 2010 Jan;24(3):F11-F4.

18. World Health Organisation. Antiretroviral therapy for HIV infection in adults and adolescents. Recommendations for a public health approach (2010 revision). Geneva: World Health Organisation 2010. Available from: http://whqlibdoc.who.int/publications/2010/9789241599764_eng.pdf. [Access date 18/01/2013].

19. World Health Organisation. Consolidated guidelines on the use of antiretroviral drugs for treating and preventing HIV infection. Recommendations for a public health approach. Geneva: World Health Organisation 2013. Available from: http://www.who.int/iris/bitstream/10665/85321/1/9789241505727_eng.pdf. [Access date 26/07/2013].

20. Le Coeur S, Collins IJ, Pannetier J, Lelievre E. Gender and access to HIV testing and antiretroviral treatments in Thailand: Why women have more and earlier access? Soc Sci Med. 2009 Jun 30;69(6):846-53.

21. UNAIDS. The impact of Voluntary Counselling and Testing. a global review of the benefits and challenges. Geneva: UNAIDS 2001. Report No.: UNAIDS/01.32E. Available from: http://www.unaids.org/publications/documents/health/counselling/JC580-VCT-E.pdf.

22. Suthar AB, Ford N, Bachanas PJ, Wong VJ, Rajan JS, Saltzman AK, et al. Towards universal voluntary HIV testing and counselling: a systematic review and meta-analysis of community-based approaches. PLoS Med. 2013 Aug;10(8):e1001496.

23. Granich RM, Gilks CF, Dye C, De Cock KM, Williams BG. Universal voluntary HIV testing with immediate antiretroviral therapy as a strategy for elimination of HIV transmission: a mathematical model. Lancet. 2009 Jan 3;373(9657):48-57.

24. Garnett GP, Baggaley RF. Treating our way out of the HIV pandemic: could we, would we, should we? Lancet. 2009 Jan 3;373(9657):9-11.

25. De Cock KM, Gilks CF, Lo YR, Guerma T. Can antiretroviral therapy eliminate HIV transmission? Lancet. 2009 Jan 3;373(9657):7-9.

26. Dodd PJ, Garnett GP, Hallett TB. Examining the promise of HIV elimination by 'test and treat' in hyperendemic settings. AIDS. 2010 Feb 11;24(5):729-35.

27. Shisana O, editor. HIV/AIDS in South Africa: At last the glass is half full. 6th SA AIDS Conference, 18-21 June; 2013; Durban, South Africa.

28. National Department of Health. HIV & AIDS and STI National Strategic Plan 2012-2017: Department of Health, Republic of South Africa 2012.

29. National Department of Health. The South African antiretroviral treatment guidelines: Department of Health, Republic of South Africa 2013. Available from:

http://www.doh.gov.za/docs/policy/2013/ART_Treatment_Guidelines_Final_25March2013.pdf.

30. Houlihan CF, Bland RM, Mutevedzi PC, Lessells RJ, Ndirangu J, Thulare H, et al. Cohort Profile: Hlabisa HIV Treatment and Care Programme. Int J Epidemiol. 2010 Feb 12;[Epub ahead of print].

31. Mutevedzi PC, Lessells RJ, Heller T, Barnighausen T, Cooke GS, Newell ML. Scale-up of a decentralized HIV treatment programme in rural KwaZulu-Natal, South Africa: does rapid expansion affect patient outcomes? Bull World Health Organ. 2010 Aug 1;88(8):593-600.

32. Lessells RJ, Mutevedzi PC, Iwuji CC, Newell ML. Reduction in early mortality on antiretroviral therapy for adults in rural South Africa since change in CD4+ cell count eligibility criteria. J Acquir Immune Defic Syndr. 2013 Jun 10.

33. National Department of Health. Clinical guidelines for the management of HIV/AIDS in adults and adolescents: Department of Health, Republic of South Africa 2010.

34. Bärnighausen T, Hosegood V, Timaeus IM, Newell ML. The socioeconomic determinants of HIV incidence: evidence from a longitudinal, population-based study in rural South Africa. AIDS. 2007 Nov;21 Suppl 7:S29-38.

35. Barnighausen T, Wallrauch C, Welte A, McWalter TA, Mbizana N, Viljoen J, et al. HIV incidence in rural South Africa: comparison of estimates from longitudinal surveillance and cross-sectional cBED assay testing. PLoS ONE. 2008;3(11):e3640.

36. Barnighausen T, McWalter TA, Rosner Z, Newell ML, Welte A. HIV incidence estimation using the BED capture enzyme immunoassay: systematic review and sensitivity analysis. Epidemiology. 2010 Sep;21(5):685-97.

37. Duracinsky M, A.C., Lalanne C, Hermann S, Lau L, Lecoeur S, et al., editors. Differences in Heath-related Quality of life (HRQL) measured by the PROQOL-HIV, a new specific instrument developed across cultures. 5th IAS Conference on HIV Pathogenesis Treatment and Prevention; 2009; Cape Town, South Africa.

38. Duracinsky M, L.C., Acquadro C, Hermann S, Lau L, Lecoeur S, et al., editors. Psychometric validation of PROQOL-HIV a contemporaneous and cross-cultural Health-Related Quality-of-Life (HRQL) Questionnaire specific to HIV. 5th IAS Conference on HIV Pathogenesis Treatment and Prevention; 2009; Cape Town, South Africa.

39. Holzemer WL, Uys LR, Chirwa ML, Greeff M, Makoae LN, Kohi TW, et al. Validation of the HIV/AIDS Stigma Instrument - PLWA (HASI-P). AIDS Care. 2007 Sep;19(8):1002-12.

40. Cleary S, Mooney G, McIntyre D. Equity and efficiency in HIV-treatment in South Africa: the contribution of mathematical programming to priority setting. Health Econ. 2010 Oct;19(10):1166-80.

41. Bender DE, Baker R, Dusch E, McCann MF. Integrated use of qualitative and quantitative methods to elicit women's differential knowledge of breastfeeding and lactational amenorrhea in periurban Bolivia. J Health Popul Dev Ctries. 1997;1(1):68-84.

42. Cleary SM, McIntyre D, Boulle AM. Assessing efficiency and costs of scaling up HIV treatment. AIDS. 2008 Jul;22 Suppl 1:S35-42.

43. Hughes J, Jelsma J, Maclean E, Darder M, Tinise X. The health-related quality of life of people living with HIV/AIDS. Disabil Rehabil. 2004 Mar 18;26(6):371-6.

44. Chaiyachati K, Hirschhorn LR, Tanser F, Newell ML, Barnighausen T. Validating five questions of antiretroviral nonadherence in a public-sector treatment program in rural South Africa. AIDS Patient Care STDS. 2010 Mar;25(3):163-70.

45. Boyer S, Clerc I, Bonono CR, Marcellin F, Bile PC, Ventelou B. Non-adherence to antiretroviral treatment and unplanned treatment interruption among people living with HIV/AIDS in Cameroon: Individual and healthcare supply-related factors. Soc Sci Med. 2011 Apr;72(8):1383-92.

46. Carrieri P, Cailleton V, Le Moing V, Spire B, Dellamonica P, Bouvet E, et al. The dynamic of adherence to highly active antiretroviral therapy: results from the French National APROCO cohort. J Acquir Immune Defic Syndr. 2001 Nov 1;28(3):232-9.

47. Spire B, Carrieri P, Sopha P, Protopopescu C, Prak N, Quillet C, et al. Adherence to antiretroviral therapy in patients enrolled in a comprehensive care program in Cambodia: a 24-month follow-up assessment. Antivir Ther. 2008;13(5):697-703.

48. Oyugi JH, Byakika-Tusiime J, Ragland K, Laeyendecker O, Mugerwa R, Kityo C, et al. Treatment interruptions predict resistance in HIV-positive individuals purchasing fixed-dose combination antiretroviral therapy in Kampala, Uganda. AIDS. 2007 May 11;21(8):965-71.

49. Ndirangu J, Newell ML, Tanser F, Herbst AJ, Bland R. Decline in early life mortality in a high HIV prevalence rural area of South Africa: evidence of HIV prevention or treatment impact? Aids. 2010 Feb 20;24(4):593-602.

50. Van Damme W, Kober K, Laga M. The real challenges for scaling up ART in sub-Saharan Africa. Aids. 2006 Mar 21;20(5):653-6.

51. Harries AD, Schouten EJ, Libamba E. Scaling up antiretroviral treatment in resource-poor settings. Lancet. 2006 Jun 3;367(9525):1870-2.

52. Rowe AK, de Savigny D, Lanata CF, Victora CG. How can we achieve and maintain high-quality performance of health workers in low-resource settings? Lancet. 2005 Sep 17-23;366(9490):1026-35.

53. Souville M, Msellati P, Carrieri MP, Brou H, Tape G, Dakoury G, et al. Physicians' knowledge and attitudes toward HIV care in the context of the UNAIDS/Ministry of Health Drug Access Initiative in Cote d'Ivoire. Aids. 2003 Jul;17 Suppl 3:S79-86.

54. Adam MA, Johnson LF. Estimation of adult antiretroviral treatment coverage in South Africa. S Afr Med J. 2009;99(9):661-7.

55. Tanser F, Lesueur D, Solarsh G, Wilkinson D. HIV heterogeneity and proximity of homestead to roads in rural South Africa: an exploration using a geographical information system. Trop Med Int Health. 2000 Jan;5(1):40-6.

56. Hayes R, Moulton LH. Cluster randomised trials. Boca Raton, FL: CRC Press; 2009.

57. Hontelez JA, de Vlas SJ, Baltussen R, Newell ML, Bakker R, Tanser F, et al. The impact of antiretroviral treatment on the age composition of the HIV epidemic in sub-Saharan Africa. AIDS. 2012 Jul 31;26 Suppl 1:S19-30.

58. Hontelez JA, de Vlas SJ, Tanser F, Bakker R, Barnighausen T, Newell ML, et al. The impact of the new WHO antiretroviral treatment guidelines on HIV epidemic dynamics and cost in South Africa. PLoS ONE. 2011;6(7):e21919.

59. Hontelez JA, Lurie MN, Newell ML, Bakker R, Tanser F, Barnighausen T, et al. Ageing with HIV in South Africa. AIDS. 2011 Aug 24;25(13):1665-7.

60. Korenromp EL, Bakker R, De Vlas SJ, Robinson NJ, Hayes R, Habbema JD. Can behavior change explain increases in the proportion of genital ulcers attributable to herpes in sub-Saharan Africa? A simulation modeling study. Sex Transm Dis. 2002 Apr;29(4):228-38.

61. Korenromp EL, Bakker R, Gray R, Wawer MJ, Serwadda D, Habbema JDF. The effect of HIV, behavioural change, and STD syndromic management on STD epidemiology in sub-Saharan Africa: simulations of Uganda. Sex Transm Infect. 2002;78:I55-I63.

62. Korenromp EL, Van Vliet C, Bakker R, de Vlas SJ, Habbema JD. HIV spread and partnership reduction for different patterns of sexual behavior – a study with the microsimulation model STDSIM. Mathematics Pop Stud. 2000;8:135-73.

63. Korenromp EL, Van Vliet C, Grosskurth H, Gavyole A, Van der Ploeg CP, Fransen L, et al. Model-based evaluation of single-round mass treatment of sexually transmitted diseases for HIV control in a rural African population. AIDS. 2000 Mar 31;14(5):573-93.

64. Orroth KK, Freeman EE, Bakker R, Buve A, Glynn JR, Boily MC, et al. Understanding the differences between contrasting HIV epidemics in east and west Africa: results from a simulation model of the Four Cities Study. Sexually Transmitted Infections. 2007;83 Suppl. 1:I5-I16.

65. Orroth KK, White RG, Korenromp EL, Bakker R, Changalucha J, Habbema JD, et al. Empirical observations underestimate the proportion of human immunodeficiency virus infections attributable to sexually transmitted diseases in the Mwanza and Rakai sexually transmitted disease treatment trials: Simulation results. Sex Transm Dis. 2006 Sep;33(9):536-44.

66. Schmidt WP, Van Der Loeff MS, Aaby P, Whittle H, Bakker R, Buckner M, et al. Behaviour change and competitive exclusion can explain the diverging HIV-1 and HIV-2 prevalence trends in Guinea-Bissau. Epidemiol Infect. 2008 Apr;136(4):551-61.

67. van der Ploeg CPB, Van Vliet C, de Vlas SJ, Ndinya Achola JO, Fransen L, Van Oortmarssen GJ. STDSIM: A Microsimulation Model for Decision Support in STD Control. Interfaces. 1998;28(3):84-100.

68. Vissers DC, SJ DEV, Bakker R, Urassa M, Voeten HA, Habbema JD. The impact of mobility on HIV control: a modelling study. Epidemiol Infect. 2011 Dec;139(12):1845-53.

69. White RG, Freeman EE, Orroth KK, Bakker R, Weiss HA, O'Farrell N, et al. Population-level effect of HSV-2 therapy on the incidence of HIV in sub-Saharan Africa. Sex Transm Infect. 2008 Oct;84 Suppl 2:ii12-8.

70. White RG, Glynn JR, Orroth KK, Freeman EE, Bakker R, Weiss HA, et al. Male circumcision for HIV prevention in sub-Saharan Africa: who, what and when? AIDS. 2008 Sep 12;22(14):1841-50.

71. White RG, Orroth KK, Glynn JR, Freeman EE, Bakker R, Habbema JD, et al. Treating curable sexually transmitted infections to prevent HIV in Africa: still an effective control strategy? J Acquir Immune Defic Syndr. 2008 Mar 1;47(3):346-53.

72. van Vliet C, Meester EI, Korenromp EL, Singer B, Bakker R, Habbema JD. Focusing strategies of condom use against HIV in different behavioural settings: an evaluation based on a simulation model. Bull World Health Organ. 2001;79(5):442-54.

73. Hontelez JA, Nagelkerke N, Barnighausen T, Bakker R, Tanser F, Newell ML, et al. The potential impact of RV144-like vaccines in rural South Africa: a study using the STDSIM microsimulation model. Vaccine. 2011 Aug 18;29(36):6100-6.

74. Tanser F, Barnighausen T, Grapsa E, Zaidi J, Newell ML. High coverage of ART associated with decline in risk of HIV acquisition in rural KwaZulu-Natal, South Africa. Science. 2013 Feb 22;339(6122):966-71.

75. Hayes RJ, Alexander ND, Bennett S, Cousens SN. Design and analysis issues in cluster-randomized trials of interventions against infectious diseases. Stat Methods Med Res. 2000 Apr;9(2):95-116.

76. Ukoumunne OC, Carlin JB, Gulliford MC. A simulation study of odds ratio estimation for binary outcomes from cluster randomized trials. Stat Med. 2007 Aug 15;26(18):3415-28.

77. Mancl LA, DeRouen TA. A covariance estimator for GEE with improved small-sample properties. Biometrics. 2001 Mar;57(1):126-34.

78. Riddler SA, Haubrich R, DiRienzo AG, Peeples L, Powderly WG, Klingman KL, et al. Class-sparing regimens for initial treatment of HIV-1 infection. N Engl J Med. 2008 May 15;358(20):2095-106.

79. Pozniak AL, Gallant JE, DeJesus E, Arribas JR, Gazzard B, Campo RE, et al. Tenofovir disoproxil fumarate, emtricitabine, and efavirenz versus fixed-dose zidovudine/lamivudine and efavirenz in antiretroviral-naive patients: virologic, immunologic, and morphologic changes--a 96-week analysis. J Acquir Immune Defic Syndr. 2006 Dec 15;43(5):535-40.

80. Bera E, McCausland K, Nonkwelo R, Mgudlwa B, Chacko S, Majeke B. Birth defects following exposure to efavirenz-based antiretroviral therapy during pregnancy: a study at a regional South African hospital. AIDS. 2010 Jan 16;24(2):283-9.

81. Lima VD, Johnston K, Hogg RS, Levy AR, Harrigan PR, Anema A, et al. Expanded access to highly active antiretroviral therapy: a potentially powerful strategy to curb the growth of the HIV epidemic. J Infect Dis. 2008 Jul 1;198(1):59-67.

82. Mugyenyi P, Walker AS, Hakim J, Munderi P, Gibb DM, Kityo C, et al. Routine versus clinically driven laboratory monitoring of HIV antiretroviral therapy in Africa (DART): a randomised non-inferiority trial. Lancet. 2010 Jan 9;375(9709):123-31.

83. Eron J, Andrade J, Zajdenverg R, Workman C, Cooper D, Young B, et al. Switching from Stable Lopinavir/Ritonavir-based to Raltegravir-based Combination ART Resulted in a Superior Lipid Profile at Week 12 but Did Not Demonstrate Non-Inferior Virologic Efficacy at Week 24. 16th Conference on Retroviruses and Opportunistic Infections; February 8-11; Montreal2009.
